# ClusterGraph: a new tool for visualisation and compression of multidimensional data

**DOI:** 10.1093/gigascience/giag070

**Published:** 2026-06-13

**Authors:** Paweł Dłotko, Davide Gurnari, Mathis Hallier, Anna Jurek-Loughrey

**Affiliations:** Center of Trustworthy AI for Life Sciences – International Research Agendas Programme, Warsaw University, 00-927, Warsaw, Poland; Dioscuri Centre in Topological Data Analysis, Mathematical Institute, Polish Academy of Sciences, 00-656, Warsaw, Poland; Dioscuri Centre in Topological Data Analysis, Mathematical Institute, Polish Academy of Sciences, 00-656, Warsaw, Poland; Dioscuri Centre in Topological Data Analysis, Mathematical Institute, Polish Academy of Sciences, 00-656, Warsaw, Poland; Génie Informatique, Université de Technologie de Compiègne, 60200, Compiègne, France; School of Electronics, Electrical Engineering and Computer Science, Queens University of Belfast, BT9 5BN, Belfast, UK

**Keywords:** visualization, mapper algorithm, scRNA-seq, bioinformatics, clusters analysis, data compression, large-scale datasets, data exploration, high-throughput data analysis

## Abstract

**Background:**

Understanding the organization of high-dimensional data is of primary interest for many branches of applied sciences. It is typically achieved by applying dimensionality reduction techniques, which, while preserving local features, often miss the global structure of the dataset. Clustering techniques are another class of methods operating in the ambient space, grouping together similar points. However, unlike dimensionality reduction techniques, they do not provide information about the organization of the data.

**Results:**

Leveraging ideas from Topological Data Analysis, we introduce *ClusterGraph*, an additional layer on the output of any clustering algorithm that represents clusters as vertices and inter-cluster relationships as weighted edges. This structure captures the large-scale organisation of a dataset without forcing it into a two-dimensional Euclidean embedding. The method comes with a built-in quality criterion, metric distortion, which quantifies how faithfully the graph reflects the intrinsic geometry of the data and provides a principled basis for pruning, model selection, and parameter tuning. ClusterGraph, possibly with an appropriate structure-preserving simplification, can be visualized and used in synergy with state-of-the-art exploratory data analysis techniques.

**Conclusion:**

ClusterGraph is complementary to methods such as UMAP, t-SNE, and PHATE: by encoding inter-cluster geometry directly in graph form, it serves not only as a visualisation tool but also as a diagnostic layer that can validate, refine, or question conclusions drawn from low-dimensional embeddings.

## Introduction

High-throughput experiments are becoming extremely common in applied sciences. Now more than ever, large high-dimensional datasets are generated in almost every laboratory, calling for an automated and reliable way to extract new knowledge from them. Let us fix a dataset $X,$ usually embedded in a high-dimensional space. Standard dimension reduction techniques, including principal component analysis (PCA) [1], t-SNE [2], umap [3], and phate [4], aim to find a low-dimensional embedding of $X$ so that points that are close in $X,$ are also close in the embedding. However, preservation of the global organization of $X$in general, and information about distances of distant points in particular, is a challenge for these methods.

Clustering techniques [5, 6], on the other hand, based on a fixed similarity measure, provide a partition of the input dataset $X.$ However, clustering itself does not provide information about either intra- or inter-cluster organization of points and is therefore not used to assess the global structure of the data.

In computational biology, both dimensionality reduction and clustering have become essential tools for the analysis of omics data. On the visualization side, dedicated tools have been developed to cope with the growing size and intricacy of single-cell data, either by providing interactive exploration of hierarchical cell populations [7] or by extending classical dimensionality reduction methods to integrate spatial and molecular information jointly [8]. On the clustering side, efforts have focused both on automating method selection for transcriptomic data [9] and on recovering biologically meaningful hierarchical structure that flat partitions fail to capture [10, 11].

The aim of this work is to bridge these 2 approaches by enriching the output of a clustering algorithm with additional information on the data’s global organization.

The first contribution of this paper is the construction of a *ClusterGraph*: a graph-based structure on top of a partition $\mathcal {C}(X)$ of the data obtained from a clustering algorithm $\mathcal {C}$ applied to $X.$ In the ClusterGraph $G = (V,E),$ each vertex corresponds to a single cluster from $\mathcal {C}(X).$ Two vertices $u,v \in V$ are connected by an edge whose length corresponds to the distance between their respective clusters in $\mathcal {C}(X).$ For the purpose of this construction, a number of inter-cluster distances defined in the ambient space are used.

The ClusterGraph has a number of advantages compared to alternative dimension reduction methods. One of them is based on the fact that the distances, computed in the ambient space, are represented by labels on edges and not subject to distortions made by standard dimension reduction techniques that force the projected data points to be embedded in a Euclidean space. This allows us to visualize the *global* distances in the dataset. This is important as many datasets cannot be embedded into low-dimensional Euclidean spaces without perturbing the distances between points.

**Figure 1:**
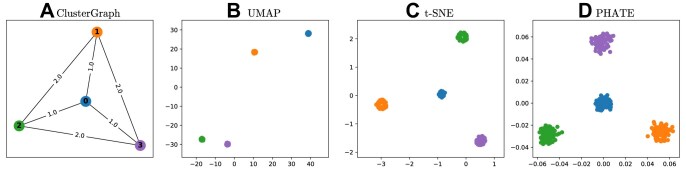
The dataset consists of 4 clusters 0, 1, 2, and 3, as described in the text, so that elements of cluster 0 are at distance one from elements from the remaining clusters, and the mutual distances between elements of clusters 1, 2, and 3 are equal to 2. Such a dataset cannot be embedded, with distances preserved, into any Euclidean space. In this case, UMAP (panel B) fails to capture the global layout, while t-SNE (panel C) and PHATE (panel D) do. However, the coordinate systems of t-SNE and PHATE are drastically different. In both cases, as a result of the embedding into the Euclidean plane, the ratio of the distances $d(1,2) / d(0,1)$ is roughly $\sqrt{3}$ instead of the original 2, the same is true for the other clusters. This is the optimal embedding that can be achieved when points are projected to Euclidean space. However, in the case of ClusterGraph (panel A), the distances are encoded as labels on the graph edges, and therefore we are not restricted by any Euclidean space.

As an example of such a situation, consider a collection of points in 4 clusters: 0, 1, 2, and 3. Points in each cluster are infinitesimally close. The distance between cluster 0 and clusters 1, 2, and 3 is 1, while the distance between clusters 1, 2, and 3 is 2. It is well known [12, 13] that such a graph cannot be isometrically embedded to any Euclidean space $\mathbb {R}^n$ for any $n$. As a consequence, all dimensionality reduction techniques will distort the distances between clusters, as can be observed in Fig. 1. The ClusterGraph, on the contrary, provides the correct graph even in this case.

The second contribution is a method to assess the quality of the ClusterGraph $G$. Working under the assumption that $X$ is sampled from a manifold equipped with an intrinsic distance, a *metric distortion* between the intrinsic distances on $X$ and the distance induced by $G$ on $X$ is used to assess the quality of $G$. The distance between points $x,y \in X$ induced by $G$ is the length of the shortest path in $G$ between vertices representing the clusters containing them.

The logarithm of the ratio between the intrinsic and the ClusterGraph distance is used as a quality measure: the smaller its value, the better the quality of the ClusterGraph representation.

**Figure 2:**
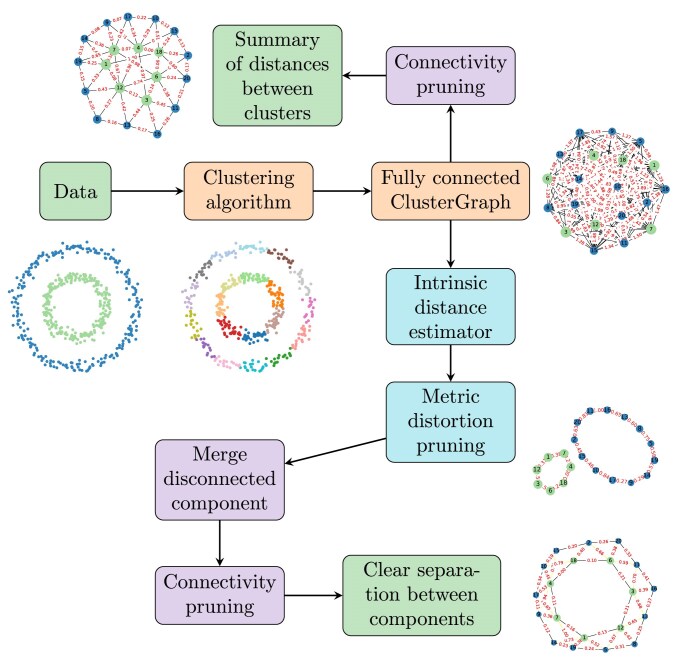
ClusterGraph pipeline with the 2 possible pruning strategies. Details on the example dataset used to generate the figures can be found in the “Concentric circles” section.

This procedure will be used to obtain a *pruned ClusterGraph* that better approximates the intrinsic structure of the data. For this purpose, a number of *edge pruning* algorithms are proposed, aiming to remove some edges while maintaining the global structure of the data. A schematic of the whole ClusterGraph pipeline is depicted in Fig. 2.

A Python implementation of the creation of the ClusterGraph data structure, as well as the pruning algorithms and interactive visualization utilities, is available at GitHub repository.

## Methods

### ClusterGraph

Let $X$ be a dataset equipped with a metric $d_X : X \times X \rightarrow \mathbb {R}_{\ge 0}.$ Take $\mathcal {C}$ to be an arbitrary hard or soft clustering algorithm. Let $\mathcal {C}(X)$ be the partition or, in a more general case, a division of $X$ obtained from the clustering algorithm $\mathcal {C}.$

A collection of sets $\lbrace C_i \rbrace$ is a *partition* of $X$ if for every $C_i \ne C_j \in \mathcal {C}(X),$  $C_i \cap C_j = \emptyset$ and $\bigcup _{C_i \in \mathcal {C}(X)} C_{i} = X.$ Partitions of $X$ are typically obtained from hard clustering algorithms [5]. In a more general case, we can drop the empty intersection condition turning $\mathcal {C}(X)$ into a *division* of $X.$ Divisions may be obtained using soft or fuzzy clustering methods [14]. They can also be obtained as by-products of Topological Data Analysis techniques like Mapper [15] or Ball Mapper [16].

In the next step, we extend $d_X$ to a distance or similarity measure ${ d_\mathcal {C} : \mathcal {C}(X) \times \mathcal {C}(X) \rightarrow \mathbb {R}_{\ge 0} }$ defined on elements of $\mathcal {C}(X),$ as detailed in the “Distances between clusters” section. The *ClusterGraph* of a partition $\mathcal {C}(X)$ is a fully connected graph whose vertices are elements of $\mathcal {C}(X),$ and edges are weighted by the distance $d_\mathcal {C}$ between the elements of $\mathcal {C}(X).$

ClusterGraph is intended to serve as a tool for data visualization and compression. Compression is achieved by collapsing points within the same cluster into a vertex of the ClusterGraph. This step is motivated by an assumption that points within the same cluster are in close proximity, hence share multiple characteristics and can be represented by a single vertex. We expect the ClusterGraph to have much fewer vertices compared to the original number of data points. The visualization aspect is accomplished when the layout of the ClusterGraph resembles, to some extent, the layout of the input point cloud. However, this is unlikely to be the case for a fully connected graph. Therefore, in the “Metric distortion” section, we propose a *metric distortion*-based criterion and techniques for removing certain edges of the ClusterGraph so that the metrics on the graph and on the initial point cloud become comparable. Different strategies for edge removal from the ClusterGraph are discussed in the “ClusterGraph pruning” section. This process yields a *pruned* ClusterGraph, which we use for visualization purposes.

#### Distances between clusters

In this section, for a dataset $X$ equipped with a similarity measure $d_X$ and a partition $\mathcal {C}(X)$, we present a number of similarity measures $d_\mathcal {C} : \mathcal {C}(X) \times \mathcal {C}(X) \rightarrow \mathbb {R}_{\ge 0}$. The choice of the optimal one is application-dependent, very much like the clustering algorithm $\mathcal {C}$, and should therefore be selected and optimized by the user. Given 2 clusters $C_i$ and $C_j$, possible options include

1. Maximum, minimum, or average distance between points
  \begin{eqnarray*}
\min (C_i, C_j) &=& \min \limits _{x \in C_i , y \in C_j } d_X(x,y), \\\max (C_i, C_j) &=& \max \limits _{x \in C_i , y \in C_j } d_X(x,y),\\{\mathrm{avg}}(C_i , C_j) &=& {\sum \limits _{x \in C_i} \sum \limits _{y \in C_j} d_X(x, y)} / {(|C_i| |C_j|)} .
\end{eqnarray*}
2. Hausdorff distance
  \begin{eqnarray*}
d_{H}(C_i,C_j)=\max \left\lbrace \, \sup _{x\in C_i}d(x,C_j),\, \sup _{y\in C_j}d(C_i,y)\, \right\rbrace ,
\end{eqnarray*}
  where $d(a, B) = \inf \limits _{b \in B} d(a,b)$.
3. Earth mover’s (a.k.a. Wasserstein) distance [17, 18], which utilizes ideas from probability theory and optimal transport
  \begin{eqnarray*}
W_p(C_i, C_j) = \inf _{\eta :C_i \rightarrow C_j} \left(\sum _{x \in C_i} d_X(x, \eta (x))^p \right)^\frac{1}{p},
\end{eqnarray*}
  where $\eta$ is a matching between points of $C_i$ and $C_j$ and $1 \le p < \infty$. If the 2 clusters have different size, the matching is computed in a weighted way, i.e., each point in $C_i$ is assigned a weight of $1/|C_i|$ such that each cluster has a total mass of 1.

#### Stability

Let us consider a dataset $X$ and 2 partitions of it $\mathcal {C}(X)$, $\mathcal {D}(X)$ obtained via some clustering algorithms. We are interested in quantifying how different these 2 partitions can be. In order to do so, we introduce the following concept.

Definition 1 **(Image of a cluster).**

Let $X$ be a dataset and $\mathcal {C}(X)$ and $\mathcal {D}(X)$ 2 partitions of it. The *image* of a cluster $C_i \in \mathcal {C}(X)$ in $\mathcal {D}(X)$ is the union of all clusters of $\mathcal {D}(X)$ that contain some points of $C_i$, namely $\operatorname{im}_{{\mathcal D}(X)} (C_i) = \lbrace \bigcup D_j \in {\mathcal D}(X) \; | \; C_i \cap D_j \ne \emptyset \rbrace$.

This idea of mapping the points covered by one cluster in a given partition to the clusters in a second partition is inspired by an analogous technique for mapper graphs, *MappingMappers*, described in [19].

Let us define the *diameter* of a collection of points as the greatest distance between any pair of points. We can then state the following bound.

Proposition 1 **(Clustering stability).**

Let $X$ be a dataset and $\mathcal {C}(X)$, $\mathcal {D}(X)$ be 2 partitions of it such that the diameter of each set in $\mathcal {C}(X)$ and $\mathcal {D}(X)$ is at most $\delta$. Then, for any cluster $C_i \in \mathcal {C}(X)$, its image in $\mathcal {D}(X)$ has diameter at most $3\delta$.

Proof . Let $d_1, d_2 \in X$ be the 2 points whose distance realizes the diameter of $\operatorname{im}(C_i)$. By definition of image, there exists at least one point $c_1 \in C_i$ which lies in the same cluster of $\mathcal {D}(X)$ as $d_1$, and similarly there exists at least one $c_2$ for $d_2$. Therefore, we have

\begin{eqnarray*}
d_X(d_1, d_2) \le d_X(d_1, c_1) + d_X(c_1, c_2) + d_X(c_2, d_2) \le 3\delta.
\end{eqnarray*}

Using a summary statistic of the distance between points as the distance between clusters (option 1 in the “Distances between clusters” section) allows us to derive a similar bound for ClusterGraphs built on top of $\mathcal {C}(X)$ and $\mathcal {D}(X)$.

Definition 2 **(Image of a ClusterGraph vertex).**

Let $X$ be a dataset, $\mathcal {C}(X)$ and $\mathcal {D}(X)$ 2 partitions of it and $G_{\mathcal {C}(X)}$, $G_{\mathcal {D}(X)}$ the ClusterGraphs obtained from $\mathcal {C}(X)$ and $\mathcal {D}(X)$. The *image* of a vertex $i \in G_{\mathcal {C}(X)}$ (corresponding to cluster $C_i \in G_{\mathcal {C}(X)}$) in $G_{\mathcal {D}(X)}$ is the collection of all vertices of $G_{\mathcal {D}(X)}$ that correspond to clusters in $G_{\mathcal {D}(X)}$ containing some points of $C_i$.

We define the *diameter* of a weighted graph as the greatest distance between any pair of vertices.

Proposition 2 **(ClusterGraph stability).**

Let $X$ be a dataset, $\mathcal {C}(X)$ and $\mathcal {D}(X)$ 2 partitions of it and $G_{\mathcal {C}(X)}$ and $G_{\mathcal {D}(X)}$ the ClusterGraphs obtained from them. Assume that the diameter of each set in $\mathcal {C}(X)$ and $\mathcal {D}(X)$ is at most $\delta$. Then the image of each vertex $u \in G_{\mathcal {C}(X)}$ in $G_{\mathcal {D}(X)}$ is a clique of diameter at most $3\delta$ for the maximum and average distance, and $\delta$ for the minimum.

Proof . Let $u$ be a vertex in $G_{\mathcal {C}(X)}$ and $\operatorname{im}(u)$ its image in $G_{\mathcal {D}(X)}$.

Recall that $\operatorname{im}(u)$ is a subset of the complete graph $G_{\mathcal {D}(X)}$; therefore, it is a clique. Let $D_i$ and $D_j$ be the clusters in $\operatorname{im}(u)$ whose distance realizes the diameter of $\operatorname{im}(u)$, i.e., they correspond to the 2 vertices in the clique that are furthest apart.

Let us start with the maximum distance case. In particular, let $d_1 \in D_i$ and $d_2 \in D_j$ be the 2 data points realizing the maximum distance between $D_i$ and $D_j$, and therefore $d(d_1, d_2) = \operatorname{im}(u)$. Let $C_u$ be the cluster in $\mathcal {C}(X)$ corresponding to vertex $u \in G_{\mathcal {G}(X)}$. By definition of the image of $u$, there are at least 2 points $c_1, c_2 \in C_u$ that lie in the same clusters of $\mathcal {D}(X)$ as $d_1$ and $d_2$, respectively. We can then proceed in a similar fashion to the proof of Proposition 1, namely we have

\begin{eqnarray*}
{\mathrm{diam}}({\mathrm{im}}(u)) &=& \max (D_i, D_j) = d_X(d_1, d_2) \le d_X(d_1, c_1) \\&&+ d_X(c_1, c_2) + d_X(c_2, d_2) \le 3\delta .
\end{eqnarray*}

The same bound holds for the average distance since $\operatorname{avg}(D_i , D_j) \le \max (D_i, D_j)$.

For the minimum distance case, it is sufficient to notice that $d_X(c_1, c_2) \le \delta$ because they both belong to the same cluster $C_i$ whose diameter is bounded by $\delta$. Hence, we have

\begin{eqnarray*}
{\mathrm{diam}}({\mathrm{im}}(u)) = \min (D_i, D_j) \le d_X(c_1, c_2) \le \delta .
\end{eqnarray*}

### Metric distortion

Given a dataset $X$, different choices of the clustering algorithm $\mathcal {C}$, as well as the metrics $d_X$ and $d_\mathcal {C}$, can lead to significantly different ClusterGraphs. The aim of this section is to introduce a score to assess the quality of a given ClusterGraph $G$ by comparing it to the underlying geometric structure of the dataset $X$.

To this end, let us assume that the point cloud $X$ is sampled from a compact and connected manifold $\mathcal {M}$ equipped with an *intrinsic distance*  $d_{\mathcal {M}}$. Informally, the intrinsic distance between 2 points $x,y \in \mathcal {M}$ is defined as the infimum of the length of a curve $\gamma \subset \mathcal {M}$ joining $x$ and $y$; this is also known as *geodesic* distance.

In most applications, the underlying manifold is not known. Consequently, the intrinsic distance needs to be estimated from the point cloud. This is a well-studied problem in computational geometry and computer graphics, and multiple methods have been proposed [20–23].

Below, we follow the approach of [20, 24] using the shortest path in the $k$-nearest neighbour graph as estimator. Note that any other estimator of intrinsic distance can also be used in the proposed construction.

Let $G_{k\mathrm{ nn}}(X)$ be the $k$-nearest neighbour graph on $X$ constructed as follows: each point of $X$ corresponds to a vertex of $G_{k\mathrm{ nn}}(X)$; it is connected to its $k$-nearest neighbours (in the chosen distance $d_X$, typically Euclidean), with $k$ being a parameter of the method. Weights corresponding to the distance between endpoints are assigned to the edges of $G_{k\mathrm{ nn}}(X)$. We define a distance $d_X^k$ on $G_{k\mathrm{ nn}}(X)$, estimating the intrinsic distance on $X$, as


(1) \begin{eqnarray*}
d_X^k(x,y) & =& \text{the length of the shortest path between}\, \\&& x\, \mathrm{and}\, y\, \mathrm{in}\, G_{k\mathrm{ nn}}(X).
\end{eqnarray*}

Remark 1. It may happen that $G_{k\mathrm{ nn}}(X)$ is not connected. There are 2 possible reasons for this. In the first case, points are indeed sampled from a compact and connected manifold, but the parameter $k$ is too low. This can be easily solved by increasing $k$. In the second case, the underlying manifold is not connected. This will result in the $k$-nn graph being disconnected even for very high values of $k$, especially if many points are sampled. In this case, we will treat each connected component separately, splitting the input dataset $X$ (and the output of the clustering algorithm) into disjoint sets, each one corresponding to a different connected component and analyse each of them separately. (By performing the construction in the “ClusterGraph” section, we obtain a ClusterGraph for each connected component, each of them being a fully connected graph. In graph theory, such a disjoint union of complete graphs is sometimes called a “cluster graph.” This unexpected but pleasing agreement in nomenclature motivates our choice of referring to our construction in camel case, to avoid confusion.) For the rest of the section, we therefore assume, without lack of generality, that the $k$-nn graph is fully connected. We discuss how to investigate the relations between different connected components in the “Connectivity-based pruning” section

Remark 2. Whenever an estimator is used, it is natural to ask how good such an estimator is. The choice of a $k$-nn graph as an estimator of the geodesic distance is motivated by the following theorem by Bernstein, Vin de Silva, Langford, and Tenenbaum.

Theorem 3 **(Theorem A in [24]).**

Let $\mathcal {M}$ be a compact submanifold of $\mathbb {R}^n$, $X$ a finite set of data points in $\mathcal {M}$ and $G$ a graph on $X$ (e.g., a $k$-nn graph). Then the inequalities

\begin{eqnarray*}
(1-\lambda _1) d_\mathcal {M}(x, y) \le d_G(x,y) \le (1+\lambda _2) d_\mathcal {M}(x, y)
\end{eqnarray*}

are valid for all $x,y$ in $X$, where $\lambda _1, \lambda _2 < 1$ are 2 positive real numbers that depend on $G$, $\mathcal {M}$ and some technical assumptions on the density of $X$.

For each point $x \in X$, we denote by $C_x$ the cluster in $\mathcal {C}(X)$ that contains $x$. We can then use the distance between clusters described in the “Distances between clusters” section to define a distance between points $d_{CG}$ in the ClusterGraph $G$ as follows:


(2) \begin{eqnarray*}
d_{CG}(x,y) & =& \text{the length of the shortest path between}\\&& C_x\, \mathrm{and}\, C_y\, \mathrm{in}\, G.
\end{eqnarray*}

Recall that $G$ is fully connected; one might wonder why the length of the shortest path between 2 vertices is used instead of the weight of the edge connecting them given by $d_\mathcal {C}$. First, the triangle inequality might not hold for $d_\mathcal {C}$. Second, we want the definition of $d_{CG}$ to also hold in the case of a pruned ClusterGraph, which we will discuss in the “ClusterGraph pruning” section.

This notion of $d_{CG}$ is well defined only when $\mathcal {C}(X)$ is a partition. In the more general case of a division, when a point can belong to more than one cluster, we take $d_{CG}(x,y)$ to be the length of the shortest path between any cluster containing $x$ and any cluster containing $y$.

Consider the ClusterGraph $G = (V, E)$ and let us fix 2 vertices, $i,j \in V$, corresponding to 2 clusters $C_i$ and $C_j$. For a pair of points $x \in C_i$ and $y \in C_j$, $x \ne y$, we compute


(3) \begin{eqnarray*}
\delta (x,y) = \left\vert \log \left(\frac{ d_{CG}(x,y) }{ d_X^k(x,y) }\right) \right\vert .
\end{eqnarray*}

The use of the absolute value of the logarithm ensures that a multiplicative scaling by a factor $\lambda = \tfrac{ d_{CG}(x,y) }{ d_X^k(x,y)}$ results in the same metric distortion as a $1/\lambda$ scaling, i.e., $\vert \log (x/y)\vert = \vert \log (y/x)\vert$ for every $x,y \in \mathbb {R}_{> 0}$.

By averaging this quantity over all possible pairs of points $x \in C_i$ and $y \in C_j$, we obtain the metric distortion between clusters $C_i$ and $C_j$.


(4) \begin{eqnarray*}
\delta _{\lbrace i,j\rbrace } = \frac{1}{|C_i| |C_j|} \sum \limits _{ (x,y) \in (C_i,C_j) }\delta (x,y).
\end{eqnarray*}

This score assesses how much the intrinsic distance between points on $X$ differs from their corresponding distance in the ClusterGraph.

The global metric distortion of the ClusterGraph can be obtained by averaging the score for each pair of vertices defined in Equation (4). However, because clusters can have different sizes, we consider a weighted average. The weight of the pair $\lbrace i,j\rbrace$ corresponding to clusters $C_i$ and $C_j$ is defined as


(5) \begin{eqnarray*}
w_{\lbrace i,j\rbrace } = \frac{ |C_i \cup C_j|}{ (n-1) |X| } ,
\end{eqnarray*}

where $n$ denotes the number of clusters in $\mathcal {C}(X)$ (or equivalently, $n = |V|$, the number of vertices in the ClusterGraph). The rationale behind this averaging is to give more importance to interactions between large clusters.

The global metric distortion for a ClusterGraph (with respect to the $k$-nearest neighbours graph) can then be defined as


(6) \begin{eqnarray*}
\Delta _k(G) = \frac{2}{n(n-1)} \sum _{ \lbrace i,j\rbrace \in V } w_{\lbrace i,j\rbrace } \delta _{\lbrace i,j\rbrace } .
\end{eqnarray*}

This quantity is a non-negative real number indicating how well the given ClusterGraph respects the intrinsic metric structure of the data. It allows us to compare the quality of ClusterGraphs having an equal or very similar number of vertices. Comparison of metric distortions of ClusterGraphs having vastly different numbers of nodes should not be performed.

In the next sections, we will use each edge’s distortion as well as the global distortion to prune the ClusterGraph, with the goal of removing the edges that do not reflect the underlying structure of the data. In practice, computing the metric distortion can be computationally intensive, particularly during pruning. In our current implementation, the following approximation is used instead.

We first compute the average intrinsic distance between clusters:


(7) \begin{eqnarray*}
d_{CG}^{\mathrm{geod}}(C_i, C_j) = \frac{1}{|C_i||C_j|} \sum _{x \in C_i} \sum _{y \in C_j} d_X^k(x, y).
\end{eqnarray*}

Comparing this quantity with the ClusterGraph edge weight yields the approximate metric distortion:


(8) \begin{eqnarray*}
\widetilde{\Delta _k}(G) = \frac{2}{n(n-1)} \sum _{\lbrace i,j\rbrace \in V} w_{\lbrace i,j\rbrace } \left|\log \!\left(\frac{d_{CG}(C_i, C_j)}{d_{CG}^{\mathrm{geod}}(C_i, C_j)}\right)\right|,
\end{eqnarray*}

where $d_{CG}(C_i, C_j)$ denotes the shortest path between clusters $C_i$ and $C_j$ in the ClusterGraph.

This approximation is particularly useful when the metric distortion must be evaluated multiple times, such as during the pruning procedure.

Remark 3. The ratio between 2 distances in our definition of the distortion (Equation (3)) might be reminiscent of the *stretch factor* or *distortion* of an embedding $f$. For 2 given points $x$ and $y$ in a metric space, the stretch factor is defined as ${d(f(x) ,f(y))}/{d(x,y)}$. For example, consider a set of points in $\mathbb {R}^d$ and a connected graph having those points as vertex set. Each edge in the graph has a weight corresponding to the Euclidean distance between its endpoints. The stretch factor for 2 given points is the ratio of the length of the shortest path between them in the graph to their Euclidean distance. The stretch factor of the graph is the maximum stretch factor over any pair of points. Graphs with stretch factor at most $t$ are called *$t$-spanners* [25].

It is important to point out the differences between this widely studied topic in graph theory and our approach. First, we are not dealing with an embedding as the map that sends each data point to its cluster is highly non-injective. Moreover, the stretch factor of a graph defined in the paragraph above is always greater than or equal to 1. In our setting, the ratio between the ClusterGraph distance and the intrinsic one (Equation (3)) might be less than 1; this is exactly the case of a “shortcut” edge in the ClusterGraph.

### ClusterGraph pruning

The ClusterGraph is, by definition, a fully connected graph. Consequently, it may contain edges connecting regions of the dataset that are not close in the manifold $\mathcal {M}$ from which the data points of $X$ are sampled. We will refer to these edges as “shortcuts,” as they are shorter than the true geodesic distance between the corresponding points in the manifold and therefore are not representative of the underlying manifold structure. The removal of such edges will make the ClusterGraph more similar, in the sense of metric distortion, to $X$. Moreover, the ClusterGraph may also contain edges whose removal does not considerably change the metric structure of the graph. In this section, we introduce 3 approaches to pruning edges of a given ClusterGraph, and by doing so, of increasing the quality of the obtained representation.

#### Threshold pruning

If the triangle inequality holds for the distance between clusters $d_\mathcal {C}$, the length of the shortest path between each pair of vertices in the ClusterGraph (Equation (2)) is exactly the length of the edge connecting them. It then makes sense to assign to each edge in the ClusterGraph the metric distortion for its 2 corresponding clusters, as defined in Equation (4).

The ClusterGraph can then be naively pruned by removing all edges having metric distortion greater than a threshold $\alpha > 0$.

#### Iterative greedy pruning

Consider the ClusterGraph $G =(V,E)$. Let $\Delta _k(G)$ be its metric distortion as in Equation (6). Denote by $G_{\hat{e}}$ the ClusterGraph obtained by removing edge $e$ from $G$, namely $G_{\hat{e}} = (V, E \setminus \lbrace e\rbrace )$.

We can then perform the following iterative greedy pruning procedure. Remove edge $e$ if both conditions hold:

1.
$\Delta _k(G_{\hat{e}}) \le \Delta _k(G)$ ,
2.
$\Delta _k(G_{\hat{e}}) \le \Delta _k(G_{\hat{e}^{\prime }})$ for any other $e^{\prime } \in E$.

Then update $E$ to $E \setminus \lbrace e\rbrace$. The process may be repeated a fixed number of times, or until no such edge $e$ can be found. Note that the length of the shortest path between 2 vertices defined in Equation (2) will be infinite if the ClusterGraph becomes disconnected, thereby leading to an infinite value of the metric distortion. Therefore condition (1) ensures that the pruning procedure will never produce new connected components in the ClusterGraph.

#### Connectivity-based pruning

The first 2 pruning techniques presented in “Threshold pruning” and “Iterative greedy pruning” sections focus on the removal of the edges with high metric distortion or, informally speaking, the removal of all the “shortcuts” with respect to the structure of $X$. It might happen that after this pruning the obtained ClusterGraph still has a complicated structure which may render its visualization and interpretation challenging.

In what follows we adopt the *connectivity-based* approach by Zhou, Mahler, and Toivonen [26] to the ClusterGraph pruning. A *path*  $P$ in $G=(V, E)$ is a set of edges $P = \lbrace \lbrace i_1, i_2\rbrace , \lbrace i_2, i_3 \rbrace , \dots , \lbrace i_{k-1}, i_k \rbrace \rbrace \in E$. A *path quality function*  $q(P) \rightarrow \mathbb {R}^+$ is defined by taking the sum of the inverse of the path length, calculated using the distance between clusters $d_\mathcal {C}$


(9) \begin{eqnarray*}
q(P) = \sum _{ \lbrace i,j\rbrace \in P} \frac{1}{d_\mathcal {C}(C_i, C_j)} .
\end{eqnarray*}

The *connectivity* between 2 vertices $i,j$ in $G=(V,E)$ is the quality of the best path between them


(10) \begin{eqnarray*}
\mathrm{conn}(i,j;E) = \left\lbrace \begin{array}{@{}l@{\quad }l@{}}\max _{P \in \mathcal {P}(i, j)} q(P) &\text{if } \mathcal {P}(i, j) \ne \emptyset \\-\infty & \mathrm{otherwise} \end{array}\right.
\end{eqnarray*}

where we denote by $\mathcal {P}(i, j)$ the set of all possible paths between vertices $i$ and $j$. The *connectivity of a ClusterGraph* is the average connectivity over all pairs of vertices


(11) \begin{eqnarray*}
\mathrm{conn}(V,E) = \frac{2}{n(n-1)} \sum _{i,j \in V, i \ne j}\mathrm{conn}(i,j;E),
\end{eqnarray*}

where $n$ is the number of vertices in the ClusterGraph. Note that the connectivity will be $-\infty$ if $G$ is disconnected. In that case each connected component should be analysed separately.

Let us now consider the ClusterGraph with one edge removed $G_{\hat{e}} = (V, E \setminus \lbrace e\rbrace )$. It is straightforward to see that $\operatorname{conn}(V, E \setminus \lbrace e\rbrace ) \le \operatorname{conn}(V,E)$. In particular ${\operatorname{conn}(V, E \setminus \lbrace e\rbrace ) = \operatorname{conn}(V,E)}$ if and only if $e$ does not belong to any of the best paths between any pairs of vertices. Moreover, $\operatorname{conn}(V, E \setminus \lbrace e\rbrace ) = -\infty$ if the removal of $e$ disconnects the graph. We can then define the *ratio of connectivity kept* after removing an edge or, more generally, after removing a subset of edges $E_R \subset E$


(12) \begin{eqnarray*}
\mathit {rk}(V, E, E_R) = \frac{\mathrm{conn}(V, E \setminus E_R)}{\mathrm{conn}(V, E)}.
\end{eqnarray*}

Pruning can then be executed in a greedy iterative fashion (see Alg. 2 BF in [26]) by selecting, at each iteration, the edge whose removal will result in smallest decrease of connectivity, i.e., the largest $\mathit {rk}$ value.

### Merging

As discussed in Remark 1, it may happen that the underlying manifold from which the data are sampled is disconnected. In that case, the resulting ClusterGraph will have more than one connected component and each of them will be pruned separately. In order to capture the global layout of the data, including the disconnected components of the manifold, we may merge different components by adding a collection of special edges between each vertex $v$ and its $k$-nearest neighbours not belonging to the same connected component as $v$. Subsequently, the connectivity-based pruning procedure can be applied to the newly added edges.

### ClusterGraph scalability

At scale, computing the ClusterGraph, evaluating the metric distortion, and performing the iterative pruning can become computationally demanding. A detailed complexity analysis is provided in Supplementary_Appendix.pdf. Several strategies can keep the ClusterGraph tractable on large datasets. Exact $k$-NN graph construction can be replaced with an approximate method such as HNSW [27], reducing the complexity from $O(|X|^2 d)$ to $O(|X| \log |X|)$. Furthermore, performing PCA prior to graph construction reduces the cost of each distance computation. Similarly, using cluster centroids for all pairwise point distances reduces the inter-cluster cost from $O(|X|^2)$ to $O(|X|d + n^2 d)$. Finally, inter-cluster distance computations and single-source Dijkstra runs are mutually independent and can be executed in parallel across available workers. Beyond these, we introduce 2 more strategies that significantly extend the scalability of ClusterGraph to larger datasets.

#### $k^{\prime }$ -NN ClusterGraph approximation

In practice, pruning preserves only connections to nearest neighbour clusters when the data exhibit geometric locality. Consequently, assessing metric distortion on long-range edges is often of limited value, as these edges are likely to be discarded anyway. This naturally leads to the following idea. Rather than pruning the fully connected ClusterGraph iteratively, one can construct directly a $k^{\prime }$-nearest neighbour graph on the $n$ clusters, retaining for each cluster only the edges to its $k^{\prime }$ nearest neighbours. This yields a ClusterGraph that approximates the geometric structure of the dataset at a cost of $O(n^2)$, which is negligible when $n \ll |X|.$

The metric distortion (Equation 6) can then be evaluated once on this $k^{\prime }$-NN ClusterGraph, providing a fast way to assess how well the graph captures the intrinsic geometry of the data for a given value of $k^{\prime }$. Additionally, the standard pruning procedure described in this paper can be performed for such a $k^{\prime }$-NN ClusterGraph at much lower cost.

#### Scalable landmark-based approximation

Most strategies presented above reduce constant factors or per-iteration costs but leave the asymptotic complexity unchanged, as the $O(|X|^2)$ bottleneck arising from the construction of the intrinsic distance matrix $d_X^k$ persists regardless. We now propose a landmark-based approximation that addresses this bottleneck directly by reducing the effective dataset size from $|X|$ to $|X^{\prime }| \ll |X|$. Rather than operating on all $|X|$ points, the pipeline is applied to a small set of representative points, or *landmarks*, selected to preserve both the global geometric structure of the dataset and the local intra-cluster structure. This yields a genuine asymptotic improvement across all steps whose complexity depends on $|X|$.

Given a dataset $X$ of $|X|$ points partitioned into $n$ clusters $\mathcal {C} = \lbrace C_1, \ldots , C_n\rbrace$, we construct a reduced dataset $X^{\prime } \subset X$ as the union of 2 complementary sets of landmarks:


(13) \begin{eqnarray*}
X^{\prime } = L_m \cup \bigcup _{i=1}^n L_i, \qquad |X^{\prime }| \approx m + \sum _{i=1}^n m_i,
\end{eqnarray*}

where

-
$L_m$ is a set of $m = \lfloor \sqrt{|X|}\rfloor$ global landmarks selected from $X$ using the MaxMin algorithm [28], which iteratively selects the point furthest from all previously selected landmarks:
  (14) \begin{eqnarray*}
l_t = \arg \max _{x \in X} \min _{l \in L_{t-1}} d(x, l), \quad t = 1, \ldots , m.
\end{eqnarray*}
  This guarantees uniform geometric coverage of the full dataset, capturing inter-cluster structure and boundary regions. The choice $m = \sqrt{|X|}$ is consistent with the Landmark Isomap literature [28], which establishes that $O(\sqrt{|X|})$ landmarks suffice for a good geodesic approximation.
-
$L_i \subset C_i$ is a set of intra-cluster landmarks of size $m_i = \lfloor \sqrt{n_i}\rfloor$, where $n_i = |C_i|$ is the number of points in cluster $C_i$, and $\lfloor \cdot \rfloor$ denotes the floor function. The landmarks are selected using MaxMin applied locally within each cluster $C_i$. This mirrors the global strategy at the cluster level, ensuring that each cluster is represented by a number of landmarks proportional to the square root of its size, guaranteeing uniform geometric coverage regardless of cluster size or shape.

Selecting landmarks randomly at both global and local levels also yields good results in practice [28]. The 2 sets serve complementary roles: $L_m$ captures global inter-cluster geometry, while $\lbrace L_i\rbrace$ captures local intra-cluster structure. The total size of the reduced dataset is


(15) \begin{eqnarray*}
|X^{\prime }| = m + \sum _{i=1}^n m_i = \sqrt{|X|} + \sum _{i=1}^n \sqrt{n_i}.
\end{eqnarray*}

By the Cauchy–Schwarz inequality, $\sum _{i=1}^n \sqrt{n_i} \le \sqrt{n \sum _{i=1}^n n_i} = \sqrt{n|X|}$, and therefore


(16) \begin{eqnarray*}
|X^{\prime }| = \sqrt{|X|} + \sum _{i=1}^n \sqrt{n_i} \le \sqrt{|X|}(1 + \sqrt{n}) = O(\sqrt{n|X|}).
\end{eqnarray*}

All subsequent steps of the pipeline are performed on $X^{\prime }$ instead of $X$.

## Results

### Concentric circles

**Figure 3:**
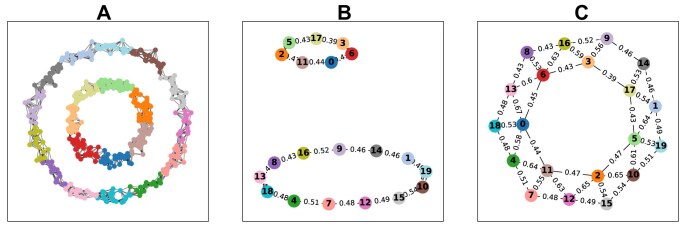
ClusterGraph built on the output of *k*-means for 500 points sampled from 2 concentric circles of diameter 1 and 2. The 20 clusters are depicted in (A), on top of the 10-nn graph. The 2 metric distortion-pruned components are depicted in (B) and subsequently merged and connectivity pruned (C). Vertex colours are inherited from panel (A).

To showcase the whole ClusterGraph pipeline (Fig. 2), we consider 500 points sampled from 2 concentric circles in the plane, depicted in Fig. 3. Clusters are computed using $k$-means with 20 centroids, and a ClusterGraph is built using the average Euclidean distance between points. The 10-nearest neighbours graph is used to estimate the intrinsic distance between data points. The iterative metric distortion pruning procedure is applied, and the pruned ClusterGraph is shown in Fig. 3B. Note that the pruned ClusterGraph has 2 connected components, as a consequence of the 10-nearest neighbours graph having 2 connected components. Finally, the 2 components are merged by adding an edge between each vertex and its 3-nearest neighbours in the other connected component. We then prune 20 of these newly introduced edges using the connectivity-based approach. The resulting ClusterGraph is depicted in Fig. 3C.

### Mice protein expression

We analyse the expression levels of 77 proteins obtained from 38 normal genotype control mice and from 34 of their trisomic littermates, both with and without treatment with the drug memantine and with and without the stimulation to learn [29]. The original dataset (more details in “Data Availability” section) contains 15 measurements of each protein per sample, for a total of 1,080 data points. Control mice learn successfully while the trisomic ones fail, unless they are first treated with memantine, which rescues their learning ability. The dataset is divided into 4 classes: mice that were not stimulated to learn (*no learning*, 555 samples), control mice that learned (*normal*, 285 samples), not treated and stimulated trisomic mice that failed to learn (*failed*, 105 samples) and treated and stimulated trisomic mice that learned successfully (*rescued*, 135 samples).

**Figure 4:**
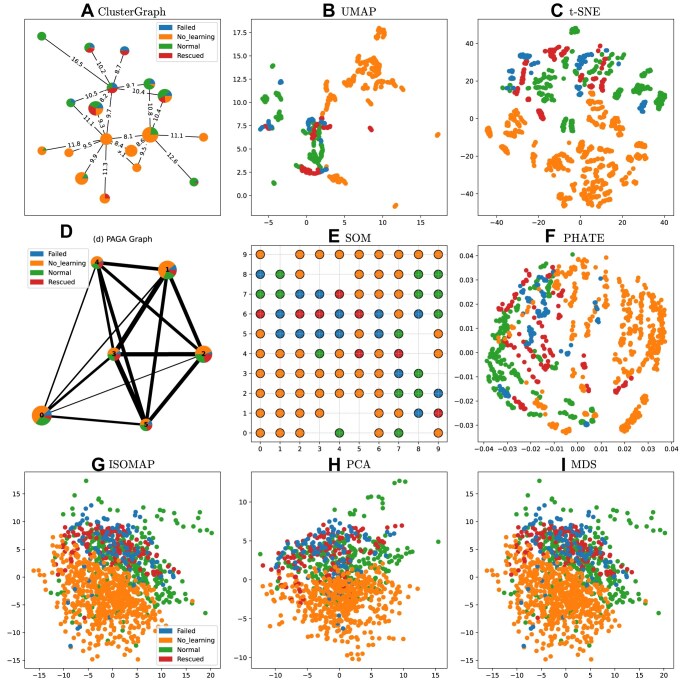
ClusterGraph visualization of the mice protein expression dataset alongside the output of other dimensionality reduction techniques. Each vertex in the ClusterGraph is depicted as a pie chart whose radius is proportional to the size of the corresponding cluster.

We reduce the dimensionality of the data by considering the first 31 principal components (95% of variance kept) and identify 18 clusters using $k$-means. The resulting ClusterGraph is depicted in Fig. 4, alongside the output of popular dimensionality reduction techniques. On most layouts, one can observe 2 main regions. One is almost entirely composed of *no learning* samples. The second one is dominated by the *normal* group and also contains the *rescued* and *failed* samples. State-of-the-art techniques such as UMAP and t-SNE are able to better separate the *no learning* samples from the others, but the 2 embeddings are drastically different. The ClusterGraph, on the other hand, captures the same information and displays it in a cleaner, embedding-agnostic way.

#### Assessing umap’s layout with ClusterGraph

ClusterGraph can also be applied to the output of any dimensionality reduction algorithm, in order to assess the quality of the returned low-dimensional embedding. As already stated, many of these methods aim to preserve the local structure of the point cloud, but they offer no guarantees on the global layout, as demonstrated in the following example.

**Figure 5:**
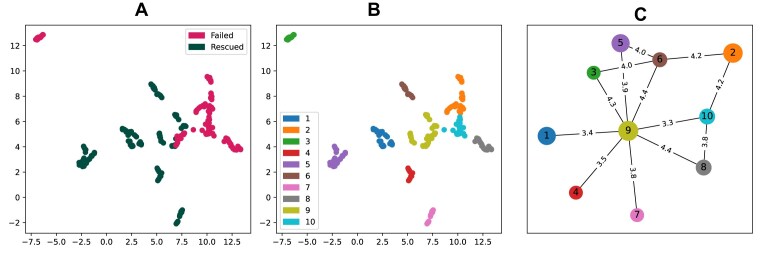
umap visualization of the *failed* and *rescued* samples is depicted in panels (A) and (B). Points are coloured by class in the former and by the output of a *k*-means clustering in the latter. Panel (C) depicts the ClusterGraph obtained from such clustering, where the distances are computed on the original space.

We focus our attention on the *failed* and *rescued* classes. Variable selection using a random forest method [5] was applied in order to identify the 10 most discriminating variables, this 10-dimensional point cloud is then visualized using umap (Fig. 5A). This projection is able to separate well the 2 classes, moreover, some points appear to be outliers.

In order to quantify this observation, 10 clusters are selected from the 2-dimensional embedding using $k$-means (Fig. 5B). A ClusterGraph is then built on top of them using the distance between clusters in the original 10-dimensional space. The connectivity pruned ClusterGraph is depicted in Fig. 5C, and it allows us to compare the organization of the points in the original space versus the low-dimensional umap embedding. In both visualizations, cluster 9 appears to be the central one, which is consistent with it being composed of a mixture of points from the 2 classes. We can however spot some clear differences between the 2 layouts. Cluster 3, which is the outlier on the top left of the umap plot, is not an outlier in the ambient space, as it is closer to cluster 9 than, e.g., cluster 6, which umap places close to the centre. Conversely, cluster 2, which appears to be near the centre in the umap plot, is at a significantly larger distance in the original space.

It is important to note that both visualizations agree with respect to the *local* layout, the differences appear at larger scales where umap fails to capture the global layout of the data.

#### Multi-level ClusterGraph

**Figure 6:**
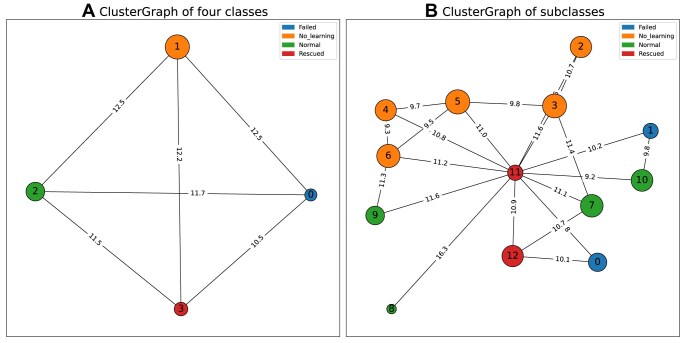
A simple ClusterGraph where each cluster corresponds to one of the 4 classes is depicted in panel (A). Each cluster can be further subdivided using $k$-means (with $k$ equal to 2 for rescued and failed, 4 for normal, and 5 for no-learning classes), the resulting connectivity pruned ClusterGraph is shown in panel (B). The radius of each vertex is proportional to the size of the corresponding cluster.

In some scenarios, we may be interested in further clustering a dataset, which is already partitioned. This is the case in our working example as each sample belongs to one of 4 classes: no learning, normal, failed, or rescued. A simple ClusterGraph obtained from such a coarse subdivision is depicted in Fig. 6A. Therefore, we could cluster samples of each class separately thus obtaining a finer partition that still respects the class labels, i.e., all clusters are monochromatic with respect to the class label. We partition the samples of each class into sub-clusters: 2 for rescued and failed, 4 for normal, and 5 for no-learning by applying $k$-means to the data projected into the first 31 principal components corresponding to 95% of the variance kept using PCA. The number of sub-clusters for each class is chosen based on the class-specific inertia.

A connectivity-pruned version of this ClusterGraph, obtained by removing 56 edges, is shown in Fig. 6B. Figure 6A shows that the rescued and failed classes are closely related, a relationship further confirmed by Fig. 6B. Although the no learning and normal classes appear as the most distant clusters in Fig. 6A, this relationship is more nuanced and can be explained by the presence of an outlier (cluster 8), while clusters 9 and 7 remain connected to the no learning nodes.

Note that the global layout is consistent with the ClusterGraph built on the full dataset without class labels, as shown in Fig. 6A. This multi-level approach allows us to obtain a clearer visualization by using the class labels as prior knowledge.

### Bone marrow mononuclear cells

The data analysed in this section consist of bone marrow mononuclear cells of healthy human donors [30] and were part of the NeurIPS 2021 OpenProblem benchmarking dataset. The dataset consists of the expression levels of 23,427 genes for 17,041 samples. After normalization, we identify the top 2,000 highly variable genes and compute the first 50 principal components. We then compute the neighbourhood graph of cells using the PCA representation of the data matrix and clustered it using the Leiden algorithm [31], separating it into 14 clusters.

**Figure 7:**
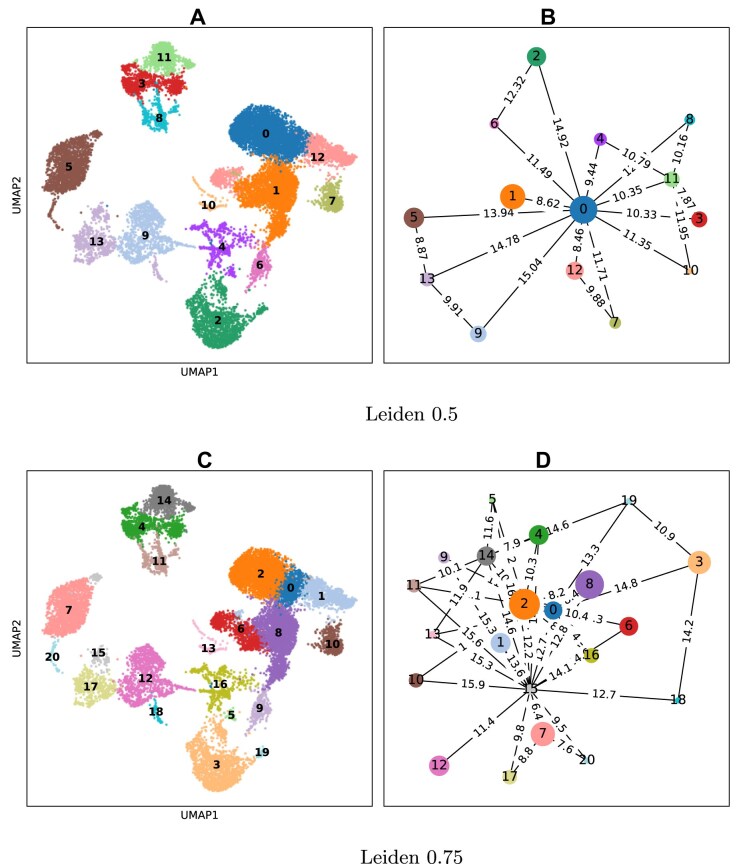
UMAP plots (A, C) and ClusterGraph (B, D) of the bone marrow single-cell dataset for 2 different Leiden thresholds. The colours correspond to the clusters found by the Leiden algorithm.

Figure 7 depicts in panel (A) the 2-dimensional UMAP projection of the first 50 principal components, coloured by the corresponding cluster. We then computed the ClusterGraph of the first 50 principal components, using as input the 14 clusters found by the Leiden algorithm. A connectivity-pruned ClusterGraph is shown in Fig. 7 panel (B), whose edge labels indicate the average distance between 2 clusters.

We can observe how both visualization techniques agree on the local organization of clusters, e.g., clusters 3, 8, 11 are close together, as well as clusters 5, 9, 13. At the same time, ClusterGraph allows us to gain insights into the global layout that are hidden by the 2-dimensional constraints of the UMAP plot. For example, cluster 6 is actually closer to cluster 0 than cluster 2.

### Human lung cancer cell lines

The last dataset [32] consists of single-cell RNA-seq of 5 human lung adenocarcinoma cell lines HCC827, H838, H2228, H1975, and A549. The 5 lung cancer cell lines were profiled using the 10X Chromium single-cell RNA sequencing platform.

**Figure 8:**
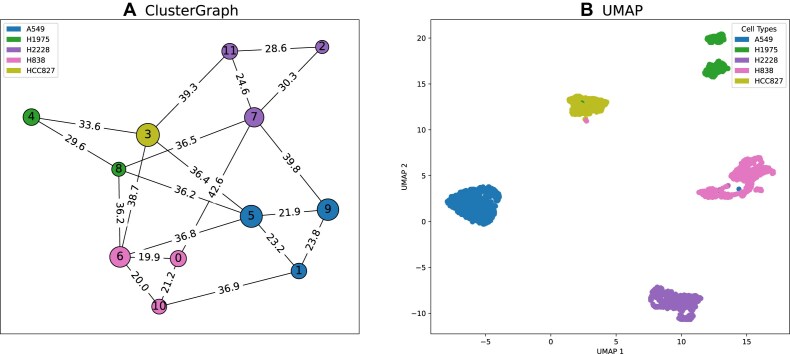
Visualization of the 5 cell line dataset using ClusterGraph and UMAP. (A) ClusterGraph representation generated using KMeans clustering with *k* = 12. Each node represents a cluster and is coloured according to its dominant cell population, defined as the label shared by at least 98% of the cells within the cluster. (B) UMAP projection. The colour scheme corresponds to the 5 cell lines: HCC827, H838, H2228, H1975, and A549.

We analyse the raw count matrix with genes represented as columns. A preprocessing pipeline is applied to retain only the most informative genes, focusing on those with high variability. PCA is then performed, and the top 40 principal components are retained for downstream analysis. The resulting dataset is visualized using both UMAP and ClusterGraph, as shown in Fig. 8.

In Fig. 8, the UMAP embedding reveals 5 clearly separated clusters, which largely correspond to the 5 cell lines. The green (H1975) and pink clusters (H838) appear closer together, while the blue (A549) cluster is positioned farther away. This organization suggests that the green (H1975) and pink (H838) cell populations are more similar to each other than either is to the blue one (A549). However, this interpretation can be further confirmed using ClusterGraph. By comparing cluster 8 (green cells) with cluster 5 (blue cells) and cluster 6 (pink cells), we observe that the distances from cluster 8 to both cluster 5 and cluster 6 are approximately equal. This indicates that the blue (A549) cluster is not an outlier, but is as distinct from the green (H1975) cluster as the pink (H838) cluster is. ClusterGraph thus provides a more balanced and interpretable view of the relationships between clusters that may not be fully captured in UMAP alone.

Another insight provided by the ClusterGraph is the ability to decompose each group into subgroups using algorithms such as KMeans as illustrated in Fig. 8A. For instance, in the UMAP embedding, the pink (H838) cluster appears more dispersed compared to the compact purple (H2228) cluster, which might suggest potential subpopulations or varying cellular states within the pink (H838) group. However, when examining the ClusterGraph, the distances between the purple (H2228) sub-clusters are actually greater than those within the pink cluster. This highlights how ClusterGraph can offer a more nuanced perspective on intra-cluster relationships that may not be immediately apparent in UMAP plots.

### ClusterGraph stability

The construction of ClusterGraph involves 2 key ingredients: a clustering scheme and a method for approximating geodesic distances. Because a systematic study of all possible choices is impractical, we focus on a single standard setting, using $K$-means for clustering and a $k$-NN graph for geodesic distance estimation, and examine how the behaviour of ClusterGraph changes as the corresponding parameters are varied over a suitable range. To avoid ambiguity, we write $k_{\mathrm{KNN}}$ for the number of neighbours in the $k$-NN graph used to approximate geodesic distances, and $K$ for the number of clusters in $K$-means.

#### Sensitivity to $k_{\mathrm{KNN}}$

The parameter $k_{\mathrm{KNN}}$ controls the connectivity of the $k$-NN graph used for geodesic distance estimation: too small a value risks graph disconnection and infinite geodesic distances, while too large a value introduces shortcuts between geometrically distant points, causing estimated distances to converge towards Euclidean distances and eroding manifold structure. The choice of $k_{\mathrm{KNN}}$ is therefore important, as the primary goal is to approximate geodesic distances faithfully while preserving the manifold structure of the data.

To assess the sensitivity of the ClusterGraph to $k_{\mathrm{KNN}},$ we run a grid search over $k_{\mathrm{KNN}}$ values ranging from 5 to 30 depending on the dataset, for several fixed values of $K.$ For each combination, a $k$-NN graph is constructed on the full point cloud, geodesic distances are estimated, and the metric distortion $\Delta _k(G)$ is recorded. This procedure is repeated across 4 datasets: **Mice Protein, Lung Cancer, Diabetes**, and **Concentric Circles**, covering both real biological data and synthetic geometric benchmarks.

**Figure 9:**
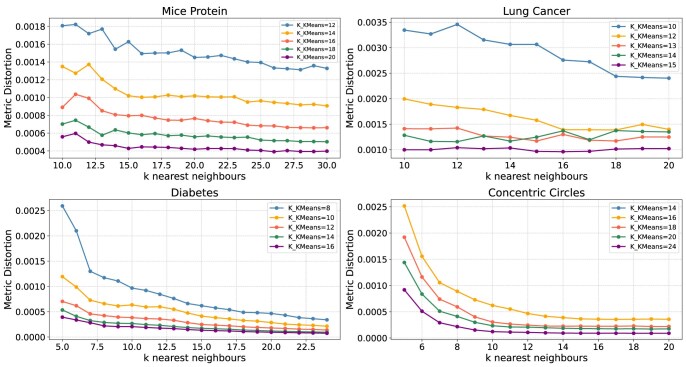
Sensitivity analysis on the **Mice Protein, Lung Cancer, Concentric Circles**, and **Diabetes** datasets as a function of the number of nearest neighbours, each line corresponding to a fixed value of $k_{\mathrm{KNN}}$.

Figure 9 shows the metric distortion as a function of $k_{\mathrm{KNN}}$, for several fixed values of $K$. A consistent pattern emerges across all datasets: metric distortion is higher and less stable for small values of $k_{\mathrm{KNN}}$, then decreases and stabilizes as $k_{\mathrm{KNN}}$ increases. This reflects the transition from an under-connected graph where geodesic estimates are noisy or unreliable, to a well-connected graph where distances stabilize. Beyond this stable region, increasing $k_{\mathrm{KNN}}$ further risks introducing shortcuts, degrading the geodesic approximation.

In practice, the recommended strategy is to identify the *elbow* of the distortion curve: the smallest $k_{\mathrm{KNN}}$ at which the metric distortion enters its stable plateau, balancing approximation quality against the risk of shortcut-induced bias. Since recomputing metric distortion over a dense grid of $k_{\mathrm{KNN}}$ values can be computationally expensive, the principled alternatives described below, which follow the same underlying logic, can guide this selection with lower overhead.

A natural first diagnostic for selecting $k_{\mathrm{KNN}}$ is to monitor the number of connected components of the $k$-NN graph as $k_{\mathrm{KNN}}$ increases. When several components persist despite increasing $k_{\mathrm{KNN}}$, this may indicate that the dataset is a union of multiple distinct manifolds, a structure that should be preserved during metric distortion pruning so that the resulting ClusterGraph faithfully reflects the geometry of the data. The relationships between these manifolds can then be explored through the merging process (“Merging” section).

The problem of selecting $k_{\mathrm{KNN}}$ automatically has been studied in the context of Isomap, leading to several principled approaches. Samko et al. [33] propose minimizing the residual variance of the Isomap embedding across multiple values of $k_{\mathrm{KNN}}$, while Jing and Shao [34] suggest monitoring the sum of shortest paths as $k_{\mathrm{KNN}}$ increases. Both methods look for an elbow in the resulting curve, identifying the smallest $k_{\mathrm{KNN}}$ beyond which the geodesic structure stabilizes, the same logic underlying the metric distortion plateau identified here. Once $k_{\mathrm{KNN}}$ is chosen, the quality of the geodesic approximation can be further improved by removing shortcut edges using betweenness-based filtering [35], which identifies edges that are disproportionately frequent in shortest paths and are therefore likely to represent spurious cross-manifold connections. Removing such shortcuts yields a cleaner $k$-NN graph, a more faithful geodesic approximation, and ultimately a more meaningful metric distortion.

In practice, we recommend $k_{\mathrm{KNN}} = 15$ as a default starting point, consistent with the literature on geodesic distance estimation, and we advise verifying the connectivity of the resulting $k$-NN graph as a sanity check.

#### Sensitivity to clustering granularity

To assess the sensitivity of the ClusterGraph to the parameter $K$ of $K$-means, we run a grid search over $K$ values ranging from 5 to 24 depending on the dataset, for several fixed values of $k_{\mathrm{KNN}}$. For each combination, a ClusterGraph is constructed, and the metric distortion $\Delta _k(G)$ is recorded. The same 4 datasets are used: **Mice Protein**  $(K \in [10, 20]$, $k_{\mathrm{KNN}} \in [15, 25]),$  **Lung Cancer**  $(K \in [10, 15],$  $k_{\mathrm{KNN}} \in [10, 20]),$  **Diabetes**  $(K \in [5, 15]$, $k_{\mathrm{KNN}} \in [8, 18]),$ and **Concentric Circles**  $(K \in [16, 24]$, $k_{\mathrm{KNN}} \in [8, 18])$ to evaluate consistency across both real biological data and synthetic geometric benchmarks.

**Figure 10:**
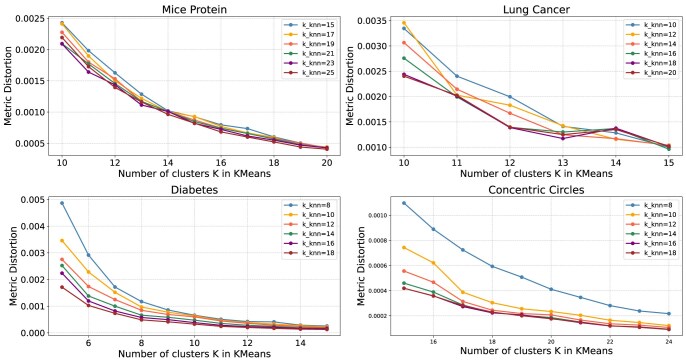
Sensitivity of the metric distortion to $K$, with $k_{\mathrm{KNN}}$ fixed, across 4 datasets: **Mice Protein** (top left), **Lung Cancer** (top right), **Diabetes** (bottom left), and **Concentric Circles** (bottom right). Each line corresponds to a fixed value of $k_{\mathrm{KNN}}$. Metric distortion decreases monotonically with $K$ across all conditions, confirming that finer clusterings consistently reduce geometric distortion.

Figure 10 indicates that the dependence of ClusterGraph on clustering granularity is stable, in the sense that changing $K$ does not produce abrupt changes in the score, but rather a gradual resolution-dependent effect.

#### Robustness to clustering noise

**Figure 11:**
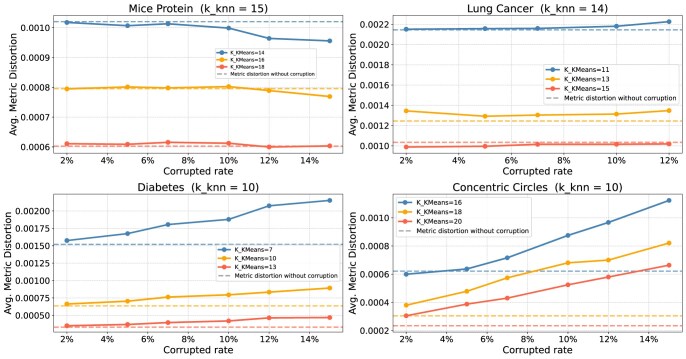
Robustness of the metric distortion to clustering label noise, across 4 datasets: **Mice Protein** ($k_{\mathrm{KNN}}=15$, top left), **Lung Cancer** ($k_{\mathrm{KNN}}=14$, top right), **Diabetes** ($k_{\mathrm{KNN}}=10$, bottom left), and **Concentric Circles** ($k_{\mathrm{KNN}}=10$, bottom right). Each solid line shows the average metric distortion over 10 independent corruptions at a given rate, for a fixed $K$. Dashed lines indicate the reference distortion with no corruption.

In practice, clustering assignments are never perfect: automated algorithms may mislabel at cluster boundaries, and biological noise can blur the separation between populations. To evaluate how sensitive ClusterGraph is to such imperfections, we simulate label noise by randomly reassigning a fraction $p$ of points to a different cluster chosen uniformly at random among all clusters other than their own. This corruption procedure is applied at rates $p \in \lbrace 2\%, 5\%, 7\%, 10\%, 12\%, 15\%\rbrace$, and for each rate, 10 independent random seeds are used to average out stochastic variation, yielding a mean metric distortion $\overline{\Delta }_k$ per condition. The $k$-NN graph is fixed at dataset-specific values ($k_{\mathrm{KNN}} = 15$ for **Mice Protein**, $k_{\mathrm{KNN}} = 14$ for **Lung Cancer**, and $k_{\mathrm{KNN}} = 10$ for **Diabetes**, and **Concentric Circles**), and 3 values of $K$ are tested per dataset. A dashed horizontal line in each panel of Fig. 11 indicates the reference metric distortion obtained without any corruption, serving as a baseline for comparison.

As shown in Fig. 11, the ClusterGraph exhibits strong robustness to label noise for most datasets and parameter settings: the average metric distortion remains close to the uncorrupted baseline even at corruption rates as high as $15\%$, with curves staying nearly flat across the full range of $p$. The effect is more pronounced for coarser clusterings (smaller $K$), where each cluster aggregates more cells and a few mislabelled points have a larger relative impact on inter-cluster distance estimates. Finer clusterings are more stable, as individual corrupted points represent a smaller fraction of each cluster. Notably, the **Concentric Circles** dataset exhibits the highest sensitivity to label noise. This can be attributed to its structure: the dataset consists of 2 geometrically disconnected components, yet the corruption procedure was applied globally at the dataset level rather than independently within each component. As a result, corrupted points may be reassigned across components, introducing inter-component label swaps that would never arise from a realistic clustering error, and artificially inflating the measured distortion.

Furthermore, the corruption model used here represents a worst-case scenario: in practice, clustering errors are predominantly local, i.e., a cell is far more likely to be mislabelled into a neighbouring cluster than into a distant, unrelated one. The global random reassignment applied here therefore overestimates the true impact of label noise, suggesting that the ClusterGraph is even more robust in practice than Fig. 11 indicates.

## Discussion

ClusterGraph provides a simple, noise-robust, and effective framework for compressing and visualizing high-dimensional data. By representing clusters as vertices and inter-cluster relationships as weighted edges, it captures the large-scale organization of a dataset without forcing the data into a 2-dimensional Euclidean embedding. After pruning, the resulting graph yields a compact summary of the intrinsic metric structure of the data.

A central advantage of the method is that it comes with a built-in quality criterion. Metric distortion quantifies how faithfully the graph reflects the intrinsic geometry of the dataset, thereby providing a principled basis for pruning, model selection, and parameter tuning.

ClusterGraph should be viewed as complementary to methods such as UMAP, t-SNE, and PHATE. Although these methods are often effective at preserving local neighbourhood structure, they can distort global relationships between data points. ClusterGraph addresses this limitation by encoding inter-cluster geometry directly in graph form. It therefore serves not only as a visualization tool, but also as a diagnostic layer that can validate, refine, or question conclusions drawn from low-dimensional embeddings.

## Additional files

The Supplementary_Appendix.pdf is available as supplementary material online.

## Availability of source code and requirements

- Project name: ClusterGraph
- Project homepage: https://github.com/dioscuri-tda/ClusterGraph
- Operating system: Linux, macOS, Windows
- Programming language: Python
- Other requirements: NumPy, pandas, NetworkX, Matplotlib, Bokeh, scikit-learn, POT (Python Optimal Transport)
- License: MIT license
- RRID: SCR 027405

## Supplementary Material

giag070_Supplementary_Appendix

giag070_Authors_Response_To_Reviewer_Comments_original_submission

giag070_GIGA-D-25-00347_original_submission

giag070_GIGA-D-25-00347_revision_1

giag070_Reviewer_1_Report_original_submission Reviewer 1 -- 12/27/2025

giag070_Reviewer_1_Report_revision_1 Reviewer 1 -- 4/26/2026

giag070_Reviewer_2_Report_original_submission Reviewer 2 -- 1/5/2026

## Data Availability

All real-world datasets used in this article are publicly available and can be found at Mice protein expression data [29]. Bone marrow mononuclear cells of healthy human donors [30]. Single-cell RNA-seq of 5 human lung cancer cell lines [32] with the GEO accession number GSM3618014. Diabetes Reaven and Miller [36].
