# Supplementary material for: ClusterGraph: a new tool for visualisation and compression of multidimensional data
Source: Gigascience. 2026 Jun 13;15:giag070. doi: 10.1093/gigascience/giag070 (PMC13354933; doi:10.1093/gigascience/giag070)
Supplement: giag070_Supplementary_Appendix [file giag070_supplementary_appendix.pdf]

A Complexity Analysis

We analyse the complexity of ClusterGraph's pipeline for $|X|$ points in $\mathbb{R}^d$, n clusters, and k, the number of neighbours in the k-NN graph.

A.1 ClusterGraph Construction

The construction complexity depends on the chosen inter-cluster metric. With centroid-based distances, computing each centroid requires a single pass over the cluster points at cost $O(|X|d)$, and evaluating all pairwise centroid distances costs $O(n^2 d)$, giving a total of $O(|X|d + n^2 d)$. When using average, minimum, or maximum inter-cluster distances, all pairwise point distances across every cluster pair must be evaluated, costing $O(|X|^2)$ regardless of cluster size distribution.

A.2 Metric Distortion

Computing the metric distortion requires building a k-NN graph over $|X|$ points and running all-pairs shortest paths (APSP) to populate the full $|X| \times |X|$ intrinsic distance matrix d_X^k. The k-NN graph construction costs $O(|X|^2 d)$ with exact methods, or $O(|X| \log |X|)$ with approximate methods such as HNSW [27]. Once the graph is built, Dijkstra's algorithm with a Fibonacci heap [37] computes APSP at a cost of $O(|X|^2 k + |X|^2 \log |X|)$. Combining both steps gives:

$$\text{Exact:} \quad O\big(|X|^2 (d + k + \log |X|)\big) \tag{17}$$

$$\text{Approximate:} \quad O\big(|X|^2 (k + \log |X|)\big) \tag{18}$$

Since APSP dominates k-NN graph construction in the approximate case, the overall complexity of the Metric Distortion for a given ClusterGraph reduces to $O\big(|X|^2 \log |X|\big)$, treating k and d as constants.

The Metric Distortion pruning step's complexity depends on both the chosen pruning algorithm and the initial graph structure. As described in Section 2.5.1, starting from a k'-NN–based ClusterGraph rather than the fully connected graph can significantly reduce the number of edges to prune.

A.3 Landmark-based approximation

To reduce complexity, we approximate ClusterGraph using a landmark set selected by the MaxMin algorithm. For a set of m landmarks over $|X|$ points, MaxMin costs $O(|X|m)$. Applying it globally and within each cluster (see Section 2.5.2) yields a total landmark selection cost of:

$$O\left(|X|\sqrt{|X|} + \sum_{i=1}^{n} n_i \sqrt{n_i}\right) = O\big(|X|^{3/2}\big). \tag{19}$$

The resulting reduced dataset X' contains $O(\sqrt{n|X|})$ points. The subsequent pipeline on X' costs $O(\sqrt{n|X|}\, d + n^2 d)$ for centroid distance computation and $O\big(n|X|(k + \log\sqrt{n|X|})\big)$ for the approximate k-NN graph and APSP. Since $n \ll |X|$, both terms are absorbed into $O\big(|X|^{3/2}\big)$, giving an overall landmark pipeline complexity of $O\big(|X|^{3/2}\big)$.

A.4 Comparison with related methods

Table 1 compares ClusterGraph's complexity against standard dimensionality reduction methods. The landmark approximation achieves $O(|X|^{3/2})$, sitting between exact ClusterGraph and UMAP.

**Method**	**Complexity**
Isomap [20]	$O(\lvert X\rvert^3)$
ClusterGraph exact	$O(\lvert X\rvert^2 \log \lvert X\rvert)$
t-SNE [2]	$O(\lvert X\rvert^2)$
ClusterGraph landmark	$O(\lvert X\rvert^{3/2})$
UMAP [3]	$O(\lvert X\rvert^{1.14})$

Table 1: Complexity comparison of ClusterGraph and standard dimensionality reduction methods. d is treated as a constant throughout.
