# Supplementary material for: ClusterGraph: a new tool for visualisation and compression of multidimensional data
Source: Gigascience. 2026 Jun 13;15:giag070. doi: 10.1093/gigascience/giag070 (PMC13354933; doi:10.1093/gigascience/giag070)
Supplement: giag070_GIGA-D-25-00347_original_submission [file giag070_giga-d-25-00347_original_submission.pdf]

GigaScience

ClusterGraph: a new tool for visualization and compression of multidimensional data
--Manuscript Draft--

**Manuscript Number:**	GIGA-D-25-00347
**Full Title:**	ClusterGraph: a new tool for visualization and compression of multidimensional data
**Article Type:**	Research
**Funding Information:**	Bundesministerium für Bildung und Forschung — Dr Paweł Dłotko<br>Narodowe Centrum Nauki — Dr Paweł Dłotko
**Abstract:**	Understanding the organization of high dimensional data is of primary interest for many branches of applied sciences. It is typically achieved by applying dimensionality reduction techniques which, while preserving local features, often miss the global structure of the dataset. Clustering techniques are another class of methods operating in the ambient space, grouping together similar points. However, unlike dimensionality reduction techniques, they do not provide information about organization of the data. Leveraging ideas from Topological Data Analysis, in this paper we provide an additional layer on the output of any clustering algorithm. Such data structure, ClusterGraph, provides information about the global layout of clusters, obtained from the considered clustering algorithm. Appropriate measures are provided to assess the quality and usefulness of the obtained representation. Subsequently the ClusterGraph, possibly with an appropriate structure–preserving simplification, can be visualized and used in synergy with state of the art exploratory data analysis techniques.
**Corresponding Author:**	Mathis Hallier, M.D<br>Instytut Matematyczny Polskiej Akademii Nauk<br>Warsaw, POLAND
**Corresponding Author Secondary Information:**	
**Corresponding Author's Institution:**	Instytut Matematyczny Polskiej Akademii Nauk
**Corresponding Author's Secondary Institution:**	
**First Author:**	Mathis Hallier, M.D
**First Author Secondary Information:**	
**Order of Authors:**	Mathis Hallier, M.D<br>Davide Gurnari, Phd<br>Anna Jurek-Loughrey, Phd<br>Paweł Dłotko, Phd
**Order of Authors Secondary Information:**	
**Additional Information:**	
**Question**	**Response**
Are you submitting this manuscript to a special series or article collection?	No
**Experimental design and statistics**<br><br>Full details of the experimental design and statistical methods used should be given in the Methods section, as detailed in our	Yes

Powered by Editorial Manager® and ProduXion Manager® from Aries Systems Corporation

Minimum Standards Reporting Checklist. Information essential to interpreting the data presented should be made available in the figure legends.<br><br>Have you included all the information requested in your manuscript?	
**Resources**<br><br>A description of all resources used, including antibodies, cell lines, animals and software tools, with enough information to allow them to be uniquely identified, should be included in the Methods section. Authors are strongly encouraged to cite Research Resource Identifiers (RRIDs) for antibodies, model organisms and tools, where possible.<br><br>Have you included the information requested as detailed in our Minimum Standards Reporting Checklist?	Yes
**Availability of data and materials**<br><br>All datasets and code on which the conclusions of the paper rely must be either included in your submission or deposited in publicly available repositories (where available and ethically appropriate), referencing such data using a unique identifier in the references and in the “Availability of Data and Materials” section of your manuscript.<br><br>Have you have met the above requirement as detailed in our Minimum Standards Reporting Checklist?	Yes
GigaScience has policies and guidelines in place for the use of generative AI-writing tools such as ChatGPT. If you have used such writing tools to assist with writing the manuscript this must be declared and cited in the text. Authors should not list AI-writing tools and other	No

AI-assisted technologies as an author or co-author and should acknowledge that they are fully responsible for text generated or refined by AI-writing tools.<p><br><br>A summary of use (particularly in the introduction or among methods) needs to be included at the end of the paper, and the outputs should also be included as a supplementary file hosted in GigaDB or other open repositories. Please read our guidelines for more information. <p><br><br>By submitting to GigaScience, you are aware of the journal's AI-writing tools policy, and if you have declared use of such tools below, you have acknowledged this where appropriate in your manuscript and have made a summary of use and outputs available. <p><br><b>AI-assisted writing tools have been used in the preparation of this manuscript?	

Powered by Editorial Manager® and ProduXion Manager® from Aries Systems Corporation

Manuscript Click here to access/download;Manuscript;ClusterGraph.pdf

ClusterGraph: a new tool for visualization and compression of multidimensional data

Paweł Dłotko[1], Davide Gurnari[1*], Mathis Hallier[1,2*], Anna Jurek-Loughrey[3]

[1]Dioscuri Centre in Topological Data Analysis, Mathematical Institute, Polish Academy of Sciences, Warsaw, PL.
[2]Génie Informatique, Université de Technologie de Compiègne, Compiègne, FR.
[3]School of Electronics, Electrical Engineering and Computer Science, Queens University of Belfast, Belfast, UK.

*Corresponding author(s). E-mail(s): dgurnari@impan.pl; mathis.hallier28@gmail.com;
Contributing authors: pdlotko@impan.pl; a.jurek@qub.ac.uk ;

Abstract

Understanding the organization of high dimensional data is of primary interest for many branches of applied sciences. It is typically achieved by applying dimensionality reduction techniques which, while preserving local features, often miss the global structure of the dataset. Clustering techniques are another class of methods operating in the ambient space, grouping together similar points. However, unlike dimensionality reduction techniques, they do not provide information about organization of the data.

Leveraging ideas from Topological Data Analysis, in this paper we provide an additional layer on the output of any clustering algorithm. Such data structure, *ClusterGraph*, provides information about the global layout of clusters, obtained from the considered clustering algorithm. Appropriate measures are provided to assess the quality and usefulness of the obtained representation. Subsequently the ClusterGraph, possibly with an appropriate structure–preserving simplification, can be visualized and used in synergy with state of the art exploratory data analysis techniques.

1 Introduction

High-throughput experiments are becoming extremely common in applied sciences. Now more than ever, large high-dimensional datasets are generated in almost every laboratory calling for an automated and reliable way to extract new knowledge from them. Let us fix a dataset X, usually embedded in a high dimensional space. Standard dimension reduction techniques, including PCA [1], t-SNE [2], UMAP [3] and PHATE [4] aim to find a low dimensional embedding of X so that points that are close in X, are also close in the embedding. However, preservation of the global organization of X in general, and information about distances of far away points in particular, is a challenge for this methods.

Clustering techniques [5, 6] on the other hand, based on a fixed similarity measure, provide a partition of the input dataset X. However, clustering itself does not provide information about either intra– or inter– cluster organization of points, therefore is not used to asses the global structure of the data. The aim of this work is two bridge this two approaches by enriching the output of a clustering algorithm with additional information on the data's global organization.

The first contribution of this paper is the construction of a *ClusterGraph*; a graph–based structure on top of a partition $\mathcal{C}(X)$ of the data obtained from a clustering algorithm $\mathcal{C}$ applied to X. In the ClusterGraph $G = (V, E)$, each vertex corresponds to a single cluster from $\mathcal{C}(X)$. Two vertices $u, v \in V$ are connected by an edge whose length corresponds to the distance between their respective clusters in $\mathcal{C}(X)$. For the purpose of this construction, a number of inter–cluster distances defined in the ambient space are considered.

ClusterGraph has a number of advantages compared to alternative dimension reduction methods. One of them is based on the fact that the distances, computed in the ambient space, are represented by labels on edges and not subjected to distortions made by standard dimension reduction techniques that force the projected data points to be embedded in an Euclidean space. This allows to visualize the *global* distances in the dataset. This is important as many datasets cannot be embedded into low dimensional Euclidean spaces without perturbing the distances between points.

As an example of such a situation consider a collection of points in four clusters: 0, 1, 2 and 3. Points in each cluster are infinitesimally close. Distance between cluster 0 and clusters 1, 2 and 3 are 1, while distance between clusters 1, 2 and 3 is 2. It is well known [7, 8] that such a graph cannot be isometrically embedded to any Euclidean space $\mathbb{R}^n$ for any n. As a consequence, all dimension reduction techniques will distort the distances between clusters, as can be observed in Fig 1. ClusterGraph, on the contrary, provides the correct graph even in this case.

The second contribution is a method to assess the quality of the ClusterGraph G.

Working under assumption that X is sampled from a manifold equipped with an intrinsic distance, a *metric distortion* between the intrinsic distances on X and the distance induced by G on X is used to assess the quality of G. The distance between points $x, y \in X$ induced by G is the length of a shortest path in G between vertices representing the clusters containing them.

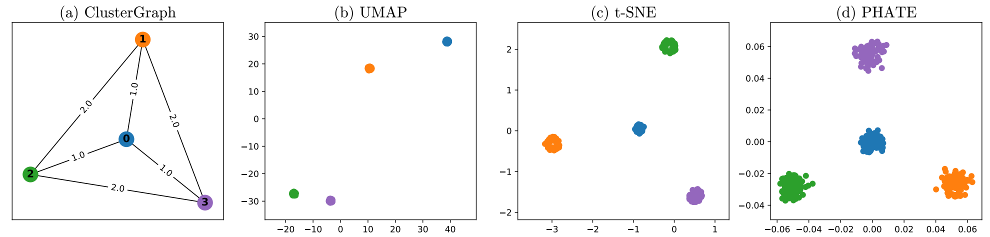

Fig. 1: The dataset consisting of four clusters 0 (blue), 1 (orange), 2 (green) and 3 (purple), as described in the text, so that elements of cluster 0 are distance one from elements from the remaining clusters and the mutual distances between elements of clusters $1, 2, 3$ are two. Such a dataset cannot be embedded, with the distances preserved, to any Euclidean space. In this case, UMAP (panel b) fails to capture the global layout, while t-SNE (panel c) and PHATE (panel d) do. However the coordinate systems of t-SNE and PHATE are drastically different. In both cases, as a result of the embedding into the Euclidean plane, the ratio of the distances $d(1, 2)/d(0, 1)$ is roughly $\sqrt{3}$ instead of the original 2, the same is true for the other clusters. This is the optimal embedding that can be achieved when points are projected to Euclidean space. However, in the case of ClusterGraph (panel a), the distances are encoded as labels to the graph edges and therefore we are not restricted by any Euclidean coordinate system.

The logarithm of the ratio between the intrinsic and the ClusterGraph distance is used as a quality measure: the smaller its value, the better the quality of the ClusterGraph representation.

This procedure will be used to obtain a *pruned ClusterGraph* that better approximates the intrinsic structure of the data. For this purpose, a number of *edge pruning* algorithms are proposed aiming to remove some edges while maintaining the global structure of the data. A schema of the whole ClusterGraph pipeline is depicted in Figure 2.

A Python implementation of the creation of ClusterGraph data structure, as well as the pruning algorithms and interactive visualization utilities is available at github.com/dioscuri-tda/ClusterGraph.

2 Methods

2.1 ClusterGraph

Let X be a dataset equipped with a metric $d_X : X \times X \to \mathbb{R}_{\geq 0}$. Take $\mathcal{C}$ to be an arbitrary hard or soft clustering algorithm. Let $\mathcal{C}(X)$ be the partition or, in a more general case, a division of X obtained from the clustering algorithm $\mathcal{C}$.

A collection of sets $\{C_i\}$ is a *partition* of X if for every $C_i \neq C_j \in \mathcal{C}(X)$, $C_i \cap C_j = \emptyset$ and $\bigcup_{C_i \in \mathcal{C}(X)} C_i = X$. Partitions of X are typically obtained from hard clustering algorithms [5]. In a more general case, we can drop the empty intersection condition turning $\mathcal{C}(X)$ into a *division* of X. Divisions may be obtained using soft or fuzzy

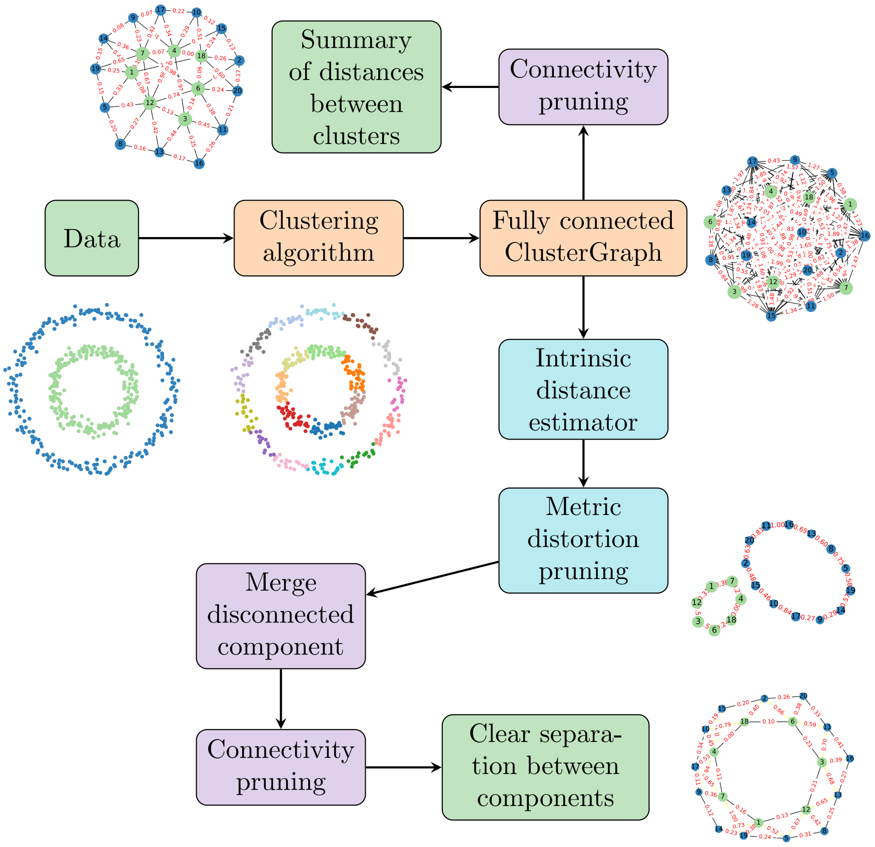

Fig. 2: ClusterGraph pipeline with the two possible pruning strategies. Details on the example dataset that has been used to generate the figures can be found in Section 3.1.

clustering methods [9]. They can also be obtained as byproducts of Topological Data Analysis techniques like Mapper [10] or Ball Mapper [11].

In the next step we extend d_X into a distance or similarity measure $d_{\mathcal{C}} : \mathcal{C}(X) \times \mathcal{C}(X) \to \mathbb{R}_{\geq 0}$ defined on elements of $\mathcal{C}(X)$, as detailed in Section 2.1.1. The *ClusterGraph* of a partition $\mathcal{C}(X)$ is a fully connected graph whose vertices are elements of $\mathcal{C}(X)$ and edges are weighted by distance $d_{\mathcal{C}}$ of the elements of $\mathcal{C}(X)$.

ClusterGraph is intended to serve as a tool for data visualization and compression. Compression is achieved by collapsing points within the same cluster into a vertex of the ClusterGraph. This step is motivated by an assumption that points within the same cluster are in close proximity, hence share mulipe characteristics and can be prepresent by a single vertex. We expect ClusterGraph to have much fewer vertices compared to the original number of points. The visualization aspect is accomplished when the layout of the ClusterGraph resembles, to some extent, the layout of the input point cloud. However, this is unlikely to be the case for a fully connected graph.

Therefore, in Section 2.2, we propose a *metric distortion*-based criterion and techniques for removing certain edges of the ClusterGraph so that the metrics on the graph and on the initial point cloud become comparable. Different strategies for edge removal from the ClusterGraph are discussed in Section 2.3. This process yields a *pruned* ClusterGraph, which we use for visualization purposes.

2.1.1 Distances between clusters

In this section, for a dataset X equipped with similarity measure d_X and a partition $\mathcal{C}(X)$, a number of similarity measures $d_{\mathcal{C}} : \mathcal{C}(X) \times \mathcal{C}(X) \to \mathbb{R}_{\geq 0}$ are presented. The choice of the optimal one is application dependent, very much like the clustering algorithm $\mathcal{C}$, and should therefore be selected and optimized by the user. Given two clusters C_i and C_j, possible options include:

1. Maximum, minimum or an average distance between points

$$\min(C_i, C_j) = \min_{x \in C_i, y \in C_j} d_X(x, y)$$
$$\max(C_i, C_j) = \max_{x \in C_i, y \in C_j} d_X(x, y)$$
$$\operatorname{avg}(C_i, C_j) = \sum_{x \in C_i} \sum_{y \in C_j} d_X(x, y)/(|C_i||C_j|).$$

2. Hausdorff distance

$$d_H(C_i, C_j) = \max \left\{ \sup_{x \in C_i} d(x, C_j), \sup_{y \in C_j} d(C_i, y) \right\},$$

where $d(a, B) = \inf_{b \in B} d(a, b)$.

3. Earth mover (a.k.a. Wasserstein) distance [12, 13] which utilizes ideas from probability theory and optimal transport

$$W_p(C_i, C_j) = \inf_{\eta: C_i \to C_j} \left(\sum_{x \in C_i} d_X(x, \eta(x))^p \right)^{\frac{1}{p}},$$

where η is a matching between points of C_i and C_j and $1 \leq p < \infty$. If the two clusters have different size, the matching is computed in a weighted way, i.e. each point in C_i is assigned a weight of $1/|C_i|$ such that each cluster has a total mass of 1.

2.1.2 Stability

Let us consider a dataset X , and two partitions of it $\mathcal{C}(X)$, $\mathcal{D}(X)$ obtained via some clustering algorithms. We are interest in quantifying how different these two partitions can be. In order to do so we introduce the following concept.

Definition 1 (Image of a cluster) Let X be a dataset and $\mathcal{C}(X)$, $\mathcal{D}(X)$ two partitions of it. The *image* of a cluster $C_i \in \mathcal{C}(X)$ in $\mathcal{D}(X)$ is the union of all clusters of $\mathcal{D}(X)$ that contain some points of C_i, namely $\mathrm{im}_{\mathcal{D}(X)}(C_i) = \{\bigcup D_j \in \mathcal{D}(X) \mid C_i \cap D_j \neq \emptyset\}$.

This idea of mapping the points covered by one cluster in a given partition to the clusters in a second partition is inspired by an analogous technique for mapper graphs, *MappingMappers*, described in [14].

Let us define the *diameter* of a collection of points as the greatest distance between any pair of points. We can then state the following bound.

Proposition 1 (Clustering stability) *Let X be a dataset and $\mathcal{C}(X)$, $\mathcal{D}(X)$ be two partitions of it such that the diameter of each set in $\mathcal{C}(X)$ and $\mathcal{D}(X)$ is at most δ. Then, for any cluster $C_i \in \mathcal{C}(X)$, its image in $\mathcal{D}(X)$ has diameter at most 3δ.*

Proof Let $d_1, d_2 \in X$ be the two points whose distance realizes the diameter of $\mathrm{im}(C_i)$. By definition of image there is at least one point $c_1 \in C_i$ which lies in the same cluster of $\mathcal{D}(X)$ as d_1, and similarly there exist at least one c_2 for d_2. Therefore we have

$$d_X(d_1, d_2) \leq d_X(d_1, c_1) + d_X(c_1, c_2) + d_X(c_2, d_2) \leq 3\delta\ .$$

□

Using a summary statistic of the distance between points as distance between clusters (option 1 in 2.1.1) allows us to derive a similar bound for ClusterGraphs built on top of $\mathcal{C}(X)$ and $\mathcal{D}(X)$.

Definition 2 (Image of a ClusterGraph vertex) Let X be a dataset, $\mathcal{C}(X)$, $\mathcal{D}(X)$ two partitions of it and $G_{\mathcal{C}(X)}$, $G_{\mathcal{D}(X)}$ the ClusterGraphs obtained from $\mathcal{C}(X)$ and $\mathcal{D}(X)$. The *image* of a vertex $i \in G_{\mathcal{C}(X)}$ (corresponding to cluster $C_i \in G_{\mathcal{C}(X)}$) in $G_{\mathcal{D}(X)}$ is the collection of all vertices of $G_{\mathcal{D}(X)}$ that corresponds to clusters in $G_{\mathcal{D}(X)}$ containing some points of C_i.

We define the *diameter* of a weighted graph as the greatest distance between any pair of vertices.

Proposition 2 (ClusterGraph stability) *Let X be a dataset, $\mathcal{C}(X)$, $\mathcal{D}(X)$ two partitions of it and $G_{\mathcal{C}(X)}$, $G_{\mathcal{D}(X)}$ the ClusterGraphs obtained from them. Assume that the diameter of each set in $\mathcal{C}(X)$ and $\mathcal{D}(X)$ is at most δ. Then the image of each vertex $u \in G_{\mathcal{C}(X)}$ in $G_{\mathcal{D}(X)}$ is a clique of diameter at most 3δ for the maximum and average distance, and δ for the minimum.*

Proof Let u be a vertex in $G_{\mathcal{C}(X)}$ and $\mathrm{im}(u)$ its image in $G_{\mathcal{D}(X)}$.

Recall that $\mathrm{im}(u)$ is a subset of the complete graph $G_{\mathcal{D}(X)}$, therefore it is a clique. Let D_i and D_j be the clusters in $\mathrm{im}(u)$ whose distance realizes the diameter of $\mathrm{im}(u)$, i.e. they correspond to the two vertices in the clique that are the furthest apart.

Let us start with the maximum distance case. In particular, let $d_1 \in D_i$ and $d_2 \in D_j$ be the two data points realizing the maximum distance between D_i and D_j, and therefore $d(d_1, d_2) = \text{im}(u)$. Let C_u be the cluster in $\mathcal{C}(X)$ corresponding to vertex $u \in G_{\mathcal{G}(X)}$. By definition of image of u there are at least two points $c_1, c_2 \in C_u$ which lie in the same clusters of $\mathcal{D}(X)$ as d_1 and d_2, respectively. We can then proceed in a similar fashion to the proof of Proposition 1, namely we have

$$\text{diam}(\text{im}(u)) = \max(D_i, D_j) = d_X(d_1, d_2) \leq d_X(d_1, c_1) + d_X(c_1, c_2) + d_X(c_2, d_2) \leq 3\delta\ .$$

The same bound holds for the average distance since $\text{avg}(D_i, D_j) \leq \max(D_i, D_j)$.

For the minimum distance case it is sufficient to notice that $d_X(c_1, c_2) \leq \delta$ because they both belong to the same cluster C_i whose diameter is bounded by δ. Hence we have

$$\text{diam}(\text{im}(u)) = \min(D_i, D_j) \leq d_X(c_1, c_2) \leq \delta.$$

□

2.2 Metric distortion

Given a dataset X, different choices of the clustering algorithm $\mathcal{C}$, as well as the metrics d_X and $d_{\mathcal{C}}$ can lead to significantly different ClusterGraphs. The aim of this section is to introduce a score to assess the quality of a given ClusterGraph G by comparing it to the underlying geometric structure of the dataset X.

For this purpose let us assume that the considered point cloud X is sampled from a compact and connected manifold $\mathcal{M}$ equipped with an *intrinsic distance* $d_{\mathcal{M}}$. Informally, the intrinsic distance between two points $x, y \in \mathcal{M}$ is defined to be the infimum of the length of a curve $\gamma \subset \mathcal{M}$ joining x and y, this is also known as *geodesic* distance.

In most applications, the underlying manifold is not known. Consequently, the intrinsic distance needs to be estimated from the point cloud. This is a well-studied problem in computational geometry and computer graphics, and multiple methods have been proposed [15–18].

Below, we follow the approach of [15, 19] using the shortest path in the k-nearest neighbor graph as estimator. Note that any other estimator of intrinsic distance can be also used in the proposed construction.

Let $G_{knn}(X)$ be the k-nearest neighbor graph on X constructed in the following way: each point of X corresponds to a vertex of $G_{knn}(X)$; it is connected to its k-nearest neighbors (in the chosen distance d_X, typically Euclidean), with k being a parameter of the method. Weights corresponding to the distance between endpoints are assigned to the edges of $G_{knn}(X)$. We define a distance d^k_X on $G_{knn}(X)$, estimating the intrinsic distance on X, as

$$d^k_X(x, y) = \text{the length of the shortest path between } x \text{ and } y \text{ in } G_{knn}(X) \qquad (1)$$

Remark 1. It may happen that $G_{knn}(X)$ is not connected. There are two possible reason for this. In the first case points are indeed sampled from a compact and connected manifold but the parameter k is too low. This can be easily solved by increasing k. In the second case the underlying manifold is not connected. This will result in the k-nn graph being disconnected even for very high values of k, especially if many points are sampled. In this case, we will threat each connected component

separately, splitting the input dataset X (and the output of the clustering algorithm) into disjoint sets, each one corresponding to a different connected component and analyze each of them separately[1]. For the rest of the Section we therefore assume, without lack of generality, that the k-nn graph is fully connected. We discuss how to investigate the relations between different connected components in Section 2.3.3.

Remark 2. Whenever an estimator is used, it is natural to ask how good such estimator is. The choice of a k-nn graph as an estimator of the geodesic distance is motivated by the following theorem by Bernstein, Vin de Silva, Langford and Tenenbaum.

Theorem 3 (Theorem A in [19]) *Let $\mathcal{M}$ be a compact submanifold of $\mathbb{R}^n$, X a finite set of data points in $\mathcal{M}$ and G a graph on X (for example, a k-nn graph). Then the inequalities*

$$(1-\lambda_1)d_{\mathcal{M}}(x,y) \leq d_G(x,y) \leq (1+\lambda_2)d_{\mathcal{M}}(x,y)$$

are valid for all x, y in X, where $\lambda_1, \lambda_2 < 1$ are two positive real numbers that depends on G, $\mathcal{M}$ and some technical assumptions on the density of X.

For each point $x \in X$, we denote with C_x the cluster in $\mathcal{C}(X)$ that contains x. We can then use the distance between clusters described in 2.1.1 to define a distance between points d_{CG} in the ClusterGraph G as following

$$d_{CG}(x,y) = \text{the length of the shortest path between } C_x \text{ and } C_y \text{ in } G. \tag{2}$$

Recall that G is fully connected; one could wonder why the length of the shortest path between two vertices is used instead of the weight of the edge connecting them given by $d_{\mathcal{C}}$. First, the triangle inequality might not hold for $d_{\mathcal{C}}$. Secondly, we want the definition of d_{CG} to hold also in the case of a pruned ClusterGraph, which we will discuss in Section 2.3.

This notion of d_{CG} is well defined only when $\mathcal{C}(X)$ is a partition. In the more general case of a division, when a point can belong to more than one cluster, we take $d_{CG}(x,y)$ to be the length of the shortest path between any cluster containing x and any cluster containing y.

Consider the ClusterGraph $G = (V, E)$ and let us fix two vertices $i, j \in V$, corresponding to two clusters C_i and C_j. For a pair of points $x \in C_i$ and $y \in C_j$, $x \neq y$, we compute:

$$\delta(x,y) = |\log\left(\frac{d_{CG}(x,y)}{d^k_X(x,y)}\right)|. \tag{3}$$

The use of the absolute value of the logarithm ensures that a multiplicative scaling by a factor $\lambda = \frac{d_{CG}(x,y)}{d^k_X(x,y)}$ result in the same metric distortion as a $1/\lambda$ scaling, i.e. $|\log(x/y)| = |\log(y/x)|$ for every $x, y \in \mathbb{R}_{>0}$.

By averaging this quantity over all possible pairs of points $x \in C_i$ and $y \in C_j$, we obtain the metric distortion between clusters C_i and C_j.

[1]By performing the construction in Sec. 2.1 we obtain a ClusterGraph for each connected component, each of them being a fully connected graph. In graph theory such a disjoint union of complete graphs is sometimes called a "cluster graph". This unexpected but pleasing agreement in nomenclature motivates our choice of referring to our construction in camel case, to avoid confusion.

$$\delta_{\{i,j\}} = \frac{1}{|C_i||C_j|} \sum_{(x,y)\in(C_i,C_j)} \delta(x,y) \tag{4}$$

This score assesses how much the intrinsic distance between points on X differs from their corresponding distance in the ClusterGraph.

The global metric distortion of the considered ClusterGraph can be obtained by averaging the score for each pair of vertices defined in Equation 4. However, because clusters can have different sizes, we consider a weighted average. The weight of the pair $\{i,j\}$ corresponding to clusters C_i and C_j is defined as:

$$w_{\{i,j\}} = \frac{|C_i \cup C_j|}{(n-1)|X|}, \tag{5}$$

where n denotes the number of clusters in $\mathcal{C}(X)$ (or equivalently $n = |V|$, the number of vertices in the ClusterGraph). The rationale behind this averaging is to give more importance to interactions between large clusters. The global metric distortion for a ClusterGraph (with respect to the k-nearest neighbors graph) can then be defined as

$$\Delta_k(G) = \frac{2}{n(n-1)} \sum_{\{i,j\}\in V} w_{\{i,j\}}\delta_{\{i,j\}}. \tag{6}$$

This quantity is a non-negative real number indicating how well the given ClusterGraph respects the intrinsic metric structure of the data. It allows to compare the quality of ClusterGraphs having an equal, or very similar number of vertices. Comparison of metric distortions of ClusterGraphs having vastly different number of nodes should not be performed.

In the next sections we will use each edge's distortion as well as the global distortion to prune the ClusterGraph; with the goal of removing the edges that do not reflect the underlying structure of the data.

Remark 3. The ratio between two distances in our definition of the distortion (Eqn. 3) might be reminiscent of the *stretch factor* or *distortion* of an embedding f. For two given points x and y in a metric space the stretch factor is defined as $d(f(x), f(y))/d(x,y)$. For example, consider a set of points in $\mathbb{R}^d$ and a connected graph having those points as vertex set. Each edge in the graph has a weight corresponding to the Euclidean distance between its endpoints. The stretch factor for two given points is the ratio of the length of the shortest path between them in the graph to their Euclidean distance. The stretch factor of the graph is the maximum stretch factor over any pair of points. Graphs with stretch factor at most t are called *t-spanners* [20].

It is important to point out the differences between this widely studied topic in graph theory and our approach. First of all, we are not dealing with an embedding as the map that sends each data point to its cluster is highly non injective. Moreover, the stretch factor of a graph defined in the paragraph above is always greater than or equal to 1. In our setting, the ratio between the ClusterGraph distance and the

intrinsic one (Eqn. 3) might be less than 1, this is exactly the case of a "shortcut" edge in the ClusterGraph.

2.3 ClusterGraph pruning

ClusterGraph is, by definition, a fully connected graph. Consequently it may contain edges connecting regions of the datasets that are not close in the manifold $\mathcal{M}$ from which the data points of X are sampled. We will refer to these edges as "shortcuts", as they are shorter than the true geodesic distance between the corresponding points in the manifold and therefore are not representative of the underlying manifold structure. The removal of such edges will make the ClusterGraph more similar, in the sense of metric distortion, to X. Moreover, the ClusterGraph may also contain edges the removal of which does not change considerably the metric structure of the graph. In this section we introduce three possible ideas to prune edges of a given ClusterGraph, and by doing so, of increasing the quality of the obtained representation.

2.3.1 Threshold pruning

If the triangle inequality holds for the distance between clusters $d_{\mathcal{C}}$, the length of the shortest path between each pair of vertices in the ClusterGraph (Eqn. 2) is exactly the length of the edge connecting them. It makes sense then to assign to each edge in the ClusterGraph the metric distortion for its two corresponding clusters, as defined in Equation 4.

The ClusterGraph can then be naively pruned by removing all edges having metric distortion greater than a threshold $\alpha > 0$.

2.3.2 Iterative greedy pruning

Consider the ClusterGraph $G = (V, E)$. Let $\Delta_k(G)$ be its metric distortion as in Equation 6. Denote with $G_{\hat{e}}$ the ClusterGraph obtained by removing edge e from G, namely $G_{\hat{e}} = (V, E \setminus \{e\})$.

We can then perform the following iterative greedy pruning procedure. Remove edge e if both conditions hold:

1. $\Delta_k(G_{\hat{e}}) \leq \Delta_k(G)$,
2. $\Delta_k(G_{\hat{e}}) \leq \Delta_k(G_{\hat{e}'})$ for any other $e' \in E$.

Then update E to $E \setminus \{e\}$. The process may be repeated a fixed number of times, or as long as such an edge e can be found. Note that the length of the shortest path between two vertices defined in Equation 2 will be infinite if the ClusterGraph becomes disconnected, thus leading to an infinite value of the metric distortion. Therefore condition (1) ensures that the pruning procedure will never produce new connected components of the ClusterGraph.

2.3.3 Connectivity based pruning

The first two pruning techniques presented in Sections 2.3.1 and 2.3.2 focus on the removal of the edges with high metric distortion or, informally speaking, the removal

all the "shortcuts" with respect to the structure of X. It might happen that after this pruning the obtained ClusterGraph still has a complicated structure which might render its visualization and interpretation challenging.

In what follows we adopt the *connectivity based* approach by Zhou, Mahler and Toivonen [21] to the ClusterGraph pruning. A *path* P in $G = (V, E)$ is a set of edges $P = \{\{i_1, i_2\}, \{i_2, i_3\}, \ldots, \{i_{k-1}, i_k\}\} \in E$. A *path quality function* $q(P) \to \mathbb{R}^+$ is defined by taking the sum of the inverse of the path length, calculated using the distance between clusters $d_{\mathcal{C}}$

$$q(P) = \sum_{\{i,j\} \in P} \frac{1}{d_{\mathcal{C}}(C_i, C_j)}. \tag{7}$$

The *connectivity* between two vertices i, j in $G = (V, E)$ is the quality of the best path between them

$$\operatorname{conn}(i, j; E) = \begin{cases} \max_{P \in \mathcal{P}(i,j)} q(P) & \text{if } \mathcal{P}(i,j) \neq \emptyset \\ -\infty & \text{otherwise} \end{cases} \tag{8}$$

where we denoted by $\mathcal{P}(i, j)$ the set of all possible paths between vertices i and j. The *connectivity of a ClusterGraph* is the average connectivity over all pairs of vertices

$$\operatorname{conn}(V, E) = \frac{2}{n(n-1)} \sum_{i,j \in V, i \neq j} \operatorname{conn}(i, j; E), \tag{9}$$

where n is the number of vertices in the ClusterGraph. Note that the connectivity will be $-\infty$ if G is disconnected. In that case each connected component should be analyzed separately.

Let us now consider the ClusterGraph with one edge removed $G_{\hat{e}} = (V, E \setminus \{e\})$. It is straightforward to see that $\operatorname{conn}(V, E \setminus \{e\}) \leq \operatorname{conn}(V, E)$. In particular $\operatorname{conn}(V, E \setminus \{e\}) = \operatorname{conn}(V, E)$ if and only if e does not belong to any of the best paths between any pairs of vertices. Moreover, $\operatorname{conn}(V, E \setminus \{e\}) = -\infty$ if the removal of e disconnects the graph. We can then define the *ratio of connectivity kept* after removing an edge or, more generally, after removing a subset of edges $E_R \subset E$

$$rk(V, E, E_R) = \frac{\operatorname{conn}(V, E \setminus E_R)}{\operatorname{conn}(V, E)}. \tag{10}$$

Pruning can then be executed in a greedy iterative fashion (see Alg. 2 BF in [21]) by selecting, at each iteration, the edge whose the removal will result in smallest decrease of connectivity, i.e. the largest rk value.

2.4 Merging

As discussed in Remark 1, it might happen that the underlying manifold from which the data are sampled is disconnected. In that case, the resulting ClusterGraph will have

more than one connected component and each of them will be pruned separately. In order to capture the global layout of the data, including the disconnected components of the manifold, we may merge different components by adding a collection of special edges between each vertex v and its k-nearest neighbors not belonging to the same connected component as v. Subsequently, the connectivity based pruning procedure can be applied to the newly added edges.

3 Results

3.1 Concentric circles

To showcase the whole ClusterGraph pipeline (Figure 2) we considered 500 points sampled from two concentric circles in the plane, depicted in Figure 3. Clusters were computed using k-means with 20 centroids and a ClusterGraph was built using the average Euclidean distance between points. The 10-nearest neighbors graph was used to estimate the intrinsic distance between data points. The iterative metric distortion pruning procedure was applied and the pruned ClusterGraph is showed in Figure 3(b). Note that the pruned ClusterGraph has two connected components, as a consequence of the 10-nearest neighbors graph having two connected components. Finally, the two components were merged by adding an edge between each vertex and its 3-nearest neighbors in the other connected component. We then pruned 20 of these newly introduced edges using the connectivity based approach. The resulting ClusterGraph is depicted in Figure 3(c).

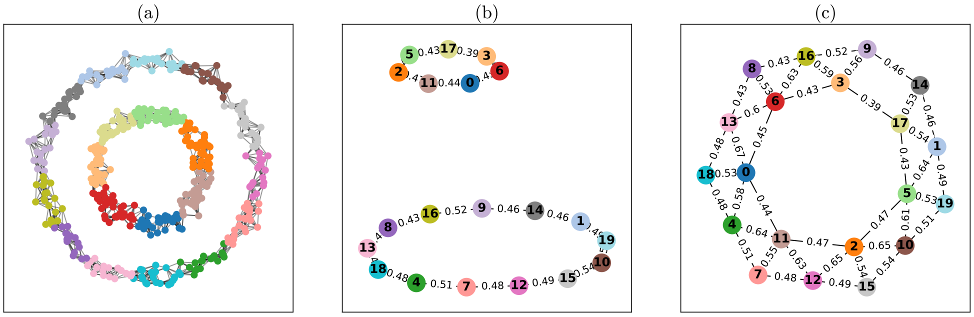

Fig. 3: ClusterGraph built on the output of k-means for 500 points sampled from two concentric circles. The 20 clusters are depicted in (a), on top of the 10-nn graph. The two metric distortion-pruned components are depicted in (b) and subsequently merged and connectivity pruned (c). The vertices' colors are inherited from panel (a).

3.2 Mice protein expression

We analyzed the expression levels of 77 proteins obtained from 38 normal genotype control mice and from 34 of their trisomic littermates, both with and without treatment with the drug memantine and with and without the stimulation to learn [22].

The original dataset (more details in Section 6) contains 15 measurements of each protein per sample, for a total of 1080 data points. Control mice learn successfully while the trisomic ones fail, unless they are first treated with memantine, which rescues their learning ability. The dataset is separated into four classes: mice that were not stimulated to learn (*no learning*, 555 samples), control mice that learned (*normal*, 285 samples), not treated and stimulated trisomic mice that failed to learn (*failed*, 105 samples) and treated and stimulated trisomic mice that learned successfully (*rescued*, 135 samples).

We reduced the dimensionality of the data by considering the first 31 principal components (95% of variance kept) and identified 18 clusters using k-means. The resulting ClusterGraph is depicted in Figure 4, alongside the output of popular dimensionality reduction techniques. On most layouts, one can observe two main regions. One is almost entirely composed by *no learning* samples. The second one is dominated by the *normal* group and is also containing the *rescued* and *failed* samples. State of the art techniques such as UMAP and t-SNE are able to better separate the *no learning* samples from the others, but the two embeddings are drastically different. ClusterGraph on the other hand captures the same information and displays in a cleaner, embedding-agnostic way.

3.2.1 Assessing UMAP's layout with ClusterGraph

ClusterGraph can also be applied to the output of any dimensional reduction algorithm, in order to assess the quality of the returned low-dimensional embedding. As already stated, many of these methods aim to preserve the local structure of the point cloud, but they offer no guarantees on the global layout, as demonstrated in the following example.

We focus our attention on the *failed* and *rescued* classes. Variable selection using a random forest method [5] was applied in order to identify the 10 most discriminating variables, this 10-dimensional point cloud was then visualized using UMAP (Figure 5(a)). Such projection is able to separate well the two classes, moreover some points appear to be outliers.

In order to quantify this observation, 10 clusters were selected from the two dimensional embedding using k-means (Figure 5(b)). A ClusterGraph was then build on top of them using the distance between clusters in the original 10-dimensional space. The connectivity pruned ClusterGraph is depicted in Figure 5(c), and it allows to compare the organization of the points in the original space versus the low dimensional UMAP embedding. In both visualizations cluster 9 appears to be the central one, which is consistent with it being composed by a mixture of points from the two classes. We can however spot some clear differences between the two layouts. Cluster 3, which is the outlier on the top left of the UMAP plot, is not an outlier in the ambient space, as it is closer to cluster 9 than, for example, cluster 6, which UMAP places close to the center. Conversely, cluster 2, which appears to be near the center in the UMAP plot, is at a significantly larger distance in the original space.

It is important to notice how both visualizations agree with respect to the *local* layout, the differences appear a larger scales where UMAP fails to capture the global layout of the data.

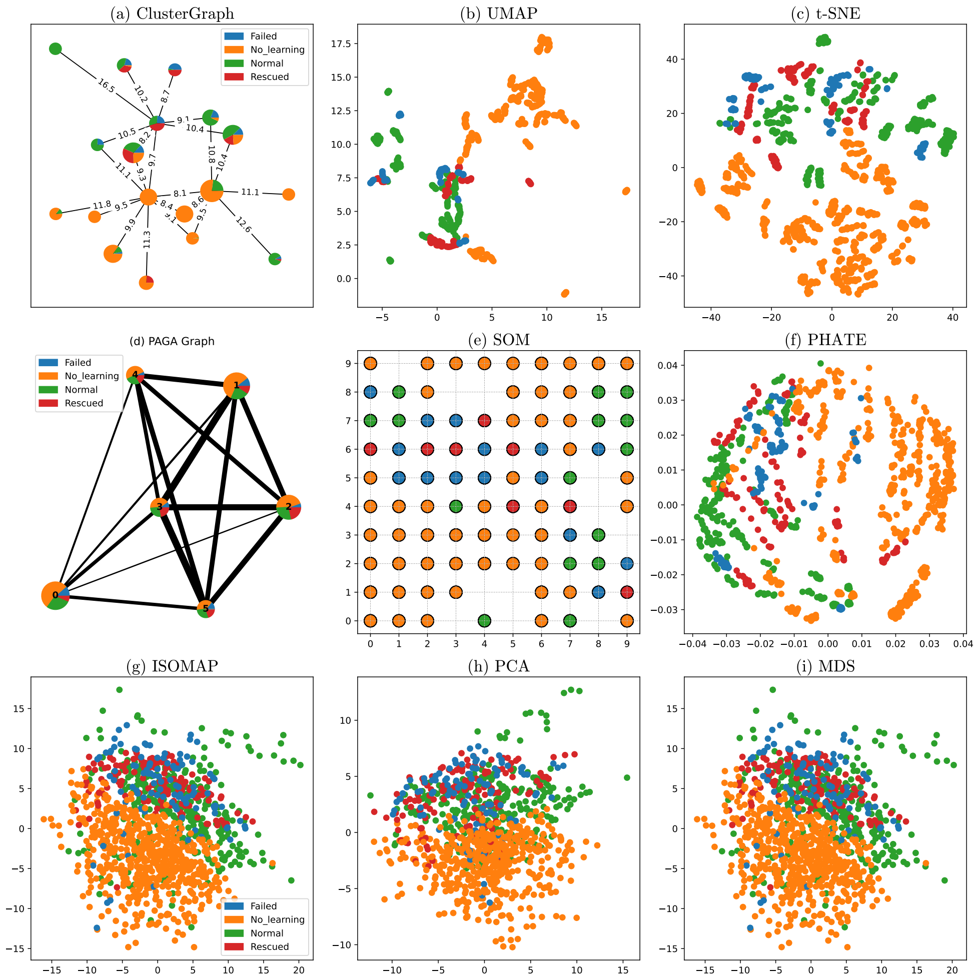

Fig. 4: ClusterGraph visualization of the mice protein expression dataset alongside the output of other dimensionality reduction techniques. Each vertex in the ClusterGraph is depicted as a pie chart whose radius is proportional to the size of the corresponding cluster.

3.2.2 Multi-level ClusterGraph

In some scenarios we might be interested in further clustering a dataset which is already partitioned. This is the case of our working example as each sample belongs to one of four classes: no learning, normal, failed or rescued. A simple ClusterGraph obtained from such a coarse subdivision is depicted in Figure 6(a). Therefore we could cluster samples of each class separately thus obtaining a finer partition that still respect the class labels, i.e. all clusters are monochromatic with respect to the class label. We partitioned the samples of each class into sub clusters: two for rescued and failed, four

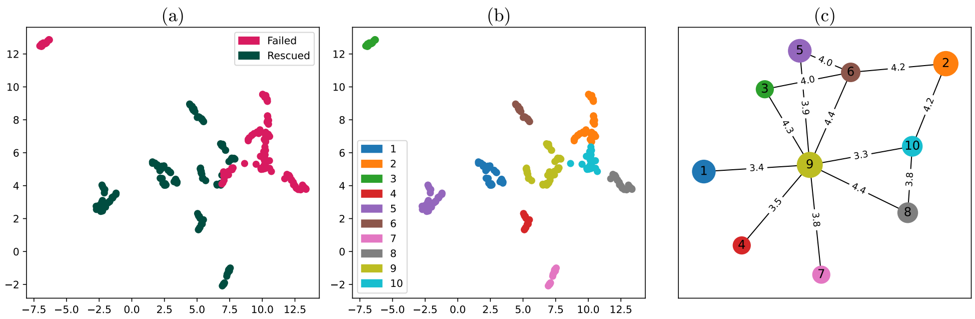

Fig. 5: UMAP visualization of the *failed* and *rescued* samples is depicted in panels (a) and (b). Points are colored by class in the former and by the output of a k-means clustering in the latter. Panel (c) depicts the ClusterGraph obtained from such clustering, where the distances are computed on the original space.

for normal, and five for no-learning by applying k-means to the data projected into the first 31 principal components corresponding to 95% of the variance kept using PCA. The number of sub clusters for each class was chosen based on the class-specific inertia.

A connectivity pruned version of this ClusterGraph is shown in Figure 6(b) by pruning 56 edges. Figure 6(a) shows that the rescued and failed classes are closely related, a relationship further confirmed by Figure 6(b). Although no learning and normal classes appear as the most distant clusters in Figure 6(a), this relationship is more nuanced: it can be explained by the presence of an outlier (cluster 8), while clusters 9 and 7 remain connected to the no learning nodes.

Note that the global layout is coherent with the ClusterGraph built on the full dataset without class labels, as shown in Figure 6(a). This multi-level approach allows us to obtain a clearer visualization by using the class labels as prior knowledge.

3.3 Bone marrow mononuclear cells

The data analyzed in this section consists of bone marrow mononuclear cells of healthy human donors [23] and was part of NeurIPS 2021 OpenProblem benchmarking dataset. The dataset consist of the expression levels of 23427 genes for 17041 samples. After normalization, we identified the top 2000 highly variable genes and computed the first 50 principal components. We then computed the neighborhood graph of cells using the PCA representation of the data matrix and clustered it using the Leiden algorithm [24], separating it into 14 clusters.

Figure 7 depict in panel (a) the two-dimensional UMAP projection of the first 50 principal components, colored by the corresponding cluster. We then computed the ClusterGraph of the first 50 principal components, using as input the 14 clusters found by the Leiden algorithm. A connectivity-pruned ClusterGraph is shown in Figure 7 panel (b), whose edge labels indicate the average distance between two clusters.

We can observe how both visualization techniques agree on the local organization of clusters, e.g. clusters 3, 8, 11 are close together, as well as clusters 5, 9, 13. At the same time, ClusterGraph allows us to gain insights on the global layout that are hidden by

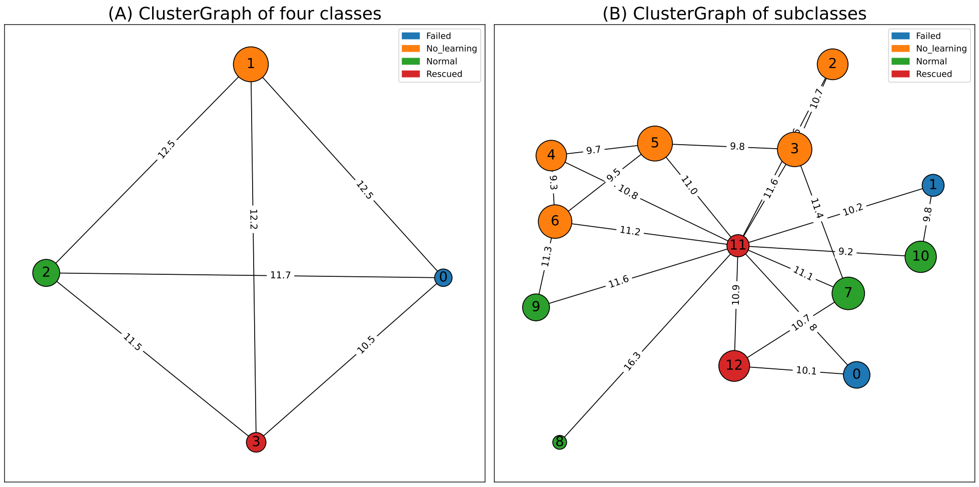

Fig. 6: A simple ClusterGraph where each cluster corresponds to one of the four classes is depicted in panel (a). Each cluster can be further subdivided using k-means(with k equals to two for rescued and failed, four for normal, and five for no-learning classes), the resulting connectivity pruned ClusterGraph is shown in panel (b). The radius of each vertex is proportional to the size of the corresponding cluster.

the two-dimensional constrains of the UMAP plot. For example cluster 6 is actually closer to cluster 0 than cluster 2.

3.4 Human lung cancer cell lines

The last data set [25] consists of single cell RNA-seq of five human lung adenocarcinoma cell lines HCC827, H838, H2228, H1975 and A549. The five lung cancer cell lines were profiled using the 10X Chromium single-cell RNA sequencing platform.

We analyzed the raw count matrix with genes represented as columns. A preprocessing pipeline was applied to retain only the most informative genes, focusing on those with high variability. Principal Component Analysis (PCA) was then performed, and the top 40 principal components were retained for downstream analysis. The resulting dataset was visualized using both UMAP and ClusterGraph, as shown in Figure 8.

In Figure 8, the UMAP embedding reveals five clearly separated clusters, which largely correspond to the five cell lines. The green (H1975) and pink clusters (H838) appear closer together, while the blue (A549) cluster is positioned further away. This organization may suggest that the green (H1975) and pink (H838) cell populations are more similar to each other than either is to the blue one (A549). However, this interpretation can be further validated using ClusterGraph. By comparing cluster 8 (green cells) with cluster 5 (blue cells) and cluster 6 (pink cells), we observe that the distances from cluster 8 to both cluster 5 and cluster 6 are approximately equal. This indicates that the blue (A549) cluster is not an outlier, but is as distinct from

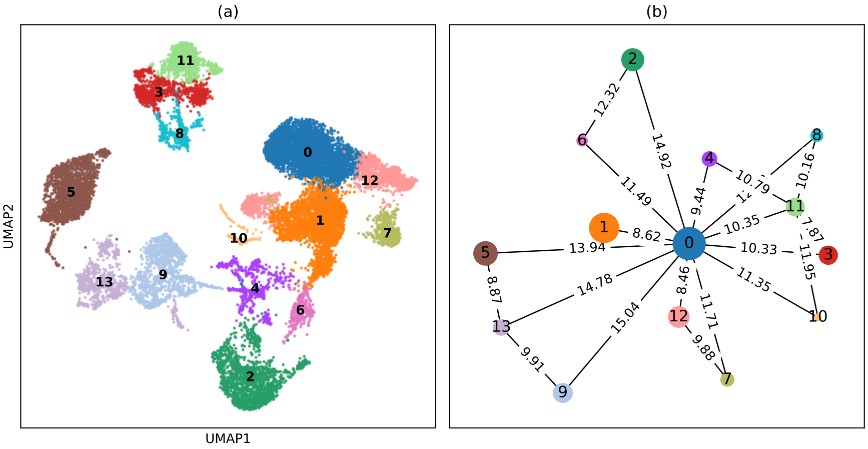

Leiden 0.5

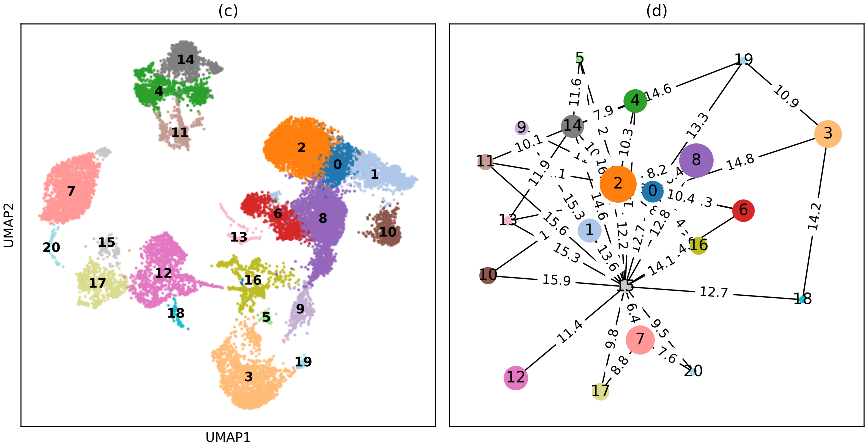

Leiden 0.75

Fig. 7: UMAP plot (a, c) and ClusterGraph (b, d) of the bone marrow single cell dataset for two different Leiden thresholds. The colors corresponds to the clusters found by the Leiden algorithm.

the green (H1975) cluster as the pink (H838) cluster is. ClusterGraph thus provides a more balanced and interpretable view of the relationships between clusters that may not be fully captured in UMAP alone.

Another insight provided by the ClusterGraph is the ability to decompose each group into subgroups using algorithms like KMeans as illustrated in Figure 8 (a). For instance, in the UMAP embedding, the pink (H838) cluster appears more dispersed

compared to the compact purple (H2228) cluster, which might suggest potential sub-populations or varying cellular states within the pink (H838) group. However, when examining the ClusterGraph, the distances between the purple (H2228) sub clusters are actually greater than those within the pink cluster. This highlights how ClusterGraph can offer a more nuanced perspective on intra-cluster relationships that may not be immediately apparent in UMAP plots.

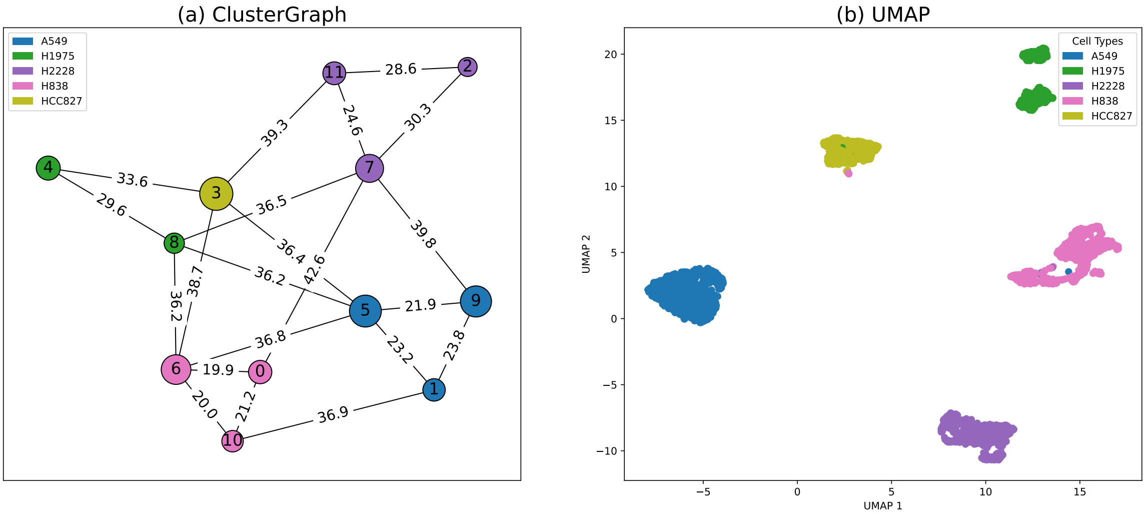

Fig. 8: Visualization of the five cells line dataset using ClusterGraph and UMAP. (a) ClusterGraph representation generated using KMeans clustering with k = 12. Each node represents a cluster and is colored according to its dominant cell population, defined as the label accounting for at least 98% of the cells within the cluster. (b) UMAP projection. The color scheme corresponds to the five cell lines: HCC827, H838, H2228, H1975, and A549.

4 Discussion

ClusterGraph is an effective tool for data visualization and compression. It can be constructed based on any clustering of the input data or arbitrary division of the data into labels. By introducing a notion of distance between clusters it provide a graph-based structure representing relationships within the data. Appropriate intrinsic-distance-preserving pruning of this graph results in a pruned graph that accurately represent the structure of the datasets. The quality of the obtained ClusterGraph can be assessed using the introduced concept of the metric distortion. It allows the analyst to assess the quality of ClusterGraph representation before further investigation. We believe that ClusterGraph can serve as a comprehensive addition to the existing methods of dimensionality reduction and high dimensional data visualization.

5 Source code and requirements

A Python implementation of ClusterGraph is available on the following public github repository https://github.com/dioscuri-tda/ClusterGraph. Examples and tutorials can be found on the github page https://github.com/dioscuri-tda/ClusterGraph/tree/main/examples. The stable version of the package is also available on the Python Package Index https://pypi.org/project/clustergraph and can be installed via the shell command `pip install clustergraph`. The ClusterGraph package uses the following Python libraries:

- Pandas
- Scipy
- Numpy
- Networkx
- Matplotlib
- Scikit-learn
- POT: Python Optimal Transport

More information can be found on the github repository.

6 Data availability

All the real-world datasets used in this article are publicly available and can be found at:

- Mice protein expression data [22] https://doi.org/10.6084/m9.figshare.1421985.v1
- Bone marrow mononuclear cells of healthy human donors [23] https://openproblems.bio/datasets/openproblems_v1/immune_cells
- Single-Cell RNA-seq of five human lung cancer cell lines [25]: https://github.com/LuyiTian/sc_mixology with the GEO accession number GSM3618014.

Acknowledgments. PD, DG and MH acknowledge support by the Dioscuri program initiated by the Max Planck Society, jointly managed with the National Science Centre (Poland), and mutually funded by the Polish Ministry of Science and Higher Education and the German Federal Ministry of Education and Research.

Authors' contributions. PD conceived and directed the project. All the authors collaborated on the mathematical aspects, the design of the algorithm and the writing of the manuscript. DG directed the writing of the manuscript. MH, under the supervision of DG, developed the software, collected and analyzed the experimental data and defined the evaluation metric. AJL helped in writing and revision of the manuscript.

References

[1] Pearson, K.: On lines and planes of closest fit to systems of points in space. The London, Edinburgh, and Dublin Philosophical Magazine and Journal of Science **2**(11), 559–572 (1901) https://doi.org/10.1080/14786440109462720

[2] Maaten, L., Hinton, G.: Visualizing data using t-SNE. Journal of Machine Learning Research **9**, 2579–2605 (2008)

[3] McInnes, L., Healy, J., Melville, J.: UMAP: Uniform Manifold Approximation and Projection for Dimension Reduction. ArXiv e-prints (2018) arXiv:1802.03426 [stat.ML]

[4] Moon, K.R., Van Dijk, D., Wang, Z., Gigante, S., Burkhardt, D.B., Chen, W.S., Yim, K., Van Den Elzen, A., Hirn, M.J., Coifman, R.R., Ivanova, N.B., Wolf, G., Krishnaswamy, S.: Visualizing Structure and Transitions for Biological Data Exploration (2017) https://doi.org/10.1101/120378 . Institution: Bioinformatics Type: preprint. Accessed 2024-01-27

[5] Hastie, T., Friedman, J., Tibshirani, R.: The Elements of Statistical Learning. Springer Series in Statistics. Springer, New York, NY (2001). https://doi.org/10.1007/978-0-387-21606-5 . http://link.springer.com/10.1007/978-0-387-21606-5 Accessed 2023-07-11

[6] Saxena, A., Prasad, M., Gupta, A., Bharill, N., Patel, O.P., Tiwari, A., Er, M.J., Ding, W., Lin, C.-T.: A review of clustering techniques and developments. Neurocomputing **267**, 664–681 (2017) https://doi.org/10.1016/j.neucom.2017.06.053

[7] Morgan, C.L.: Embedding metric spaces in Euclidean space. Journal of Geometry **5**(1), 101–107 (1974) https://doi.org/10.1007/BF01954540 . Accessed 2024-03-06

[8] Bourgain, J.: On lipschitz embedding of finite metric spaces in Hilbert space. Israel Journal of Mathematics **52**(1), 46–52 (1985) https://doi.org/10.1007/BF02776078 . Accessed 2024-03-06

[9] Höppner, F., Klawonn, F., Kruse, R., Runkler, T.: Fuzzy Cluster Analysis. Wiley IBM PC Series. John Wiley & Sons, Chichester, England (1999)

[10] Singh, G., Memoli, F., Carlsson, G.: Topological Methods for the Analysis of High Dimensional Data Sets and 3D Object Recognition. The

Eurographics Association, ??? (2007). https://doi.org/10.2312/SPBG/SPBG07/091-100 . Accepted: 2014-01-29T16:52:11Z ISSN: 1811-7813. https://diglib.eg.org:443/xmlui/handle/10.2312/SPBG.SPBG07.091-100 Accessed 2023-04-27

[11] Dłotko, P.: Ball mapper: a shape summary for topological data analysis. arXiv. arXiv:1901.07410 [math] (2019). https://doi.org/10.48550/arXiv.1901.07410 . http://arxiv.org/abs/1901.07410 Accessed 2023-04-27

[12] Rubner, Y., Tomasi, C., Guibas, L.J.: The Earth Mover's Distance as a Metric for Image Retrieval. International Journal of Computer Vision **40**(2), 99–121 (2000) https://doi.org/10.1023/A:1026543900054 . Accessed 2023-04-28

[13] Wasserstein, L.N.: Markov processes over denumerable products of spaces describing large systems of automata. Problems of Information Transmission **5**(1), 47–52 (1969)

[14] Dłotko, P., Gurnari, D., Sazdanovic, R.: Mapper–type algorithms for complex data and relations. Journal of Computational and Graphical Statistics, 1–14 (2024) https://doi.org/10.1080/10618600.2024.2343321 https://doi.org/10.1080/10618600.2024.2343321

[15] Tenenbaum, J.B., Silva, V.d., Langford, J.C.: A Global Geometric Framework for Nonlinear Dimensionality Reduction. Science **290**(5500), 2319–2323 (2000) https://doi.org/10.1126/science.290.5500.2319 . Publisher: American Association for the Advancement of Science. Accessed 2024-03-27

[16] Klein, J., Zachmann, G.: Point cloud surfaces using geometric proximity graphs. Computers & Graphics **28**(6), 839–850 (2004) https://doi.org/10.1016/j.cag.2004.08.012 . Accessed 2024-03-27

[17] Ruggeri, M.R., Darom, T., Saupe, D., Kiryati, N.: Approximating geodesics on point set surfaces. In: Proceedings of the 3rd Eurographics / IEEE VGTC Conference on Point-Based Graphics. SPBG'06, pp. 85–94. Eurographics Association, Goslar, DEU (2006)

[18] Yu, H., Zhang, J.J., Jiao, Z.: Geodesics on Point Clouds. Mathematical Problems in Engineering **2014**, 860136 (2014) https://doi.org/10.1155/2014/860136 . Publisher: Hindawi. Accessed 2023-04-27

[19] Bernstein, M., Silva, V., Langford, J.C., Tenenbaum, J.B.: Graph approximations to geodesics on embedded manifolds. (2000)

[20] Narasimhan, G., Smid, M.: Geometric Spanner Networks. Cambridge University Press, Cambridge (2007). https://doi.org/10.1017/CBO9780511546884

[21] Zhou, F., Mahler, S., Toivonen, H.: Simplification of Networks by Edge Pruning.

In: Berthold, M.R. (ed.) Bisociative Knowledge Discovery: An Introduction to Concept, Algorithms, Tools, And Applications, pp. 179–198. Springer, Berlin, Heidelberg (2012). https://doi.org/10.1007/978-3-642-31830-6_13

[22] Higuera, C., Gardiner, K.J., Cios, K.J.: Self-Organizing Feature Maps Identify Proteins Critical to Learning in a Mouse Model of Down Syndrome. PLOS ONE **10**(6), 0129126 (2015) https://doi.org/10.1371/journal.pone.0129126 . Publisher: Public Library of Science. Accessed 2024-04-11

[23] Luecken, M.D., Büttner, M., Chaichoompu, K., Danese, A., Interlandi, M., Mueller, M.F., Strobl, D.C., Zappia, L., Dugas, M., Colomé-Tatché, M., Theis, F.J.: Benchmarking atlas-level data integration in single-cell genomics. Nature Methods **19**(1), 41–50 (2022) https://doi.org/10.1038/s41592-021-01336-8

[24] Traag, V.A., Waltman, L., Eck, N.J.: From louvain to leiden: guaranteeing well-connected communities. Scientific Reports **9**(1), 5233 (2019) https://doi.org/10.1038/s41598-019-41695-z

[25] Tian, L., Dong, X., Freytag, S., Lê Cao, K.-A., Su, S., JalalAbadi, A., Amann-Zalcenstein, D., Weber, T.S., Seidi, A., Jabbari, J.S., Naik, S.H., Ritchie, M.E.: Benchmarking single cell RNA-sequencing analysis pipelines using mixture control experiments. Nature Methods **16**(6), 479–487 (2019) https://doi.org/10.1038/s41592-019-0425-8

Personal Cover
Click here to access/download;Personal Cover;Cover_letter_ClusterGraph.pdf

Mathis Hallier

Dioscuri Centre in Topological Data Analysis,
Mathematical Institute, Polish Academy of Sciences,
Sniadeckich 8, Warsaw, Poland

mathis.hallier28@gmail.com

Dear Dr Nogoy and the Editorial Team,

We are writing to resubmit our manuscript titled “**ClusterGraph: a new tool for visualization and compression of multidimensional data**” for your consideration in *GigaScience*. This revised version follows your earlier editorial decision, in which you requested the inclusion of missing sections, additional benchmarking, and other specific enhancements for further consideration.

We are pleased to report that we have thoroughly addressed all points raised in your letter. In particular:

Source Code & Requirements
We have added a dedicated *Source Code & Requirements* section to the manuscript. The full implementation of *ClusterGraph* is now openly available as a public Python library, with clear installation instructions, environment details, example usage, and all scripts necessary to reproduce the figures and experiments in the paper.

Data Availability
We now clearly list all datasets used in the study, emphasizing accessibility and reproducibility. All datasets, including those for benchmarking, are openly available either through public repositories or via the project’s GitHub page. Where applicable, we have provided accession numbers, and the Data Availability section contains all relevant links and metadata.

Benchmarking and Comparison to Existing Tools

We have substantially expanded the manuscript to include a comprehensive benchmarking section. In this section, we compare *ClusterGraph* with eight established visualization tools, including UMAP, t-SNE, and PCA. We evaluate performance across multiple datasets, highlighting key differences and demonstrating how *ClusterGraph* can detect and correct misleading visualizations—particularly with respect to preserving the global data structure.

Single-Cell Data Experiments

In response to your suggestion, we have added two new experiments analyzing publicly available single-cell datasets. These demonstrate the utility of *ClusterGraph* in the context of single-cell data analysis and confirm its ability to provide robust insights into subgroup structure in highly complex, high-dimensional data.

We believe that the manuscript has been improved considerably, both in scientific content and reproducibility, and that it addresses your requests regarding openness, benchmarking, and broader relevance. We kindly submit this revised version for your consideration in *GigaScience*.

We greatly appreciate your time and the constructive feedback, which has helped us enhance our work. We look forward to hearing from you.

Sincerely,

Mathis Hallier
