# Supplementary material for: ClusterGraph: a new tool for visualisation and compression of multidimensional data
Source: Gigascience. 2026 Jun 13;15:giag070. doi: 10.1093/gigascience/giag070 (PMC13354933; doi:10.1093/gigascience/giag070)
Supplement: giag070_GIGA-D-25-00347_revision_1 [file giag070_giga-d-25-00347_revision_1.pdf]

GigaScience

ClusterGraph: a new tool for visualisation and compression of multidimensional data

--Manuscript Draft--

**Manuscript Number:**	GIGA-D-25-00347R1
**Full Title:**	ClusterGraph: a new tool for visualisation and compression of multidimensional data
**Article Type:**	Research
**Funding Information:**	Bundesministerium für Bildung und Forschung — Dr Paweł Dłotko<br>Narodowe Centrum Nauki — Dr Paweł Dłotko
**Abstract:**	Understanding the organisation of high dimensional data is of primary interest for many branches of applied sciences. It is typically achieved by applying dimensionality reduction techniques which, while preserving local features, often miss the global structure of the dataset. Clustering techniques are another class of methods operating in the ambient space, grouping together similar points. However, unlike dimensionality reduction techniques, they do not provide information about organisation of the data. Leveraging ideas from Topological Data Analysis, in this paper we provide an additional layer on the output of any clustering algorithm. Such a data structure, ClusterGraph, provides information about the global layout of clusters, obtained from the chosen clustering algorithm. Appropriate measures are provided to assess the quality and usefulness of the obtained representation. Subsequently the ClusterGraph, possibly with an appropriate structure–preserving simplification, can be visualised and used in synergy with state-of-the-art exploratory data analysis techniques.
**Corresponding Author:**	Mathis Hallier, M.D<br>Instytut Matematyczny Polskiej Akademii Nauk<br>Warsaw, POLAND
**Corresponding Author Secondary Information:**	
**Corresponding Author's Institution:**	Instytut Matematyczny Polskiej Akademii Nauk
**Corresponding Author's Secondary Institution:**	
**First Author:**	Mathis Hallier, M.D
**First Author Secondary Information:**	
**Order of Authors:**	Mathis Hallier, M.D<br>Davide Gurnari, Phd<br>Anna Jurek-Loughrey, Phd<br>Paweł Dłotko, Phd
**Order of Authors Secondary Information:**	
**Response to Reviewers:**	Dear Editor,<br>We would like to express our gratitude to the reviewers for their insightful comments and suggestions, which have significantly contributed to enhancing our manuscript. Below, we provide detailed explanations of how each comment has been addressed. All the changes are marked in green in the manuscript pdf. Reviewer comments are marked between "***Reviewer" for<br>clarity, with our responses immediately following each point.<br><br>Thank you for your assistance in this process.<br>Best wishes,<br>Mathis Hallier (on behalf of all authors)<br><br><br>************* Reviewer

Powered by Editorial Manager® and ProduXion Manager® from Aries Systems Corporation

Reviewer reports:

Reviewer #1: This manuscript introduces ClusterGraph, a graph-based abstraction built on top of clustering results, aiming to capture and visualize the global organization of high-dimensional data while avoiding distortions inherent to low-dimensional embeddings.

1.In realistic high-dimensional biological or social datasets, many assumptions are rarely verifiable. The manuscript would benefit from a discussion of failure modes, robustness to noisy clustering, and sensitivity to the choice of k in k-NN graphs.
************* End Reviewer

We thank the Reviewer for this important comment. We agree that, in realistic high-dimensional biological or social datasets, the assumptions underlying clustering and neighborhood construction are often difficult to verify directly. ClusterGraph is built on top of an input partition of the data into clusters, and therefore its quality necessarily depends on the quality of that partition.
In particular, if the clustering is of poor quality, this can already be detected at the level of the partition itself. When a metric or similarity measure is available, one can assess this indirectly through, for example, large within-cluster diameters. In such a case, points assigned to the same cluster are identified at distance zero in the ClusterGraph representation, despite being far apart in the original space, which leads to a large distortion already at the partition level. Importantly, this acts as a warning signal: rather than silently producing a misleading summary, the method indicates that the chosen clustering should be reconsidered.
To fully address this comment, we have added Section 3.5 “ClusterGraph stability” to our manuscript, where we discuss the sensitivity of our method to the choice of k in k-NN, to the clustering granularity and its robustness to noise.

************* Reviewer
2.The proposed distortion metric requires pairwise shortest-path computations between clusters and averaging over all inter-cluster point pairs. This raises concerns about scalability to large datasets.
************* End Reviewer

We thank the Reviewer for raising this important point. We agree that the distortion measure, in its most direct form, may be computationally demanding on large datasets, since it relies on shortest-path distances in the underlying neighborhood graph together with averaging over inter-cluster point pairs.
We would like to emphasize that this all-to-all distance construction should be viewed primarily as an ideal reference object. In practice, it is expensive for several reasons. First, it requires constructing the k-NN graph itself, which may already be nontrivial in high dimensions. Second, computing shortest-path distances between all relevant pairs is costly, especially when the ambient dimension is high and exact nearest-neighbor search becomes difficult. For this reason, the exact distortion is not intended as the only practical route in large-scale settings, but rather as a quantity that can be approximated or accelerated.
To address these concerns, we have added Section 2.5 “ClusterGraph scalability”, where we discuss several ways to improve scalability. These include the use of approximate k-NN algorithms, dimensionality reduction as a preprocessing step, and replacing full pairwise averaging by simpler cluster representatives such as centroids. We also describe a landmark-based approximation, which in our view provides the most effective speed-up. More generally, most of the required computations can also be parallelized effectively.

************* Reviewer
3.The experimental section relies heavily on qualitative visual comparisons. While illustrative, these examples do not convincingly demonstrate that ClusterGraph improves downstream tasks, e.g., clustering. Some relevant references are:CDC: A Simple Framework for Complex Data Clustering;Structured Graph Learning for Scalable Subspace Clustering: From Single-view to Multi-view.
************* End Reviewer

Powered by Editorial Manager® and ProduXion Manager® from Aries Systems Corporation

We thank the Reviewer for this important comment. We agree that the current experimental section is primarily qualitative. At the same time, we would like to clarify the scope of the method. ClusterGraph is not proposed as a new clustering algorithm, and it is not merely a visualization method in the usual sense. Rather, it is a graph-based abstraction built on top of a given clustering, whose purpose is both to summarize the global organization of the data and to compress the dataset into a more compact structural representation. For this reason, a direct one-to-one comparison with methods such as PCA, t-SNE, or UMAP is inherently difficult: these methods return pointwise low-dimensional embeddings, whereas ClusterGraph produces a compressed graph-level representation of the clustered data.
This is why we have chosen to include in the manuscript, in addition to real-word examples, experiments with artificial datasets for which the underlying metric structure is known (e.g. Fig. 3 concentric circles). This ground truth knowledge allows us to qualitatively assess faithfulness of the ClusterGraph representation. Additional toy examples are provided in our GitHub repository.

************* Reviewer
4.Typographical and grammatical issues are rare but present (e.g., "two bridge this two approaches" in the Introduction).
************* End Reviewer

Thank you for the comment, we have run a spellchecker on the paper.

****

************* Reviewer
Reviewer #2: This manuscript proposes "ClusterGraph," a new tool designed to address the limitations of existing high-dimensional data analysis methods. Current dimensionality reduction techniques (e.g., t-SNE, UMAP), while effective at preserving local features, often lose the global structure of the dataset. Conversely, clustering algorithms partition data but fail to provide information regarding the inter-cluster organization. ClusterGraph bridges this gap by constructing a graph layer on top of the output of any clustering algorithm. In this graph, vertices represent clusters, and edges

Powered by Editorial Manager® and ProduXion Manager® from Aries Systems Corporation

represent the distances in the ambient space. The authors introduce the concept of "Metric Distortion" to assess the consistency between the graph representation and the intrinsic geometric structure of the data. Furthermore, pruning strategies based on metric distortion and connectivity are proposed to optimize visualization. The authors demonstrate the effectiveness of this tool in capturing global structure, data compression, and visualization using synthetic datasets (concentric circles) and real-world biological datasets (mice protein expression, bone marrow mononuclear cells, and lung cancer cell lines). An open-source Python implementation is also provided.

Strengths:

1. Innovation and Complementarity: The method cleverly leverages ideas from Topological Data Analysis (TDA) to provide an intermediate representation between dimensionality reduction and pure clustering. It does not attempt to replace t-SNE or UMAP but offers an "embedding-agnostic" alternative. By encoding distances directly on graph edges rather than forcing a projection into a low-dimensional Euclidean space, ClusterGraph avoids the distance distortions inherent in standard embedding techniques.

2. Rigorous Theoretical Foundation: Unlike many purely heuristic tools, this work is supported by rigorous mathematical definitions and proofs. Specifically, Propositions 1 and 2 provide proofs for the stability of the method under changes in partitions. The use of the k-nearest neighbor graph to estimate intrinsic geodesic distances is well-grounded, citing relevant theorems by Bernstein et al.

3. Objective Quality Assessment: The introduction of "Metric Distortion" is a significant contribution. It allows users to quantitatively evaluate how well the ClusterGraph preserves the intrinsic structure of the data. This metric serves as an objective criterion for automated edge pruning, removing "shortcuts" to better reflect the underlying manifold.

4. Comprehensive and Intuitive Experimental Validation: The synthetic data experiments intuitively demonstrate the limitations of t-SNE and UMAP in preserving global distance ratios, while ClusterGraph captures them accurately. In real-world biological datasets, the authors successfully show how the tool reveals global relationships—such as the outlier status of specific clusters in the lung cancer dataset—that may be obscured or ambiguous in UMAP embeddings.
************* End Reviewer

We sincerely thank the Reviewer for their thorough and generous summary of our work.

************* Reviewer
Weaknesses and Suggestions:

1. Parameter Sensitivity Analysis: The construction of ClusterGraph relies on several parameters, including the choice of the clustering algorithm (e.g., k in k-means or resolution in Leiden), the k value for the k-NN graph used in intrinsic distance estimation, and the pruning thresholds. While different parameter values are used across the experiments, a systematic sensitivity analysis is missing. A discussion on how variations in these parameters—particularly the granularity of the clustering—affect the final graph structure and metric distortion score would be beneficial.
************* End Reviewer

We thank the Reviewer for this important comment. We agree that parameter sensitivity deserves a more systematic discussion. In the revised manuscript, we now address this point in Section 3.5 “ClusterGraph stability”. We include additional quantitative experiments showing how the metric distortion changes with (i) the number of clusters used in the clustering stage, (ii) the neighborhood size k used in the k-NN graph for intrinsic-distance approximation, and (iii) added noise within clusters.

Powered by Editorial Manager® and ProduXion Manager® from Aries Systems Corporation

	*************Reviewer<br>2. Visualization Scalability: While ClusterGraph aims to "compress" data for visualization, the examples provided utilize a relatively small number of clusters (approximately 10-20). For complex datasets requiring a significantly higher number of clusters (e.g., n > 50), the fully connected graph (even after pruning) might become visually cluttered. It would be helpful to discuss the readability and limitations of the visualization when the node count increases.<br>************* End Reviewer<br><br>We thank the Reviewer for this important comment. We agree that readability becomes a genuine issue when the number of nodes increases substantially. The current examples were intentionally kept in the range of approximately 10–20 clusters in order to make the visual message as clear as possible. For substantially larger numbers of clusters, a flat visualization of the full ClusterGraph may indeed become cluttered, even after pruning.<br>At the same time, we would like to emphasize that ClusterGraph should not be viewed only as a static visualization object. It is also a data-compression and structural-abstraction tool. From this perspective, when the number of clusters becomes too large for a single readable view, the natural next step is not necessarily to display all clusters at once, but rather to move to a hierarchical or multiscale representation.<br>We also note that the computational side of graph visualization at these sizes is not the main bottleneck. Modern graph-visualization platforms such as Gephi are specifically designed for interactive exploration of large networks, with millions of nodes and edges.<br><br><br>*************Reviewer<br>3. Computational Complexity: The manuscript would benefit from a discussion on computational costs. Calculating inter-cluster distances (e.g., Wasserstein distance) and the metric distortion for all pairs can be computationally intensive. A brief analysis of the time complexity and scalability of the pipeline on large-scale datasets (e.g., single-cell data with millions of cells) is recommended.<br>*************End Reviewer<br><br>We thank the Reviewer for raising this important point. We agree that the full pipeline may be computationally demanding on large datasets, and we have expanded the discussion of computational aspects in Section 2.5 and in Appendix A.<br>To address scalability, we discuss several practical strategies in the manuscript. These include the use of approximate k-NN algorithms, dimensionality reduction as a preprocessing step, and replacing full pairwise averaging by simpler cluster representatives such as centroids. We also describe a landmark-based approximation, which in our view provides the most effective speed-up. More generally, most of the required computations can also be parallelized effectively.<br><br><br>************* Reviewer
**Additional Information:**	
**Question**	**Response**
Are you submitting this manuscript to a special series or article collection?	No
**Experimental design and statistics**<br><br><br>Full details of the experimental design and statistical methods used should be given in the Methods section, as detailed in our Minimum Standards Reporting Checklist. Information essential to interpreting the	Yes

Powered by Editorial Manager® and ProduXion Manager® from Aries Systems Corporation

data presented should be made available in the figure legends.<br><br>Have you included all the information requested in your manuscript?	
**Resources**<br><br>A description of all resources used, including antibodies, cell lines, animals and software tools, with enough information to allow them to be uniquely identified, should be included in the Methods section. Authors are strongly encouraged to cite Research Resource Identifiers (RRIDs) for antibodies, model organisms and tools, where possible.<br><br>Have you included the information requested as detailed in our Minimum Standards Reporting Checklist?	Yes
**Availability of data and materials**<br><br>All datasets and code on which the conclusions of the paper rely must be either included in your submission or deposited in publicly available repositories (where available and ethically appropriate), referencing such data using a unique identifier in the references and in the "Availability of Data and Materials" section of your manuscript.<br><br>Have you have met the above requirement as detailed in our Minimum Standards Reporting Checklist?	Yes
GigaScience has policies and guidelines in place for the use of generative AI-writing tools such as ChatGPT. If you have used such writing tools to assist with writing the manuscript this must be declared and cited in the text. Authors should not list AI-writing tools and other AI-assisted technologies as an author or co-author and should acknowledge that they are fully responsible for text	No

generated or refined by AI-writing tools.<p><br><br>A summary of use (particularly in the introduction or among methods) needs to be included at the end of the paper, and the outputs should also be included as a supplementary file hosted in GigaDB or other open repositories. Please read our guidelines for more information. <p><br><br>By submitting to GigaScience, you are aware of the journal's AI-writing tools policy, and if you have declared use of such tools below, you have acknowledged this where appropriate in your manuscript and have made a summary of use and outputs available. <p><br><b>AI-assisted writing tools have been used in the preparation of this manuscript?	

Powered by Editorial Manager® and ProduXion Manager® from Aries Systems Corporation

Manuscript Click here to access/download;Manuscript;Cluster_graph.pdf

ClusterGraph: a new tool for visualisation and compression of multidimensional data

Paweł Dłotko[1,2], Davide Gurnari[2*], Mathis Hallier[2,3*], Anna Jurek-Loughrey[4]

[1]Center of Trustworthy AI for Life Sciences – International Research Agendas Programme, Warsaw University, Warsaw, PL.
[2]Dioscuri Centre in Topological Data Analysis, Mathematical Institute, Polish Academy of Sciences, Warsaw, PL.
[3]Génie Informatique, Université de Technologie de Compiègne, Compiègne, FR.
[4]School of Electronics, Electrical Engineering and Computer Science, Queens University of Belfast, Belfast, UK.

*Corresponding author(s). E-mail(s): dgurnari@impan.pl; mathis.hallier28@gmail.com;
Contributing authors: p.dlotko@uw.edu.pl; a.jurek@qub.ac.uk ;

Abstract

Understanding the organisation of high-dimensional data is of primary interest for many branches of applied sciences. It is typically achieved by applying dimensionality reduction techniques which, while preserving local features, often miss the global structure of the dataset. Clustering techniques are another class of methods operating in the ambient space, grouping together similar points. However, unlike dimensionality reduction techniques, they do not provide information about the organisation of the data.
Leveraging ideas from Topological Data Analysis, in this paper we provide an additional layer on the output of any clustering algorithm. Such a data structure, *ClusterGraph*, provides information about the global layout of clusters, obtained from the chosen clustering algorithm. Appropriate measures are provided to assess the quality and usefulness of the obtained representation. Subsequently, the ClusterGraph, possibly with an appropriate structure–preserving simplification, can be visualised and used in synergy with state-of-the-art exploratory data analysis techniques.

1 Introduction

High-throughput experiments are becoming extremely common in applied sciences. Now more than ever, large high-dimensional datasets are generated in almost every laboratory, calling for an automated and reliable way to extract new knowledge from them. Let us fix a dataset X, usually embedded in a high-dimensional space. Standard dimension reduction techniques, including PCA [1], t-SNE [2], UMAP [3] and PHATE [4] aim to find a low-dimensional embedding of X so that points that are close in X, are also close in the embedding. However, preservation of the global organisation of X in general, and information about distances of distant points in particular, is a challenge for these methods.

Clustering techniques [5, 6], on the other hand, based on a fixed similarity measure, provide a partition of the input dataset X. However, clustering itself does not provide information about either intra- or inter- cluster organisation of points and is therefore not used to assess the global structure of the data.

In computational biology, both dimensionality reduction and clustering have become essential tools for the analysis of omics data. On the visualisation side, dedicated tools have been developed to cope with the growing size and intricacy of single-cell data, either by providing interactive exploration of hierarchical cell populations [7] or by extending classical dimensionality reduction methods to integrate spatial and molecular information jointly [8]. On the clustering side, efforts have focused both on automating method selection for transcriptomic data [9] and on recovering biologically meaningful hierarchical structure that flat partitions fail to capture [10, 11].

The aim of this work is to bridge these two approaches by enriching the output of a clustering algorithm with additional information on the data's global organisation.

The first contribution of this paper is the construction of a *ClusterGraph*: a graph-based structure on top of a partition $\mathcal{C}(X)$ of the data obtained from a clustering algorithm $\mathcal{C}$ applied to X. In the ClusterGraph $G = (V, E)$, each vertex corresponds to a single cluster from $\mathcal{C}(X)$. Two vertices $u, v \in V$ are connected by an edge whose length corresponds to the distance between their respective clusters in $\mathcal{C}(X)$. For the purpose of this construction, a number of inter-cluster distances defined in the ambient space are used.

The ClusterGraph has a number of advantages compared to alternative dimension reduction methods. One of them is based on the fact that the distances, computed in the ambient space, are represented by labels on edges and not subject to distortions made by standard dimension reduction techniques that force the projected data points to be embedded in a Euclidean space. This allows us to visualise the *global* distances in the dataset. This is important as many datasets cannot be embedded into low-dimensional Euclidean spaces without perturbing the distances between points.

As an example of such a situation consider a collection of points in four clusters: 0, 1, 2 and 3. Points in each cluster are infinitesimally close. The distance between cluster 0 and clusters 1, 2 and 3 is 1, while the distance between clusters 1, 2 and 3 is 2. It is well known [12, 13] that such a graph cannot be isometrically embedded to any Euclidean space $\mathbb{R}^n$ for any n. As a consequence, all dimensionality reduction

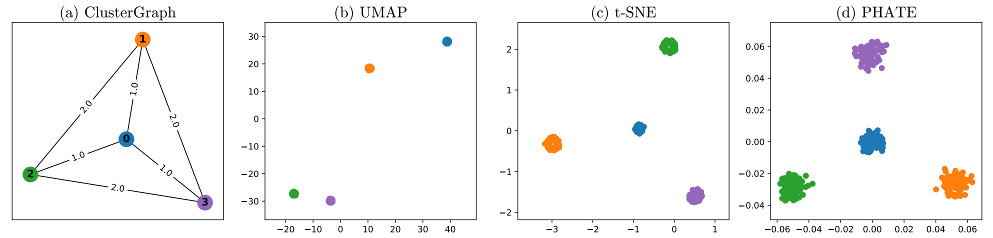

Fig. 1: The dataset consists of four clusters 0 (blue), 1 (orange), 2 (green) and 3 (purple), as described in the text, so that elements of cluster 0 are at distance one from elements from the remaining clusters and the mutual distances between elements of clusters 1, 2 and 3 are equal to two. Such a dataset cannot be embedded, with distances preserved, into any Euclidean space. In this case, UMAP (panel b) fails to capture the global layout, while t-SNE (panel c) and PHATE (panel d) do. However, the coordinate systems of t-SNE and PHATE are drastically different. In both cases, as a result of the embedding into the Euclidean plane, the ratio of the distances $d(1,2)/d(0,1)$ is roughly $\sqrt{3}$ instead of the original 2, the same is true for the other clusters. This is the optimal embedding that can be achieved when points are projected to Euclidean space. However, in the case of ClusterGraph (panel a), the distances are encoded as labels on the graph edges and therefore we are not restricted by any Euclidean space.

techniques will distort the distances between clusters, as can be observed in Fig 1. The ClusterGraph, on the contrary, provides the correct graph even in this case.

The second contribution is a method to assess the quality of the ClusterGraph G. Working under the assumption that X is sampled from a manifold equipped with an intrinsic distance, a *metric distortion* between the intrinsic distances on X and the distance induced by G on X is used to assess the quality of G. The distance between points $x, y \in X$ induced by G is the length of the shortest path in G between vertices representing the clusters containing them.

The logarithm of the ratio between the intrinsic and the ClusterGraph distance is used as a quality measure: the smaller its value, the better the quality of the ClusterGraph representation.

This procedure will be used to obtain a *pruned ClusterGraph* that better approximates the intrinsic structure of the data. For this purpose, a number of *edge pruning* algorithms are proposed, aiming to remove some edges while maintaining the global structure of the data. A schematic of the whole ClusterGraph pipeline is depicted in Figure 2.

A Python implementation of the creation of the ClusterGraph data structure, as well as the pruning algorithms and interactive visualisation utilities is available at github.com/dioscuri-tda/ClusterGraph.

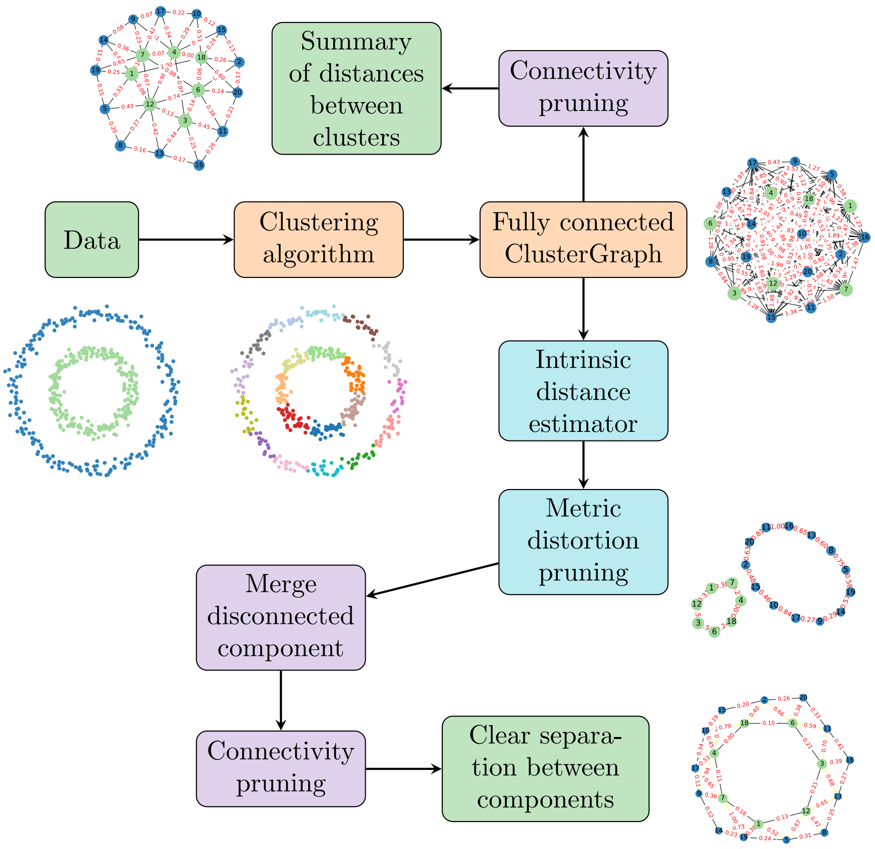

Fig. 2: ClusterGraph pipeline with the two possible pruning strategies. Details on the example dataset used to generate the figures can be found in Section 3.1.

2 Methods

2.1 ClusterGraph

Let X be a dataset equipped with a metric $d_X : X \times X \to \mathbb{R}_{\geq 0}$. Take $\mathcal{C}$ to be an arbitrary hard or soft clustering algorithm. Let $\mathcal{C}(X)$ be the partition or, in a more general case, a division of X obtained from the clustering algorithm $\mathcal{C}$.

A collection of sets $\{C_i\}$ is a *partition* of X if for every $C_i \neq C_j \in \mathcal{C}(X)$, $C_i \cap C_j = \emptyset$ and $\bigcup_{C_i \in \mathcal{C}(X)} C_i = X$. Partitions of X are typically obtained from hard clustering algorithms [5]. In a more general case, we can drop the empty intersection condition turning $\mathcal{C}(X)$ into a *division* of X. Divisions may be obtained using soft or fuzzy clustering methods [14]. They can also be obtained as by-products of Topological Data Analysis techniques like Mapper [15] or Ball Mapper [16].

In the next step we extend d_X to a distance or similarity measure $d_{\mathcal{C}} : \mathcal{C}(X) \times \mathcal{C}(X) \to \mathbb{R}_{\geq 0}$ defined on elements of $\mathcal{C}(X)$, as detailed in Section 2.1.1. The *ClusterGraph* of a partition $\mathcal{C}(X)$ is a fully connected graph whose vertices are elements of $\mathcal{C}(X)$ and edges are weighted by the distance $d_{\mathcal{C}}$ between the elements of $\mathcal{C}(X)$.

ClusterGraph is intended to serve as a tool for data visualisation and compression. Compression is achieved by collapsing points within the same cluster into a vertex of the ClusterGraph. This step is motivated by an assumption that points within the same cluster are in close proximity, hence share multiple characteristics and can be represented by a single vertex. We expect the ClusterGraph to have much fewer vertices compared to the original number of data points. The visualisation aspect is accomplished when the layout of the ClusterGraph resembles, to some extent, the layout of the input point cloud. However, this is unlikely to be the case for a fully connected graph. Therefore, in Section 2.2, we propose a *metric distortion*-based criterion and techniques for removing certain edges of the ClusterGraph so that the metrics on the graph and on the initial point cloud become comparable. Different strategies for edge removal from the ClusterGraph are discussed in Section 2.3. This process yields a *pruned* ClusterGraph, which we use for visualisation purposes.

2.1.1 Distances between clusters

In this section, for a dataset X equipped with a similarity measure d_X and a partition $\mathcal{C}(X)$, we present a number of similarity measures $d_{\mathcal{C}} : \mathcal{C}(X) \times \mathcal{C}(X) \to \mathbb{R}_{\geq 0}$. The choice of the optimal one is application-dependent, very much like the clustering algorithm $\mathcal{C}$, and should therefore be selected and optimised by the user. Given two clusters C_i and C_j, possible options include:

1. Maximum, minimum or average distance between points

$$\begin{aligned} \min(C_i, C_j) &= \min_{x \in C_i, y \in C_j} d_X(x, y) \\ \max(C_i, C_j) &= \max_{x \in C_i, y \in C_j} d_X(x, y) \\ \text{avg}(C_i, C_j) &= \sum_{x \in C_i} \sum_{y \in C_j} d_X(x, y) / (|C_i||C_j|). \end{aligned}$$

2. Hausdorff distance

$$d_H(C_i, C_j) = \max \left\{ \sup_{x \in C_i} d(x, C_j), \sup_{y \in C_j} d(C_i, y) \right\},$$

where $d(a, B) = \inf_{b \in B} d(a, b)$.

3. Earth mover's (a.k.a. Wasserstein) distance [17, 18] which utilises ideas from probability theory and optimal transport

$$W_p(C_i, C_j) = \inf_{\eta: C_i \to C_j} \left(\sum_{x \in C_i} d_X(x, \eta(x))^p \right)^{\frac{1}{p}} ,$$

where η is a matching between points of C_i and C_j and $1 \leq p < \infty$. If the two clusters have different size, the matching is computed in a weighted way, i.e. each point in C_i is assigned a weight of $1/|C_i|$ such that each cluster has a total mass of 1.

2.1.2 Stability

Let us consider a dataset X and two partitions of it $\mathcal{C}(X)$, $\mathcal{D}(X)$ obtained via some clustering algorithms. We are interested in quantifying how different these two partitions can be. In order to do so, we introduce the following concept.

Definition 1 (Image of a cluster) Let X be a dataset and $\mathcal{C}(X)$ and $\mathcal{D}(X)$ two partitions of it. The *image* of a cluster $C_i \in \mathcal{C}(X)$ in $\mathcal{D}(X)$ is the union of all clusters of $\mathcal{D}(X)$ that contain some points of C_i, namely $\mathrm{im}_{\mathcal{D}(X)}(C_i) = \{\bigcup D_j \in \mathcal{D}(X) \mid C_i \cap D_j \neq \emptyset\}$.

This idea of mapping the points covered by one cluster in a given partition to the clusters in a second partition is inspired by an analogous technique for mapper graphs, *MappingMappers*, described in [19].

Let us define the *diameter* of a collection of points as the greatest distance between any pair of points. We can then state the following bound.

Proposition 1 (Clustering stability) *Let X be a dataset and $\mathcal{C}(X)$, $\mathcal{D}(X)$ be two partitions of it such that the diameter of each set in $\mathcal{C}(X)$ and $\mathcal{D}(X)$ is at most δ. Then, for any cluster $C_i \in \mathcal{C}(X)$, its image in $\mathcal{D}(X)$ has diameter at most 3δ.*

Proof Let $d_1, d_2 \in X$ be the two points whose distance realises the diameter of $\mathrm{im}(C_i)$. By definition of image there exists at least one point $c_1 \in C_i$ which lies in the same cluster of $\mathcal{D}(X)$ as d_1, and similarly there exists at least one c_2 for d_2. Therefore, we have

$$d_X(d_1, d_2) \leq d_X(d_1, c_1) + d_X(c_1, c_2) + d_X(c_2, d_2) \leq 3\delta .$$

□

Using a summary statistic of the distance between points as the distance between clusters (option 1 in Section 2.1.1) allows us to derive a similar bound for ClusterGraphs built on top of $\mathcal{C}(X)$ and $\mathcal{D}(X)$.

Definition 2 (Image of a ClusterGraph vertex) Let X be a dataset, $\mathcal{C}(X)$ and $\mathcal{D}(X)$ two partitions of it and $G_{\mathcal{C}(X)}$, $G_{\mathcal{D}(X)}$ the ClusterGraphs obtained from $\mathcal{C}(X)$ and $\mathcal{D}(X)$. The

image of a vertex $i \in G_{\mathcal{C}(X)}$ (corresponding to cluster $C_i \in G_{\mathcal{C}(X)}$) in $G_{\mathcal{D}(X)}$ is the collection of all vertices of $G_{\mathcal{D}(X)}$ that correspond to clusters in $G_{\mathcal{D}(X)}$ containing some points of C_i.

We define the *diameter* of a weighted graph as the greatest distance between any pair of vertices.

Proposition 2 (ClusterGraph stability) *Let X be a dataset, $\mathcal{C}(X)$ and $\mathcal{D}(X)$ two partitions of it and $G_{\mathcal{C}(X)}$ and $G_{\mathcal{D}(X)}$ the ClusterGraphs obtained from them. Assume that the diameter of each set in $\mathcal{C}(X)$ and $\mathcal{D}(X)$ is at most δ. Then the image of each vertex $u \in G_{\mathcal{C}(X)}$ in $G_{\mathcal{D}(X)}$ is a clique of diameter at most 3δ for the maximum and average distance, and δ for the minimum.*

Proof Let u be a vertex in $G_{\mathcal{C}(X)}$ and $\mathrm{im}(u)$ its image in $G_{\mathcal{D}(X)}$.

Recall that $\mathrm{im}(u)$ is a subset of the complete graph $G_{\mathcal{D}(X)}$, therefore, it is a clique. Let D_i and D_j be the clusters in $\mathrm{im}(u)$ whose distance realises the diameter of $\mathrm{im}(u)$, i.e. they correspond to the two vertices in the clique that are furthest apart.

Let us start with the maximum distance case. In particular, let $d_1 \in D_i$ and $d_2 \in D_j$ be the two data points realizing the maximum distance between D_i and D_j, and therefore $d(d_1, d_2) = \mathrm{im}(u)$. Let C_u be the cluster in $\mathcal{C}(X)$ corresponding to vertex $u \in G_{\mathcal{G}(X)}$. By definition of the image of u, there are at least two points $c_1, c_2 \in C_u$ which lie in the same clusters of $\mathcal{D}(X)$ as d_1 and d_2, respectively. We can then proceed in a similar fashion to the proof of Proposition 1, namely we have

$$\mathrm{diam}(\mathrm{im}(u)) = \max(D_i, D_j) = d_X(d_1, d_2) \leq d_X(d_1, c_1) + d_X(c_1, c_2) + d_X(c_2, d_2) \leq 3\delta\ .$$

The same bound holds for the average distance since $\mathrm{avg}(D_i, D_j) \leq \max(D_i, D_j)$.

For the minimum distance case it is sufficient to notice that $d_X(c_1, c_2) \leq \delta$ because they both belong to the same cluster C_i whose diameter is bounded by δ. Hence we have

$$\mathrm{diam}(\mathrm{im}(u)) = \min(D_i, D_j) \leq d_X(c_1, c_2) \leq \delta.$$

□

2.2 Metric distortion

Given a dataset X, different choices of the clustering algorithm $\mathcal{C}$, as well as the metrics d_X and $d_{\mathcal{C}}$, can lead to significantly different ClusterGraphs. The aim of this section is to introduce a score to assess the quality of a given ClusterGraph G by comparing it to the underlying geometric structure of the dataset X.

To this end, let us assume that the point cloud X is sampled from a compact and connected manifold $\mathcal{M}$ equipped with an *intrinsic distance* $d_{\mathcal{M}}$. Informally, the intrinsic distance between two points $x, y \in \mathcal{M}$ is defined as the infimum of the length of a curve $\gamma \subset \mathcal{M}$ joining x and y; this is also known as *geodesic* distance.

In most applications, the underlying manifold is not known. Consequently, the intrinsic distance needs to be estimated from the point cloud. This is a well-studied problem in computational geometry and computer graphics, and multiple methods have been proposed [20–23].

Below, we follow the approach of [20, 24] using the shortest path in the k-nearest neighbour graph as estimator. Note that any other estimator of intrinsic distance can also be used in the proposed construction.

Let $G_{knn}(X)$ be the k-nearest neighbour graph on X constructed as follows: each point of X corresponds to a vertex of $G_{knn}(X)$; it is connected to its k-nearest neighbours (in the chosen distance d_X, typically Euclidean), with k being a parameter of the method. Weights corresponding to the distance between endpoints are assigned to the edges of $G_{knn}(X)$. We define a distance d_X^k on $G_{knn}(X)$, estimating the intrinsic distance on X, as

$$d_X^k(x,y) = \text{the length of the shortest path between } x \text{ and } y \text{ in } G_{knn}(X) \tag{1}$$

Remark 1. It may happen that $G_{knn}(X)$ is not connected. There are two possible reason for this. In the first case points are indeed sampled from a compact and connected manifold but the parameter k is too low. This can be easily solved by increasing k. In the second case the underlying manifold is not connected. This will result in the k-nn graph being disconnected even for very high values of k, especially if many points are sampled. In this case, we will threat each connected component separately, splitting the input dataset X (and the output of the clustering algorithm) into disjoint sets, each one corresponding to a different connected component and analyse each of them separately[1]. For the rest of the Section we therefore assume, without lack of generality, that the k-nn graph is fully connected. We discuss how to investigate the relations between different connected components in Section 2.3.3.

Remark 2. Whenever an estimator is used, it is natural to ask how good such an estimator is. The choice of a k-nn graph as an estimator of the geodesic distance is motivated by the following theorem by Bernstein, Vin de Silva, Langford and Tenenbaum.

Theorem 3 (Theorem A in [24]) *Let $\mathcal{M}$ be a compact submanifold of $\mathbb{R}^n$, X a finite set of data points in $\mathcal{M}$ and G a graph on X (for example, a k-nn graph). Then the inequalities*

$$(1-\lambda_1)d_{\mathcal{M}}(x,y) \leq d_G(x,y) \leq (1+\lambda_2)d_{\mathcal{M}}(x,y)$$

are valid for all x, y in X, where $\lambda_1, \lambda_2 < 1$ are two positive real numbers that depend on G, $\mathcal{M}$ and some technical assumptions on the density of X.

For each point $x \in X$, we denote by C_x the cluster in $\mathcal{C}(X)$ that contains x. We can then use the distance between clusters described in Section 2.1.1 to define a distance between points d_{CG} in the ClusterGraph G as follows

$$d_{CG}(x,y) = \text{the length of the shortest path between } C_x \text{ and } C_y \text{ in } G. \tag{2}$$

[1]By performing the construction in Sec. 2.1 we obtain a ClusterGraph for each connected component, each of them being a fully connected graph. In graph theory such a disjoint union of complete graphs is sometimes called a "cluster graph". This unexpected but pleasing agreement in nomenclature motivates our choice of referring to our construction in camel case, to avoid confusion.

Recall that G is fully connected; one might wonder why the length of the shortest path between two vertices is used instead of the weight of the edge connecting them given by $d_{\mathcal{C}}$. First, the triangle inequality might not hold for $d_{\mathcal{C}}$. Second, we want the definition of d_{CG} to also hold in the case of a pruned ClusterGraph, which we will discuss in Section 2.3.

This notion of d_{CG} is well defined only when $\mathcal{C}(X)$ is a partition. In the more general case of a division, when a point can belong to more than one cluster, we take $d_{CG}(x, y)$ to be the length of the shortest path between any cluster containing x and any cluster containing y.

Consider the ClusterGraph $G = (V, E)$ and let us fix two vertices, $i, j \in V$, corresponding to two clusters C_i and C_j. For a pair of points $x \in C_i$ and $y \in C_j$, $x \neq y$, we compute:

$$\delta(x, y) = |\log\left(\frac{d_{CG}(x, y)}{d_X^k(x, y)}\right)|. \tag{3}$$

The use of the absolute value of the logarithm ensures that a multiplicative scaling by a factor $\lambda = \frac{d_{CG}(x,y)}{d_X^k(x,y)}$ results in the same metric distortion as a $1/\lambda$ scaling, i.e. $|\log(x/y)| = |\log(y/x)|$ for every $x, y \in \mathbb{R}_{>0}$.

By averaging this quantity over all possible pairs of points $x \in C_i$ and $y \in C_j$, we obtain the metric distortion between clusters C_i and C_j.

$$\delta_{\{i,j\}} = \frac{1}{|C_i||C_j|} \sum_{(x,y)\in(C_i,C_j)} \delta(x, y) \tag{4}$$

This score assesses how much the intrinsic distance between points on X differs from their corresponding distance in the ClusterGraph.

The global metric distortion of the ClusterGraph can be obtained by averaging the score for each pair of vertices defined in Equation 4. However, because clusters can have different sizes, we consider a weighted average. The weight of the pair $\{i, j\}$ corresponding to clusters C_i and C_j is defined as:

$$w_{\{i,j\}} = \frac{|C_i \cup C_j|}{(n-1)|X|}, \tag{5}$$

where n denotes the number of clusters in $\mathcal{C}(X)$ (or equivalently, $n = |V|$, the number of vertices in the ClusterGraph). The rationale behind this averaging is to give more importance to interactions between large clusters.

The global metric distortion for a ClusterGraph (with respect to the k-nearest neighbours graph) can then be defined as

$$\Delta_k(G) = \frac{2}{n(n-1)} \sum_{\{i,j\}\in V} w_{\{i,j\}}\delta_{\{i,j\}}. \tag{6}$$

This quantity is a non-negative real number indicating how well the given ClusterGraph respects the intrinsic metric structure of the data. It allows us to compare the

quality of ClusterGraphs having an equal or very similar number of vertices. Comparison of metric distortions of ClusterGraphs having vastly different numbers of nodes should not be performed.

In the next sections we will use each edge's distortion as well as the global distortion to prune the ClusterGraph, with the goal of removing the edges that do not reflect the underlying structure of the data. In practice, computing the metric distortion can be computationally intensive, particularly during pruning. In our current implementation, the following approximation is used instead.

We first compute the average intrinsic distance between clusters:

$$d_{CG}^{\text{geod}}(C_i, C_j) = \frac{1}{|C_i||C_j|} \sum_{x \in C_i} \sum_{y \in C_j} d_X^k(x, y). \tag{7}$$

Comparing this quantity with the ClusterGraph edge weight yields the approximate metric distortion:

$$\widetilde{\Delta_k}(G) = \frac{2}{n(n-1)} \sum_{\{i,j\} \in V} w_{\{i,j\}} \left| \log\left(\frac{d_{CG}(C_i, C_j)}{d_{CG}^{\text{geod}}(C_i, C_j)} \right) \right|. \tag{8}$$

where $d_{CG}(C_i, C_j)$ denotes the shortest path between clusters C_i and C_j in the ClusterGraph.

This approximation is particularly useful when the metric distortion must be evaluated multiple times, such as during the pruning procedure.

Remark 3. The ratio between two distances in our definition of the distortion (Eqn. 3) might be reminiscent of the *stretch factor* or *distortion* of an embedding f. For two given points x and y in a metric space the stretch factor is defined as $d(f(x), f(y))/d(x, y)$. For example, consider a set of points in $\mathbb{R}^d$ and a connected graph having those points as vertex set. Each edge in the graph has a weight corresponding to the Euclidean distance between its endpoints. The stretch factor for two given points is the ratio of the length of the shortest path between them in the graph to their Euclidean distance. The stretch factor of the graph is the maximum stretch factor over any pair of points. Graphs with stretch factor at most t are called *t-spanners* [25].

It is important to point out the differences between this widely studied topic in graph theory and our approach. First, we are not dealing with an embedding as the map that sends each data point to its cluster is highly non-injective. Moreover, the stretch factor of a graph defined in the paragraph above is always greater than or equal to 1. In our setting, the ratio between the ClusterGraph distance and the intrinsic one (Eqn. 3) might be less than 1; this is exactly the case of a "shortcut" edge in the ClusterGraph.

2.3 ClusterGraph pruning

The ClusterGraph is, by definition, a fully connected graph. Consequently, it may contain edges connecting regions of the dataset that are not close in the manifold $\mathcal{M}$

from which the data points of X are sampled. We will refer to these edges as "short-cuts", as they are shorter than the true geodesic distance between the corresponding points in the manifold and therefore are not representative of the underlying manifold structure. The removal of such edges will make the ClusterGraph more similar, in the sense of metric distortion, to X. Moreover, the ClusterGraph may also contain edges whose removal does not considerably change the metric structure of the graph. In this section we introduce three approaches to pruning edges of a given ClusterGraph, and by doing so, of increasing the quality of the obtained representation.

2.3.1 Threshold pruning

If the triangle inequality holds for the distance between clusters $d_{\mathcal{C}}$, the length of the shortest path between each pair of vertices in the ClusterGraph (Eqn. 2) is exactly the length of the edge connecting them. It then makes sense to assign to each edge in the ClusterGraph the metric distortion for its two corresponding clusters, as defined in Equation 4.

The ClusterGraph can then be naively pruned by removing all edges having metric distortion greater than a threshold $\alpha > 0$.

2.3.2 Iterative greedy pruning

Consider the ClusterGraph $G = (V, E)$. Let $\Delta_k(G)$ be its metric distortion as in Equation 6. Denote by $G_{\hat{e}}$ the ClusterGraph obtained by removing edge e from G, namely $G_{\hat{e}} = (V, E \setminus \{e\})$.

We can then perform the following iterative greedy pruning procedure. Remove edge e if both conditions hold:

1. $\Delta_k(G_{\hat{e}}) \leq \Delta_k(G)$,
2. $\Delta_k(G_{\hat{e}}) \leq \Delta_k(G_{\hat{e}'})$ for any other $e' \in E$.

Then update E to $E \setminus \{e\}$. The process may be repeated a fixed number of times, or until no such edge e can be found. Note that the length of the shortest path between two vertices defined in Equation 2 will be infinite if the ClusterGraph becomes disconnected, thereby leading to an infinite value of the metric distortion. Therefore condition (1) ensures that the pruning procedure will never produce new connected components in the ClusterGraph.

2.3.3 Connectivity-based pruning

The first two pruning techniques presented in Sections 2.3.1 and 2.3.2 focus on the removal of the edges with high metric distortion or, informally speaking, the removal of all the "shortcuts" with respect to the structure of X. It might happen that after this pruning the obtained ClusterGraph still has a complicated structure which may render its visualisation and interpretation challenging.

In what follows we adopt the *connectivity based* approach by Zhou, Mahler and Toivonen [26] to the ClusterGraph pruning. A *path* P in $G = (V, E)$ is a set of edges $P = \{\{i_1, i_2\}, \{i_2, i_3\}, \ldots, \{i_{k-1}, i_k\}\} \in E$. A *path quality function* $q(P) \to \mathbb{R}^+$ is defined by taking the sum of the inverse of the path length, calculated using the

distance between clusters $d_{\mathcal{C}}$

$$q(P) = \sum_{\{i,j\}\in P} \frac{1}{d_{\mathcal{C}}(C_i, C_j)}. \tag{9}$$

The *connectivity* between two vertices i, j in $G = (V, E)$ is the quality of the best path between them

$$\mathrm{conn}(i, j; E) = \begin{cases} \max_{P\in\mathcal{P}(i,j)} q(P) & \text{if } \mathcal{P}(i,j) \neq \emptyset \\ -\infty & \text{otherwise} \end{cases} \tag{10}$$

where we denote by $\mathcal{P}(i, j)$ the set of all possible paths between vertices i and j. The *connectivity of a ClusterGraph* is the average connectivity over all pairs of vertices

$$\mathrm{conn}(V, E) = \frac{2}{n(n-1)} \sum_{i,j\in V, i\neq j} \mathrm{conn}(i, j; E), \tag{11}$$

where n is the number of vertices in the ClusterGraph. Note that the connectivity will be $-\infty$ if G is disconnected. In that case each connected component should be analysed separately.

Let us now consider the ClusterGraph with one edge removed $G_{\hat{e}} = (V, E \setminus \{e\})$. It is straightforward to see that $\mathrm{conn}(V, E \setminus \{e\}) \leq \mathrm{conn}(V, E)$. In particular $\mathrm{conn}(V, E \setminus \{e\}) = \mathrm{conn}(V, E)$ if and only if e does not belong to any of the best paths between any pairs of vertices. Moreover, $\mathrm{conn}(V, E\setminus\{e\}) = -\infty$ if the removal of e disconnects the graph. We can then define the *ratio of connectivity kept* after removing an edge or, more generally, after removing a subset of edges $E_R \subset E$

$$rk(V, E, E_R) = \frac{\mathrm{conn}(V, E \setminus E_R)}{\mathrm{conn}(V, E)}. \tag{12}$$

Pruning can then be executed in a greedy iterative fashion (see Alg. 2 BF in [26]) by selecting, at each iteration, the edge whose removal will result in smallest decrease of connectivity, i.e. the largest rk value.

2.4 Merging

As discussed in Remark 1, it may happen that the underlying manifold from which the data are sampled is disconnected. In that case, the resulting ClusterGraph will have more than one connected component and each of them will be pruned separately. In order to capture the global layout of the data, including the disconnected components of the manifold, we may merge different components by adding a collection of special edges between each vertex v and its k-nearest neighbours not belonging to the same connected component as v. Subsequently, the connectivity-based pruning procedure can be applied to the newly added edges.

2.5 ClusterGraph scalability

At scale, computing the ClusterGraph, evaluating the metric distortion, and performing the iterative pruning can become computationally demanding. A detailed complexity analysis is provided in Appendix A. Several strategies can keep the ClusterGraph tractable on large datasets. Exact k-NN graph construction can be replaced with an approximate method such as HNSW [27], reducing the complexity from $O(|X|^2 d)$ to $O(|X| \log |X|)$. Furthermore, performing PCA prior to graph construction reduces the cost of each distance computation. Similarly, using cluster centroids for all pairwise point distances reduces the inter-cluster cost from $O(|X|^2)$ to $O(|X|d + n^2 d)$. Finally, inter-cluster distance computations and single-source Dijkstra runs are mutually independent and can be executed in parallel across available workers. Beyond these, we introduce two more strategies that significantly extend the scalability of ClusterGraph to larger datasets.

2.5.1 k'-NN ClusterGraph approximation

In practice, pruning preserves only connections to nearest neighbour clusters when the data exhibit geometric locality. Consequently, assessing metric distortion on long-range edges is often of limited value, as these edges are likely to be discarded anyway. This naturally leads to the following idea. Rather than pruning the fully connected ClusterGraph iteratively, one can construct directly a k'-nearest neighbour graph on the n clusters, retaining for each cluster only the edges to its k' nearest neighbours. This yields a ClusterGraph that approximates the geometric structure of the dataset at a cost of $O(n^2)$, which is negligible when $n \ll |X|$.

The metric distortion (Equation 6) can then be evaluated once on this k'-NN ClusterGraph, providing a fast way to assess how well the graph captures the intrinsic geometry of the data for a given value of k'. Additionally, the standard pruning procedure described in this paper can be performed for such a k'-NN ClusterGraph at much lower cost.

2.5.2 Scalable landmark-based approximation

Most strategies presented above reduce constant factors or per-iteration costs but leave the asymptotic complexity unchanged, as the $O(|X|^2)$ bottleneck arising from the construction of the intrinsic distance matrix d_X^k persists regardless. We now propose a landmark-based approximation that addresses this bottleneck directly by reducing the effective dataset size from $|X|$ to $|X'| \ll |X|$. Rather than operating on all $|X|$ points, the pipeline is applied to a small set of representative points, or *landmarks*, selected to preserve both the global geometric structure of the dataset and the local intra-cluster structure. This yields a genuine asymptotic improvement across all steps whose complexity depends on $|X|$.

Given a dataset X of $|X|$ points partitioned into n clusters $\mathcal{C} = \{C_1, \ldots, C_n\}$, we construct a reduced dataset $X' \subset X$ as the union of two complementary sets of landmarks:

$$X' = L_m \cup \bigcup_{i=1}^{n} L_i, \qquad |X'| \approx m + \sum_{i=1}^{n} m_i \tag{13}$$

where

- L_m is a set of $m = \lfloor\sqrt{|X|}\rfloor$ global landmarks selected from X using the MaxMin algorithm [28], which iteratively selects the point furthest from all previously selected landmarks:

$$l_t = \arg\max_{x\in X} \min_{l\in L_{t-1}} d(x,l), \quad t = 1,\dots,m \tag{14}$$

  This guarantees uniform geometric coverage of the full dataset, capturing inter-cluster structure and boundary regions. The choice $m = \sqrt{|X|}$ is consistent with the Landmark Isomap literature [28], which establishes that $O(\sqrt{|X|})$ landmarks suffice for a good geodesic approximation.
- $L_i \subset C_i$ is a set of intra-cluster landmarks of size $m_i = \lfloor\sqrt{n_i}\rfloor$, where $n_i = |C_i|$ is the number of points in cluster C_i and $\lfloor\cdot\rfloor$ denotes the floor function. The landmarks are selected using MaxMin applied locally within each cluster C_i. This mirrors the global strategy at the cluster level, ensuring that each cluster is represented by a number of landmarks proportional to the square root of its size, guaranteeing uniform geometric coverage regardless of cluster size or shape.

Selecting landmarks randomly at both global and local levels also yields good results in practice [28]. The two sets serve complementary roles: L_m captures global inter-cluster geometry, while $\{L_i\}$ captures local intra-cluster structure. The total size of the reduced dataset is:

$$|X'| = m + \sum_{i=1}^{n} m_i = \sqrt{|X|} + \sum_{i=1}^{n} \sqrt{n_i} \tag{15}$$

By the Cauchy-Schwarz inequality, $\sum_{i=1}^{n} \sqrt{n_i} \leq \sqrt{n\sum_{i=1}^{n} n_i} = \sqrt{n|X|}$, and therefore:

$$|X'| = \sqrt{|X|} + \sum_{i=1}^{n} \sqrt{n_i} \leq \sqrt{|X|}(1+\sqrt{n}) = O(\sqrt{n|X|}) \tag{16}$$

All subsequent steps of the pipeline are performed on X' instead of X.

3 Results

3.1 Concentric circles

To showcase the whole ClusterGraph pipeline (Figure 2) we consider 500 points sampled from two concentric circles in the plane, depicted in Figure 3. Clusters are computed using k-means with 20 centroids and a ClusterGraph is built using the average Euclidean distance between points. The 10-nearest neighbours graph is used to estimate the intrinsic distance between data points. The iterative metric distortion pruning procedure is applied and the pruned ClusterGraph is showed in Figure 3(b). Note that the pruned ClusterGraph has two connected components, as a consequence of the 10-nearest neighbours graph having two connected components. Finally, the

two components are merged by adding an edge between each vertex and its 3-nearest neighbours in the other connected component. We then prune 20 of these newly introduced edges using the connectivity based approach. The resulting ClusterGraph is depicted in Figure 3(c).

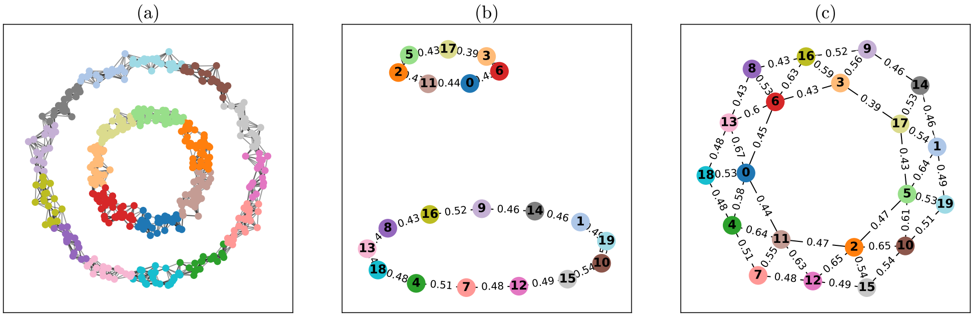

Fig. 3: ClusterGraph built on the output of k-means for 500 points sampled from two concentric circles of diameter 1 and 2. The 20 clusters are depicted in (a), on top of the 10-nn graph. The two metric distortion-pruned components are depicted in (b) and subsequently merged and connectivity pruned (c). Vertex colours are inherited from panel (a).

3.2 Mice protein expression

We analyse the expression levels of 77 proteins obtained from 38 normal genotype control mice and from 34 of their trisomic littermates, both with and without treatment with the drug memantine and with and without the stimulation to learn [29]. The original dataset (more details in Section 6) contains 15 measurements of each protein per sample, for a total of 1080 data points. Control mice learn successfully while the trisomic ones fail, unless they are first treated with memantine, which rescues their learning ability. The dataset is divided into four classes: mice that were not stimulated to learn (*no learning*, 555 samples), control mice that learned (*normal*, 285 samples), not treated and stimulated trisomic mice that failed to learn (*failed*, 105 samples) and treated and stimulated trisomic mice that learned successfully (*rescued*, 135 samples).

We reduce the dimensionality of the data by considering the first 31 principal components (95% of variance kept) and identify 18 clusters using k-means. The resulting ClusterGraph is depicted in Figure 4, alongside the output of popular dimensionality reduction techniques. On most layouts, one can observe two main regions. One is almost entirely composed of *no learning* samples. The second one is dominated by the *normal* group and is also containing the *rescued* and *failed* samples. State-of-the-art techniques such as UMAP and t-SNE are able to better separate the *no learning* samples from the others, but the two embeddings are drastically different. The ClusterGraph, on the other hand captures the same information and displays it in a cleaner, embedding-agnostic way.

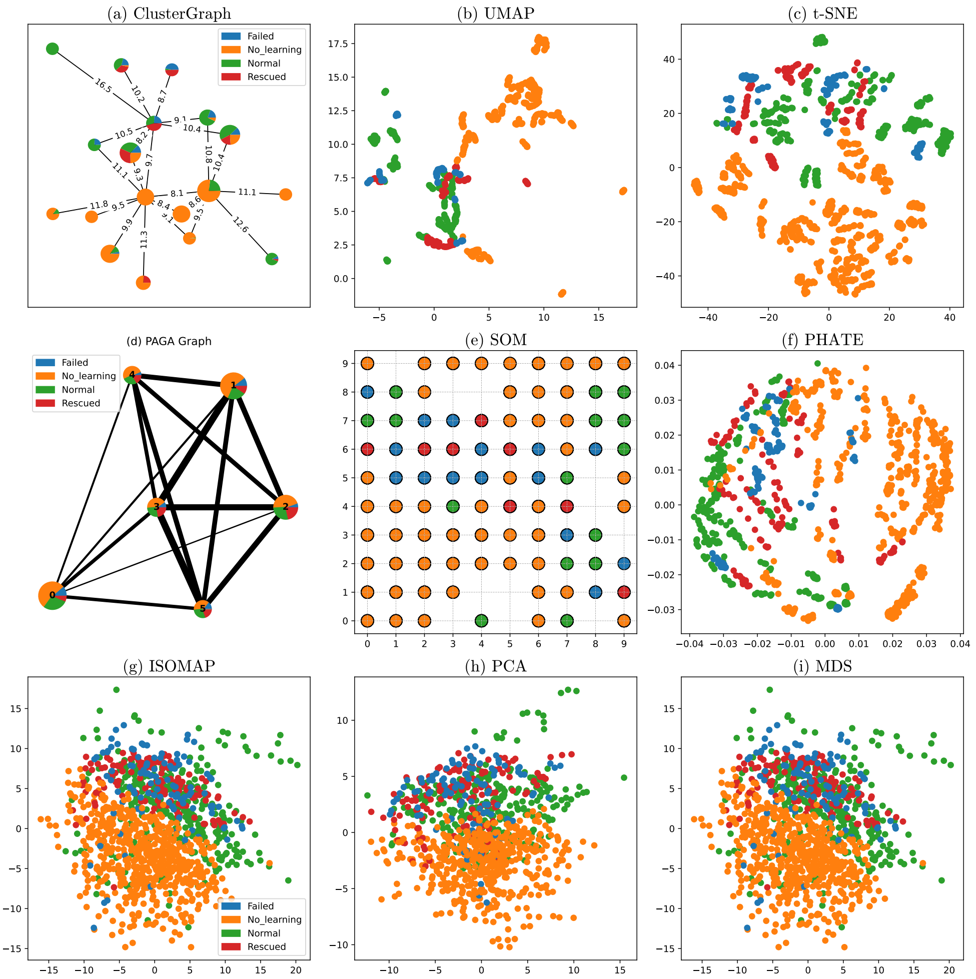

Fig. 4: ClusterGraph visualisation of the mice protein expression dataset alongside the output of other dimensionality reduction techniques. Each vertex in the ClusterGraph is depicted as a pie chart whose radius is proportional to the size of the corresponding cluster.

3.2.1 Assessing UMAP's layout with ClusterGraph

ClusterGraph can also be applied to the output of any dimensionality reduction algorithm, in order to assess the quality of the returned low-dimensional embedding. As already stated, many of these methods aim to preserve the local structure of the point cloud, but they offer no guarantees on the global layout, as demonstrated in the following example.

We focus our attention on the *failed* and *rescued* classes. Variable selection using a random forest method [5] was applied in order to identify the 10 most discriminating variables, this 10-dimensional point cloud is then visualised using UMAP (Figure 5(a)). This projection is able to separate well the two classes, moreover, some points appear to be outliers.

In order to quantify this observation, 10 clusters are selected from the two dimensional embedding using k-means (Figure 5(b)). A ClusterGraph is then built on top of them using the distance between clusters in the original 10-dimensional space. The connectivity pruned ClusterGraph is depicted in Figure 5(c), and it allows us to compare the organisation of the points in the original space versus the low dimensional UMAP embedding. In both visualisations cluster 9 appears to be the central one, which is consistent with it being composed of a mixture of points from the two classes. We can however spot some clear differences between the two layouts. Cluster 3, which is the outlier on the top left of the UMAP plot, is not an outlier in the ambient space, as it is closer to cluster 9 than, for example, cluster 6, which UMAP places close to the centre. Conversely, cluster 2, which appears to be near the centre in the UMAP plot, is at a significantly larger distance in the original space.

It is important to note that both visualisations agree with respect to the *local* layout, the differences appear at larger scales where UMAP fails to capture the global layout of the data.

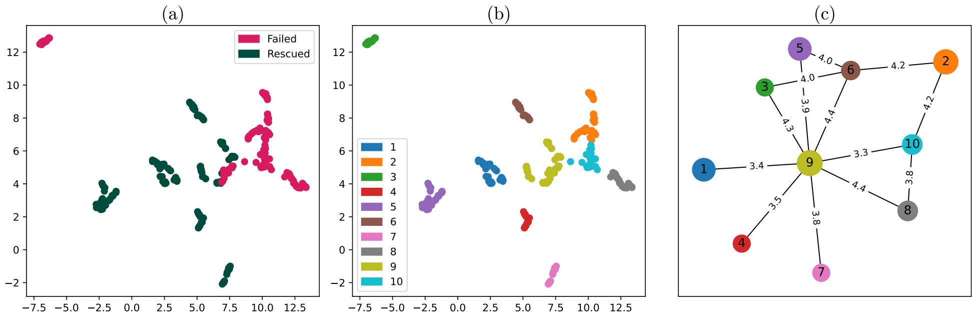

Fig. 5: UMAP visualisation of the *failed* and *rescued* samples is depicted in panels (a) and (b). Points are coloured by class in the former and by the output of a k-means clustering in the latter. Panel (c) depicts the ClusterGraph obtained from such clustering, where the distances are computed on the original space.

3.2.2 Multi-level ClusterGraph

In some scenarios we may be interested in further clustering a dataset which is already partitioned. This is the case in our working example as each sample belongs to one of four classes: no learning, normal, failed or rescued. A simple ClusterGraph obtained from such a coarse subdivision is depicted in Figure 6(a). Therefore, we could cluster samples of each class separately thus obtaining a finer partition that still respects the

class labels, i.e. all clusters are monochromatic with respect to the class label. We partition the samples of each class into sub-clusters: two for rescued and failed, four for normal, and five for no-learning by applying k-means to the data projected into the first 31 principal components corresponding to 95% of the variance kept using PCA. The number of sub-clusters for each class is chosen based on the class-specific inertia.

A connectivity-pruned version of this ClusterGraph, obtained by removing 56 edges, is shown in Figure 6(b). Figure 6(a) shows that the rescued and failed classes are closely related, a relationship further confirmed by Figure 6(b). Although the no learning and normal classes appear as the most distant clusters in Figure 6(a), this relationship is more nuanced and can be explained by the presence of an outlier (cluster 8), while clusters 9 and 7 remain connected to the no learning nodes.

Note that the global layout is consistent with the ClusterGraph built on the full dataset without class labels, as shown in Figure 6(a). This multi-level approach allows us to obtain a clearer visualisation by using the class labels as prior knowledge.

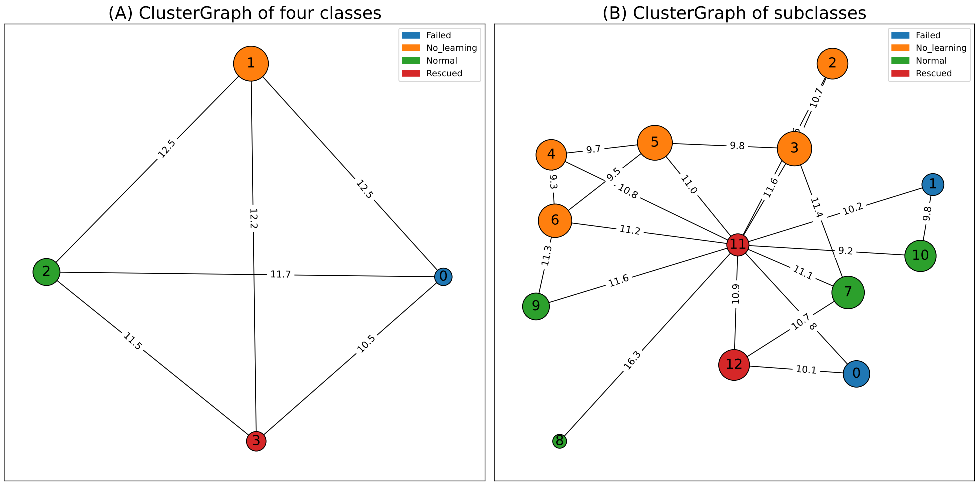

Fig. 6: A simple ClusterGraph where each cluster corresponds to one of the four classes is depicted in panel (a). Each cluster can be further subdivided using k-means (with k equal to two for rescued and failed, four for normal, and five for no-learning classes), the resulting connectivity pruned ClusterGraph is shown in panel (b). The radius of each vertex is proportional to the size of the corresponding cluster.

3.3 Bone marrow mononuclear cells

The data analysed in this section consists of bone marrow mononuclear cells of healthy human donors [30] and was part of the NeurIPS 2021 OpenProblem benchmarking dataset. The dataset consists of the expression levels of 23427 genes for 17041 samples. After normalisation, we identify the top 2000 highly variable genes and computed the first 50 principal components. We then compute the neighbourhood graph of cells

using the PCA representation of the data matrix and clustered it using the Leiden algorithm [31], separating it into 14 clusters.

Figure 7 depicts in panel (a) the two-dimensional UMAP projection of the first 50 principal components, coloured by the corresponding cluster. We then computed the ClusterGraph of the first 50 principal components, using as input the 14 clusters found by the Leiden algorithm. A connectivity-pruned ClusterGraph is shown in Figure 7 panel (b), whose edge labels indicate the average distance between two clusters.

We can observe how both visualisation techniques agree on the local organisation of clusters, e.g. clusters $3, 8, 11$ are close together, as well as clusters $5, 9, 13$. At the same time, ClusterGraph allows us to gain insights into the global layout that are hidden by the two-dimensional constraints of the UMAP plot. For example, cluster 6 is actually closer to cluster 0 than cluster 2.

3.4 Human lung cancer cell lines

The last dataset [32] consists of single cell RNA-seq of five human lung adenocarcinoma cell lines HCC827, H838, H2228, H1975 and A549. The five lung cancer cell lines were profiled using the 10X Chromium single-cell RNA sequencing platform.

We analyse the raw count matrix with genes represented as columns. A preprocessing pipeline is applied to retain only the most informative genes, focusing on those with high variability. Principal Component Analysis (PCA) is then performed, and the top 40 principal components are retained for downstream analysis. The resulting dataset is visualised using both UMAP and ClusterGraph, as shown in Figure 8.

In Figure 8, the UMAP embedding reveals five clearly separated clusters, which largely correspond to the five cell lines. The green (H1975) and pink clusters (H838) appear closer together, while the blue (A549) cluster is positioned farther away. This organisation suggests that the green (H1975) and pink (H838) cell populations are more similar to each other than either is to the blue one (A549). However, this interpretation can be further confirmed using ClusterGraph. By comparing cluster 8 (green cells) with cluster 5 (blue cells) and cluster 6 (pink cells), we observe that the distances from cluster 8 to both cluster 5 and cluster 6 are approximately equal. This indicates that the blue (A549) cluster is not an outlier, but is as distinct from the green (H1975) cluster as the pink (H838) cluster is. ClusterGraph thus provides a more balanced and interpretable view of the relationships between clusters that may not be fully captured in UMAP alone.

Another insight provided by the ClusterGraph is the ability to decompose each group into subgroups using algorithms such as KMeans as illustrated in Figure 8 (a). For instance, in the UMAP embedding, the pink (H838) cluster appears more dispersed compared to the compact purple (H2228) cluster, which might suggest potential subpopulations or varying cellular states within the pink (H838) group. However, when examining the ClusterGraph, the distances between the purple (H2228) sub-clusters are actually greater than those within the pink cluster. This highlights how ClusterGraph can offer a more nuanced perspective on intra-cluster relationships that may not be immediately apparent in UMAP plots.

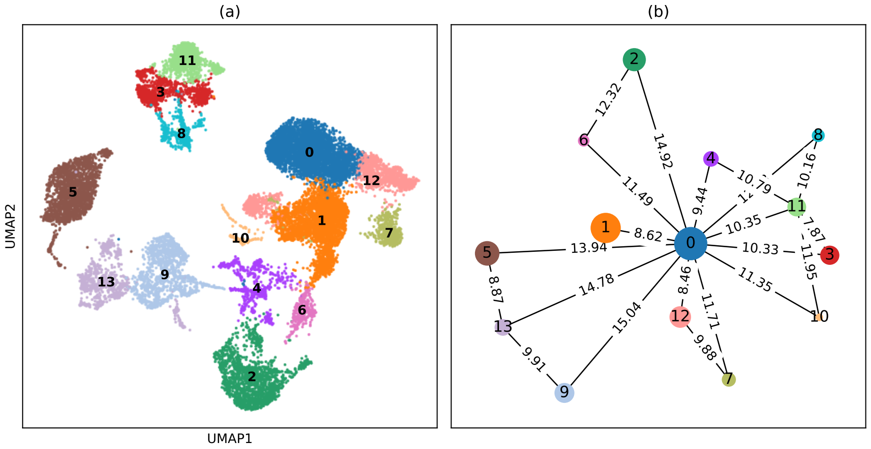

Leiden 0.5

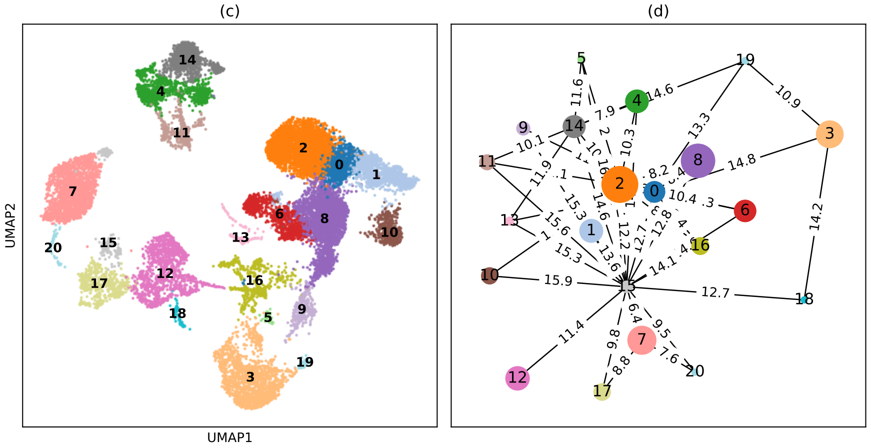

Leiden 0.75

Fig. 7: UMAP plots (a, c) and ClusterGraph (b, d) of the bone marrow single cell dataset for two different Leiden thresholds. The colours correspond to the clusters found by the Leiden algorithm.

3.5 ClusterGraph stability

The construction of ClusterGraph involves two key ingredients: a clustering scheme and a method for approximating geodesic distances. Because a systematic study of all possible choices is impractical, we focus on a single standard setting, using K-means for clustering and a k-NN graph for geodesic distance estimation, and examine how the behaviour of ClusterGraph changes as the corresponding parameters are varied over a suitable range. To avoid ambiguity, we write k_{KNN} for the number of neighbours

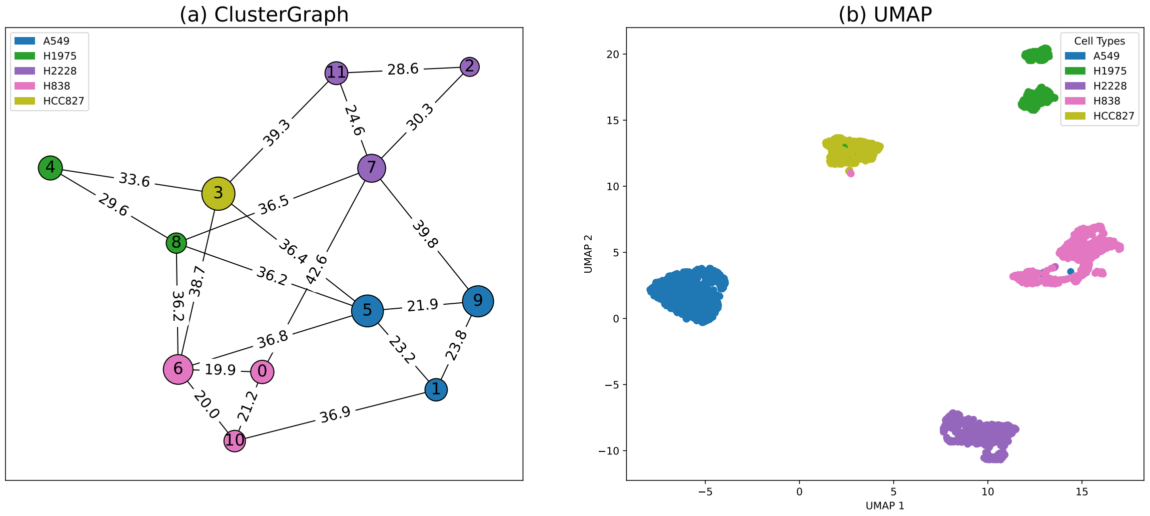

Fig. 8: Visualisation of the five cell line dataset using ClusterGraph and UMAP. (a) ClusterGraph representation generated using KMeans clustering with k = 12. Each node represents a cluster and is coloured according to its dominant cell population, defined as the label shared by at least 98% of the cells within the cluster. (b) UMAP projection. The colour scheme corresponds to the five cell lines: HCC827, H838, H2228, H1975, and A549.

in the k-NN graph used to approximate geodesic distances, and K for the number of clusters in K-means.

3.5.1 Sensitivity to k_{KNN}

The parameter k_{KNN} controls the connectivity of the k-NN graph used for geodesic distance estimation: too small a value risks graph disconnection and infinite geodesic distances, while too large a value introduces shortcuts between geometrically distant points, causing estimated distances to converge towards Euclidean distances and eroding manifold structure. The choice of k_{KNN} is therefore important, as the primary goal is to approximate geodesic distances faithfully while preserving the manifold structure of the data.

To assess the sensitivity of the ClusterGraph to k_{KNN}, we run a grid search over k_{KNN} values ranging from 5 to 30 depending on the dataset, for several fixed values of K. For each combination, a k-NN graph is constructed on the full point cloud, geodesic distances are estimated, and the metric distortion $\Delta_k(G)$ is recorded. This procedure is repeated across four datasets: **Mice Protein**, **Lung Cancer**, **Diabetes**, and **Concentric Circles**, covering both real biological data and synthetic geometric benchmarks.

Figure 9 shows the metric distortion as a function of k_{KNN}, for several fixed values of K. A consistent pattern emerges across all datasets: metric distortion is higher and less stable for small values of k_{KNN}, then decreases and stabilises as k_{KNN} increases. This reflects the transition from an under-connected graph where geodesic estimates are noisy or unreliable, to a well-connected graph where distances stabilise. Beyond this stable region, increasing k_{KNN} further risks introducing shortcuts, degrading the geodesic approximation.

In practice, the recommended strategy is to identify the *elbow* of the distortion curve: the smallest k_{KNN} at which the metric distortion enters its stable plateau, balancing approximation quality against the risk of shortcut-induced bias. Since recomputing metric distortion over a dense grid of k_{KNN} values can be computationally expensive, the principled alternatives described below, which follow the same underlying logic, can guide this selection with lower overhead.

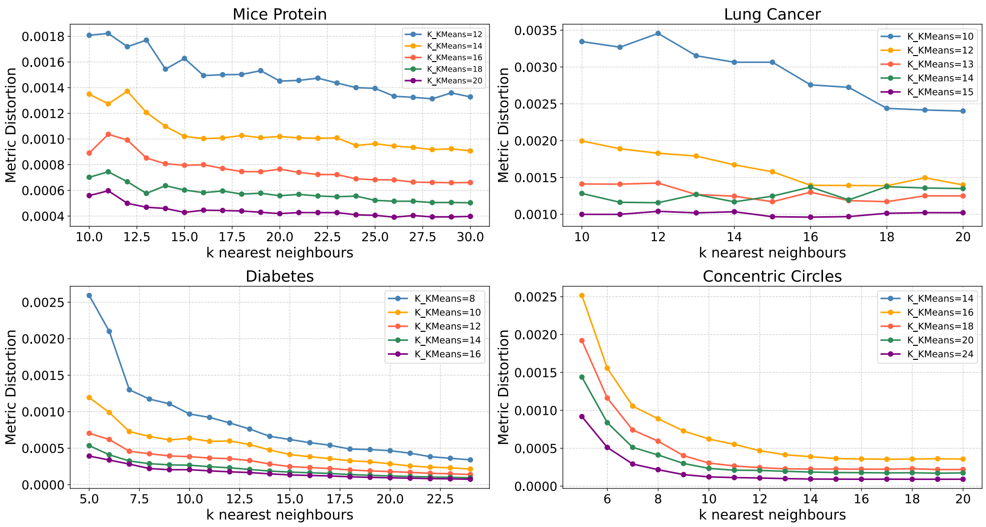

Fig. 9: Sensitivity analysis on the **Mice Protein**, **Lung Cancer**, **Concentric Circles** and **Diabetes** datasets as a function of the number of nearest neighbours, each line corresponding to a fixed value of k_{KNN}.

A natural first diagnostic for selecting k_{KNN} is to monitor the number of connected components of the k-NN graph as k_{KNN} increases. When several components persist despite increasing k_{KNN}, this may indicate that the dataset is a union of multiple distinct manifolds, a structure that should be preserved during metric distortion pruning so that the resulting ClusterGraph faithfully reflects the geometry of the data. The relationships between these manifolds can then be explored through the merging process (Section 2.4).

The problem of selecting k_{KNN} automatically has been studied in the context of Isomap, leading to several principled approaches. Samko et al. [33] propose minimising the residual variance of the Isomap embedding across multiple values of k_{KNN},

while Jing and Shao [34] suggest monitoring the sum of shortest paths as k_{KNN} increases. Both methods look for an elbow in the resulting curve, identifying the smallest k_{KNN} beyond which the geodesic structure stabilises, the same logic underlying the metric distortion plateau identified here. Once k_{KNN} is chosen, the quality of the geodesic approximation can be further improved by removing shortcut edges using betweenness-based filtering [35], which identifies edges that are disproportionately frequent in shortest paths and are therefore likely to represent spurious cross-manifold connections. Removing such shortcuts yields a cleaner k-NN graph, a more faithful geodesic approximation, and ultimately a more meaningful metric distortion.

In practice, we recommend $k_{\mathrm{KNN}} = 15$ as a default starting point, consistent with the literature on geodesic distance estimation, and we advise verifying the connectivity of the resulting k-NN graph as a sanity check.

3.5.2 Sensitivity to clustering granularity

To assess the sensitivity of the ClusterGraph to the parameter K of K-Means, we run a grid search over K values ranging from 5 to 24 depending on the dataset, for several fixed values of k_{KNN}. For each combination, a ClusterGraph is constructed and the metric distortion $\Delta_k(G)$ is recorded. The same four datasets are used: **Mice Protein** ($K \in [10, 20]$, $k_{\mathrm{KNN}} \in [15, 25]$), **Lung Cancer** ($K \in [10, 15]$, $k_{\mathrm{KNN}} \in [10, 20]$), **Diabetes** ($K \in [5, 15]$, $k_{\mathrm{KNN}} \in [8, 18]$), and **Concentric Circles** ($K \in [16, 24]$, $k_{\mathrm{KNN}} \in [8, 18]$) to evaluate consistency across both real biological data and synthetic geometric benchmarks.

Fiure 10 indicates that the dependence of ClusterGraph on clustering granularity is stable, in the sense that changing K does not produce abrupt changes in the score, but rather a gradual resolution-dependent effect.

3.5.3 Robustness to clustering noise

In practice, clustering assignments are never perfect: automated algorithms may mislabel at cluster boundaries, and biological noise can blur the separation between populations. To evaluate how sensitive ClusterGraph is to such imperfections, we simulate label noise by randomly reassigning a fraction p of points to a different cluster chosen uniformly at random among all clusters other than their own. This corruption procedure is applied at rates $p \in \{2\%, 5\%, 7\%, 10\%, 12\%, 15\%\}$, and for each rate, 10 independent random seeds are used to average out stochastic variation, yielding a mean metric distortion $\overline{\Delta}_k$ per condition. The k-NN graph is fixed at dataset-specific values ($k_{\mathrm{KNN}} = 15$ for **Mice Protein**, $k_{\mathrm{KNN}} = 14$ for **Lung Cancer**, and $k_{\mathrm{KNN}} = 10$ for **Diabetes** and **Concentric Circles**), and three values of K are tested per dataset. A dashed horizontal line in each panel of Figure 11 indicates the reference metric distortion obtained without any corruption, serving as a baseline for comparison.

As shown in Figure 11, the ClusterGraph exhibits strong robustness to label noise for most datasets and parameter settings: the average metric distortion remains close to the uncorrupted baseline even at corruption rates as high as 15%, with curves staying nearly flat across the full range of p. The effect is more pronounced for coarser clusterings (smaller K), where each cluster aggregates more cells and a few mislabelled points have a larger relative impact on inter-cluster distance estimates. Finer

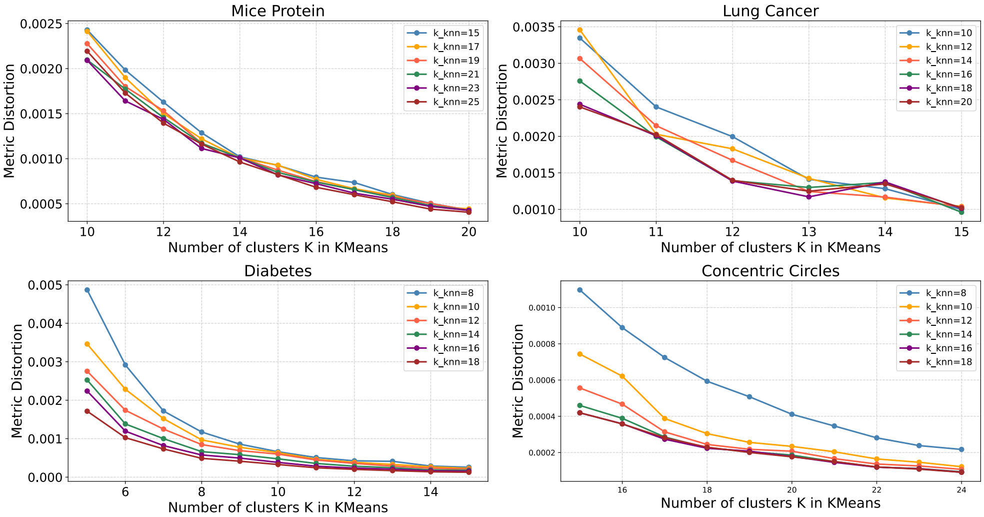

Fig. 10: Sensitivity of the metric distortion to K, with k_{KNN} fixed, across four datasets: **Mice Protein** (top left), **Lung Cancer** (top right), **Diabetes** (bottom left), and **Concentric Circles** (bottom right). Each line corresponds to a fixed value of k_{KNN}. Metric distortion decreases monotonically with K across all conditions, confirming that finer clusterings consistently reduce geometric distortion.

clusterings are more stable, as individual corrupted points represent a smaller fraction of each cluster. Notably, the **Concentric Circles** dataset exhibits the highest sensitivity to label noise. This can be attributed to its structure: the dataset consists of two geometrically disconnected components, yet the corruption procedure was applied globally at the dataset level rather than independently within each component. As a result, corrupted points may be reassigned across components, introducing inter-component label swaps that would never arise from a realistic clustering error, and artificially inflating the measured distortion.

Furthermore, the corruption model used here represents a worst-case scenario: in practice, clustering errors are predominantly local, i.e. a cell is far more likely to be mislabelled into a neighbouring cluster than into a distant, unrelated one. The global random reassignment applied here therefore overestimates the true impact of label noise, suggesting that the ClusterGraph is even more robust in practice than Figure 11 indicates.

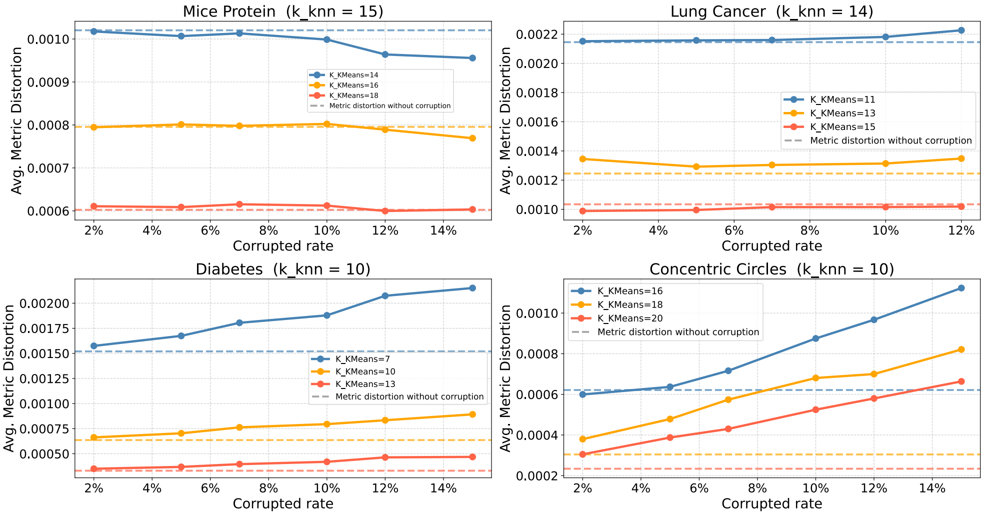

Fig. 11: Robustness of the metric distortion to clustering label noise, across four datasets: **Mice Protein** ($k_{\mathrm{KNN}} = 15$, top left), **Lung Cancer** ($k_{\mathrm{KNN}} = 14$, top right), **Diabetes** ($k_{\mathrm{KNN}} = 10$, bottom left), and **Concentric Circles** ($k_{\mathrm{KNN}} = 10$, bottom right). Each solid line shows the average metric distortion over 10 independent corruptions at a given rate, for a fixed K. Dashed lines indicate the reference distortion with no corruption.

4 Discussion

ClusterGraph provides a simple, noise-robust, and effective framework for compressing and visualising high-dimensional data. By representing clusters as vertices and inter-cluster relationships as weighted edges, it captures the large-scale organisation of a dataset without forcing the data into a two-dimensional Euclidean embedding. After pruning, the resulting graph yields a compact summary of the intrinsic metric structure of the data.

A central advantage of the method is that it comes with a built-in quality criterion. Metric distortion quantifies how faithfully the graph reflects the intrinsic geometry of the dataset, thereby providing a principled basis for pruning, model selection, and parameter tuning.

ClusterGraph should be viewed as complementary to methods such as UMAP, t-SNE, and PHATE. Although these methods are often effective at preserving local neighbourhood structure, they can distort global relationships between data points. ClusterGraph addresses this limitation by encoding inter-cluster geometry directly in graph form. It therefore serves not only as a visualisation tool, but also as a diagnostic layer that can validate, refine, or question conclusions drawn from low-dimensional embeddings.

5 Availability of Source Code and Requirements

Project name: ClusterGraph
Project homepage: https://github.com/dioscuri-tda/ClusterGraph
Operating system: Linux, macOS, Windows
Programming language: Python
Other requirements: NumPy, pandas, NetworkX, Matplotlib, Bokeh, scikit-learn, POT (Python Optimal Transport)
License: MIT license
RRID: SCR 027405

6 Data availability

All real-world datasets used in this article are publicly available and can be found at:

- Mice protein expression data [29]
- Bone marrow mononuclear cells of healthy human donors [30]
- Single-Cell RNA-seq of five human lung cancer cell lines [32] with the GEO accession number GSM3618014.
- Diabetes Reaven and Miller (1979) [36]

Acknowledgments. PD, DG and MH acknowledge support by the Dioscuri program initiated by the Max Planck Society, jointly managed with the National Science Centre (Poland), and mutually funded by the Polish Ministry of Science and Higher Education and the German Federal Ministry of Education and Research. The publication was created as part of the project "Center for Trustworthy Artificial Intelligence for Life Sciences" implemented under the International Research Agendas programme of the Foundation for Polish Science co-financed by the European Union under the European Funds for Smart Economy 2021-2027 (FENG).

Authors' contributions. PD conceived and directed the project. All the authors collaborated on the mathematical aspects, the design of the algorithm and the writing of the manuscript. DG directed the writing of the manuscript. MH, under the supervision of DG, developed the software, collected and analysed the experimental data and defined the evaluation metric. AJL helped in writing and revision of the manuscript.

References

[1] Pearson, K.: On lines and planes of closest fit to systems of points in space. The London, Edinburgh, and Dublin Philosophical Magazine and Journal of Science **2**(11), 559–572 (1901) https://doi.org/10.1080/14786440109462720

[2] Maaten, L., Hinton, G.: Visualizing data using t-SNE. Journal of Machine Learning Research **9**, 2579–2605 (2008)

[3] McInnes, L., Healy, J., Melville, J.: UMAP: Uniform Manifold Approximation and Projection for Dimension Reduction. ArXiv e-prints (2018) arXiv:1802.03426 [stat.ML]

[4] Moon, K.R., Van Dijk, D., Wang, Z., Gigante, S., Burkhardt, D.B., Chen, W.S., Yim, K., Van Den Elzen, A., Hirn, M.J., Coifman, R.R., Ivanova, N.B., Wolf, G., Krishnaswamy, S.: Visualizing Structure and Transitions for Biological Data Exploration (2017) https://doi.org/10.1101/120378 . Institution: Bioinformatics Type: preprint. Accessed 2024-01-27

[5] Hastie, T., Friedman, J., Tibshirani, R.: The Elements of Statistical Learning. Springer Series in Statistics. Springer, New York, NY (2001). https://doi.org/10.1007/978-0-387-21606-5 . http://link.springer.com/10.1007/978-0-387-21606-5 Accessed 2023-07-11

[6] Saxena, A., Prasad, M., Gupta, A., Bharill, N., Patel, O.P., Tiwari, A., Er, M.J., Ding, W., Lin, C.-T.: A review of clustering techniques and developments. Neurocomputing **267**, 664–681 (2017) https://doi.org/10.1016/j.neucom.2017.06.053

[7] Klamann, C., Lau, C.J., Ruiz-Ramírez, J., Schwartz, G.W.: TooManyCellsInteractive: A visualization tool for dynamic exploration of single-cell data. GigaScience **13**, 056 (2024) https://doi.org/10.1093/gigascience/giae056 . Accessed 2026-04-08

[8] Zhou, Y., Tang, C., Xiao, X., Zhan, X., Wang, T., Xiao, G., Xu, L.: Dimensionality reduction for visualizing spatially resolved profiling data using SpaSNE. GigaScience **14**, 002 (2025) https://doi.org/10.1093/gigascience/giaf002 . Accessed 2026-04-08

[9] Kariotis, S., Tan, P.F., Lu, H., Rhodes, C.J., Wilkins, M.R., Lawrie, A., Wang, D.: Omada: robust clustering of transcriptomes through multiple testing. GigaScience **13**, 039 (2024) https://doi.org/10.1093/gigascience/giae039 . Accessed 2026-04-08

[10] Zhang, D., Yu, N., Yuan, Z., Li, W., Sun, X., Zou, Q., Li, X., Liu, Z., Zhang, W., Gao, R.: stmmr: accurate and robust spatial domain identification from spatially resolved transcriptomics with multimodal feature representation. GigaScience **13**, 089 (2024) https://doi.org/10.1093/gigascience/giae089 https://academic.oup.com/gigascience/article-pdf/doi/10.1093/gigascience/giae089/60885040/giae089.pdf

[11] Song, W.-M., Ming, C., Forst, C.V., Zhang, B.: Unsupervised multiscale clustering of single-cell transcriptomes to identify hierarchical structures of cell subtypes. GigaScience **14**, 111 (2025) https://doi.org/10.1093/gigascience/giaf111 https://academic.oup.com/gigascience/article-pdf/doi/10.1093/gigascience/giaf111/64573732/giaf111.pdf

[12] Morgan, C.L.: Embedding metric spaces in Euclidean space. Journal of Geometry **5**(1), 101–107 (1974) https://doi.org/10.1007/BF01954540 . Accessed 2024-03-06

[13] Bourgain, J.: On lipschitz embedding of finite metric spaces in Hilbert space. Israel Journal of Mathematics **52**(1), 46–52 (1985) https://doi.org/10.1007/BF02776078 . Accessed 2024-03-06

[14] Höppner, F., Klawonn, F., Kruse, R., Runkler, T.: Fuzzy Cluster Analysis. Wiley IBM PC Series. John Wiley & Sons, Chichester, England (1999)

[15] Singh, G., Memoli, F., Carlsson, G.: Topological Methods for the Analysis of High Dimensional Data Sets and 3D Object Recognition. The Eurographics Association, (2007). https://doi.org/10.2312/SPBG/SPBG07/091-100 . Accepted: 2014-01-29T16:52:11Z ISSN: 1811-7813. https://diglib.eg.org:443/xmlui/handle/10.2312/SPBG.SPBG07.091-100 Accessed 2023-04-27

[16] Dłotko, P.: Ball mapper: a shape summary for topological data analysis. arXiv. arXiv:1901.07410 [math] (2019). https://doi.org/10.48550/arXiv.1901.07410 . http://arxiv.org/abs/1901.07410 Accessed 2023-04-27

[17] Rubner, Y., Tomasi, C., Guibas, L.J.: The Earth Mover's Distance as a Metric for Image Retrieval. International Journal of Computer Vision **40**(2), 99–121 (2000) https://doi.org/10.1023/A:1026543900054 . Accessed 2023-04-28

[18] Wasserstein, L.N.: Markov processes over denumerable products of spaces describing large systems of automata. Problems of Information Transmission **5**(1), 47–52 (1969)

[19] Dłotko, P., Gurnari, D., Sazdanovic, R.: Mapper–type algorithms for complex data and relations. Journal of Computational and Graphical Statistics, 1–14 (2024) https://doi.org/10.1080/10618600.2024.2343321 https://doi.org/10.1080/10618600.2024.2343321

[20] Tenenbaum, J.B., Silva, V.d., Langford, J.C.: A Global Geometric Framework for Nonlinear Dimensionality Reduction. Science **290**(5500), 2319–2323 (2000) https://doi.org/10.1126/science.290.5500.2319 . Publisher: American Association for the Advancement of Science. Accessed 2024-03-27

[21] Klein, J., Zachmann, G.: Point cloud surfaces using geometric proximity graphs. Computers & Graphics **28**(6), 839–850 (2004) https://doi.org/10.1016/j.cag.2004.08.012 . Accessed 2024-03-27

[22] Ruggeri, M.R., Darom, T., Saupe, D., Kiryati, N.: Approximating geodesics on point set surfaces. In: Proceedings of the 3rd Eurographics / IEEE VGTC Conference on Point-Based Graphics. SPBG'06, pp. 85–94. Eurographics Association, Goslar, DEU (2006)

[23] Yu, H., Zhang, J.J., Jiao, Z.: Geodesics on Point Clouds. Mathematical Problems in Engineering **2014**, 860136 (2014) https://doi.org/10.1155/2014/860136 . Publisher: Hindawi. Accessed 2023-04-27

[24] Bernstein, M., Silva, V., Langford, J.C., Tenenbaum, J.B.: Graph approximations to geodesics on embedded manifolds. (2000)

[25] Narasimhan, G., Smid, M.: Geometric Spanner Networks. Cambridge University Press, Cambridge (2007). https://doi.org/10.1017/CBO9780511546884

[26] Zhou, F., Mahler, S., Toivonen, H.: Simplification of Networks by Edge Pruning. In: Berthold, M.R. (ed.) Bisociative Knowledge Discovery: An Introduction to Concept, Algorithms, Tools, And Applications, pp. 179–198. Springer, Berlin, Heidelberg (2012). https://doi.org/10.1007/978-3-642-31830-6_13

[27] Malkov, Y.A., Yashunin, D.A.: Efficient and robust approximate nearest neighbor search using hierarchical navigable small world graphs. IEEE Transactions on Pattern Analysis and Machine Intelligence **42**(4), 824–836 (2020) https://doi.org/10.1109/TPAMI.2018.2889473

[28] Silva, V., Tenenbaum, J.: Sparse multidimensional scaling using landmark points. Technology (2004)

[29] Higuera, C., Gardiner, K.J., Cios, K.J.: Self-Organizing Feature Maps Identify Proteins Critical to Learning in a Mouse Model of Down Syndrome. PLOS ONE **10**(6), 0129126 (2015) https://doi.org/10.1371/journal.pone.0129126 . Publisher: Public Library of Science. Accessed 2024-04-11

[30] Luecken, M.D., Büttner, M., Chaichoompu, K., Danese, A., Interlandi, M., Mueller, M.F., Strobl, D.C., Zappia, L., Dugas, M., Colomé-Tatché, M., Theis, F.J.: Benchmarking atlas-level data integration in single-cell genomics. Nature Methods **19**(1), 41–50 (2022) https://doi.org/10.1038/s41592-021-01336-8

[31] Traag, V.A., Waltman, L., Eck, N.J.: From louvain to leiden: guaranteeing well-connected communities. Scientific Reports **9**(1), 5233 (2019) https://doi.org/10.1038/s41598-019-41695-z

[32] Tian, L., Dong, X., Freytag, S., Lê Cao, K.-A., Su, S., JalalAbadi, A., Amann-Zalcenstein, D., Weber, T.S., Seidi, A., Jabbari, J.S., Naik, S.H., Ritchie, M.E.: Benchmarking single cell RNA-sequencing analysis pipelines using mixture control experiments. Nature Methods **16**(6), 479–487 (2019) https://doi.org/10.1038/s41592-019-0425-8

[33] Samko, O., Marshall, A.D., Rosin, P.L.: Selection of the optimal parameter value for the isomap algorithm. Pattern Recognition Letters **27**(9), 968–979 (2006) https://doi.org/10.1016/j.patrec.2005.11.017

[34] Jing, L., Shao, C.: Selection of the suitable parameter value for isomap. J. Softw. **6**, 1034–1041 (2011)

[35] Cukierski, W.J., Foran, D.J.: Using betweenness centrality to identify manifold shortcuts. In: 2008 IEEE International Conference on Data Mining Workshops, pp. 949–958 (2008). https://doi.org/10.1109/ICDMW.2008.39

[36] Reaven, G.M., Miller, R.G.: An attempt to define the nature of chemical diabetes using a multidimensional analysis. Diabetologia **16**(1), 17–24 (1979) https://doi.org/10.1007/BF00423145 . Accessed 2023-04-28

[37] Fredman, M.L., Tarjan, R.E.: Fibonacci heaps and their uses in improved network optimization algorithms. J. ACM **34**(3), 596–615 (1987) https://doi.org/10.1145/28869.28874

A Complexity Analysis

We analyse the complexity of ClusterGraph's pipeline for $|X|$ points in $\mathbb{R}^d$, n clusters, and k, the number of neighbours in the k-NN graph.

A.1 ClusterGraph Construction

The construction complexity depends on the chosen inter-cluster metric. With centroid-based distances, computing each centroid requires a single pass over the cluster points at cost $O(|X|d)$, and evaluating all pairwise centroid distances costs $O(n^2 d)$, giving a total of $O(|X|d + n^2 d)$. When using average, minimum, or maximum inter-cluster distances, all pairwise point distances across every cluster pair must be evaluated, costing $O(|X|^2)$ regardless of cluster size distribution.

A.2 Metric Distortion

Computing the metric distortion requires building a k-NN graph over $|X|$ points and running all-pairs shortest paths (APSP) to populate the full $|X| \times |X|$ intrinsic distance matrix d_X^k. The k-NN graph construction costs $O(|X|^2 d)$ with exact methods, or $O(|X| \log |X|)$ with approximate methods such as HNSW [27]. Once the graph is built, Dijkstra's algorithm with a Fibonacci heap [37] computes APSP at a cost of $O(|X|^2 k + |X|^2 \log |X|)$. Combining both steps gives:

$$\text{Exact:} \quad O\big(|X|^2(d + k + \log |X|)\big) \tag{17}$$

$$\text{Approximate:} \quad O\big(|X|^2(k + \log |X|)\big) \tag{18}$$

Since APSP dominates k-NN graph construction in the approximate case, the overall complexity of the Metric Distortion for a given ClusterGraph reduces to $O\big(|X|^2 \log |X|\big)$, treating k and d as constants.

The Metric Distortion pruning step's complexity depends on both the chosen pruning algorithm and the initial graph structure. As described in Section 2.5.1, starting from a k'-NN–based ClusterGraph rather than the fully connected graph can significantly reduce the number of edges to prune.

A.3 Landmark-based approximation

To reduce complexity, we approximate ClusterGraph using a landmark set selected by the MaxMin algorithm. For a set of m landmarks over $|X|$ points, MaxMin costs $O(|X|m)$. Applying it globally and within each cluster (see Section 2.5.2) yields a total landmark selection cost of:

$$O\left(|X|\sqrt{|X|} + \sum_{i=1}^{n} n_i\sqrt{n_i}\right) = O\big(|X|^{3/2}\big). \tag{19}$$

The resulting reduced dataset X' contains $O(\sqrt{n|X|})$ points. The subsequent pipeline on X' costs $O(\sqrt{n|X|}\, d + n^2 d)$ for centroid distance computation and $O\big(n|X|(k + \log\sqrt{n|X|})\big)$ for the approximate k-NN graph and APSP. Since $n \ll |X|$, both terms are absorbed into $O\big(|X|^{3/2}\big)$, giving an overall landmark pipeline complexity of $O\big(|X|^{3/2}\big)$.

A.4 Comparison with related methods

Table 1 compares ClusterGraph's complexity against standard dimensionality reduction methods. The landmark approximation achieves $O(|X|^{3/2})$, sitting between exact ClusterGraph and UMAP.

Method	Complexity
Isomap [20]	$O(\lvert X\rvert^3)$
ClusterGraph exact	$O(\lvert X\rvert^2 \log \lvert X\rvert)$
t-SNE [2]	$O(\lvert X\rvert^2)$
ClusterGraph landmark	$O(\lvert X\rvert^{3/2})$
UMAP [3]	$O(\lvert X\rvert^{1.14})$

Table 1: Complexity comparison of ClusterGraph and standard dimensionality reduction methods. d is treated as a constant throughout.

Response letter

Click here to access/download

Supplementary Material

ClusterGraph_reviews_answer_letter.docx

Personal Cover

Click here to access/download;Personal Cover;Cover_letter_ClusterGraph.pdf

Mathis Hallier

Dioscuri Centre in Topological Data Analysis,
Mathematical Institute, Polish Academy of Sciences,
Sniadeckich 8, Warsaw, Poland

mathis.hallier28@gmail.com

Dear Dr Nogoy and the Editorial Team,

We are writing to resubmit our manuscript titled "**ClusterGraph: a new tool for visualization and compression of multidimensional data**" for your consideration in *GigaScience*. This revised version follows your earlier editorial decision, in which you requested the inclusion of missing sections, additional benchmarking, and other specific enhancements for further consideration.

We are pleased to report that we have thoroughly addressed all points raised in your letter. In particular:

Source Code & Requirements
We have added a dedicated *Source Code & Requirements* section to the manuscript. The full implementation of *ClusterGraph* is now openly available as a public Python library, with clear installation instructions, environment details, example usage, and all scripts necessary to reproduce the figures and experiments in the paper.

Data Availability
We now clearly list all datasets used in the study, emphasizing accessibility and reproducibility. All datasets, including those for benchmarking, are openly available either through public repositories or via the project's GitHub page. Where applicable, we have provided accession numbers, and the Data Availability section contains all relevant links and metadata.

Benchmarking and Comparison to Existing Tools

We have substantially expanded the manuscript to include a comprehensive benchmarking section. In this section, we compare *ClusterGraph* with eight established visualization tools, including UMAP, t-SNE, and PCA. We evaluate performance across multiple datasets, highlighting key differences and demonstrating how *ClusterGraph* can detect and correct misleading visualizations—particularly with respect to preserving the global data structure.

Single-Cell Data Experiments

In response to your suggestion, we have added two new experiments analyzing publicly available single-cell datasets. These demonstrate the utility of *ClusterGraph* in the context of single-cell data analysis and confirm its ability to provide robust insights into subgroup structure in highly complex, high-dimensional data.

We believe that the manuscript has been improved considerably, both in scientific content and reproducibility, and that it addresses your requests regarding openness, benchmarking, and broader relevance. We kindly submit this revised version for your consideration in *GigaScience*.

We greatly appreciate your time and the constructive feedback, which has helped us enhance our work. We look forward to hearing from you.

Sincerely,

Mathis Hallier
