# Supplementary material for: ClusterGraph: a new tool for visualisation and compression of multidimensional data
Source: Gigascience. 2026 Jun 13;15:giag070. doi: 10.1093/gigascience/giag070 (PMC13354933; doi:10.1093/gigascience/giag070)
Supplement: giag070_Authors_Response_To_Reviewer_Comments_original_submission [file giag070_authors_response_to_reviewer_comments_original_submission.pdf]

Author's Response To Reviewer Comments

Dear Editor,
We would like to express our gratitude to the reviewers for their insightful comments and suggestions, which have significantly contributed to enhancing our manuscript. Below, we provide detailed explanations of how each comment has been addressed. All the changes are marked in green in the manuscript pdf. Reviewer comments are marked between "***Reviewer" for
clarity, with our responses immediately following each point.

Thank you for your assistance in this process.
Best wishes,
Mathis Hallier (on behalf of all authors)

************* Reviewer
Reviewer reports:

Reviewer #1: This manuscript introduces ClusterGraph, a graph-based abstraction built on top of clustering results, aiming to capture and visualize the global organization of high-dimensional data while avoiding distortions inherent to low-dimensional embeddings.

1.In realistic high-dimensional biological or social datasets, many assumptions are rarely verifiable. The manuscript would benefit from a discussion of failure modes, robustness to noisy clustering, and sensitivity to the choice of k in k-NN graphs.
************* End Reviewer

We thank the Reviewer for this important comment. We agree that, in realistic high-dimensional biological or social datasets, the assumptions underlying clustering and neighborhood construction are often difficult to verify directly. ClusterGraph is built on top of an input partition of the data into clusters, and therefore its quality necessarily depends on the quality of that partition.
In particular, if the clustering is of poor quality, this can already be detected at the level of the partition itself. When a metric or similarity measure is available, one can assess this indirectly through, for example, large within-cluster diameters. In such a case, points assigned to the same cluster are identified at distance zero in the ClusterGraph representation, despite being far apart in the original space, which leads to a large distortion already at the partition level. Importantly, this acts as a warning signal: rather than silently producing a misleading summary, the method indicates that the chosen clustering should be reconsidered.
To fully address this comment, we have added Section 3.5 “ClusterGraph stability” to our manuscript, where we discuss the sensitivity of our method to the choice of k in k-NN, to the clustering granularity and its robustness to noise.

************* Reviewer
2.The proposed distortion metric requires pairwise shortest-path computations between clusters and averaging over all inter-cluster point pairs. This raises concerns about scalability to large datasets.
************* End Reviewer

We thank the Reviewer for raising this important point. We agree that the distortion measure, in its most direct form, may be computationally demanding on large datasets, since it relies on shortest-path distances in the underlying neighborhood graph together with averaging over inter-cluster point pairs.
We would like to emphasize that this all-to-all distance construction should be viewed primarily as an ideal reference object. In practice, it is expensive for several reasons. First, it requires constructing the k-NN graph itself, which may already be nontrivial in high dimensions. Second, computing shortest-path distances between all relevant pairs is costly, especially when the ambient dimension is high and exact nearest-neighbor search becomes difficult. For this reason, the exact distortion is not intended as the only practical route in large-scale settings, but rather as a quantity that can be approximated or accelerated.

To address these concerns, we have added Section 2.5 “ClusterGraph scalability”, where we discuss several ways to improve scalability. These include the use of approximate k-NN algorithms, dimensionality reduction as a preprocessing step, and replacing full pairwise averaging by simpler cluster representatives such as centroids. We also describe a landmark-based approximation, which in our view provides the most effective speed-up. More generally, most of the required computations can also be parallelized effectively.

************* Reviewer
3.The experimental section relies heavily on qualitative visual comparisons. While illustrative, these examples do not convincingly demonstrate that ClusterGraph improves downstream tasks, e.g., clustering. Some relevant references are:CDC: A Simple Framework for Complex Data Clustering;Structured Graph Learning for Scalable Subspace Clustering: From Single-view to Multi-view.
************* End Reviewer

We thank the Reviewer for this important comment. We agree that the current experimental section is primarily qualitative. At the same time, we would like to clarify the scope of the method. ClusterGraph is not proposed as a new clustering algorithm, and it is not merely a visualization method in the usual sense. Rather, it is a graph-based abstraction built on top of a given clustering, whose purpose is both to summarize the global organization of the data and to compress the dataset into a more compact structural representation. For this reason, a direct one-to-one comparison with methods such as PCA, t-SNE, or UMAP is inherently difficult: these methods return pointwise low-dimensional embeddings, whereas ClusterGraph produces a compressed graph-level representation of the clustered data.
This is why we have chosen to include in the manuscript, in addition to real-word examples, experiments with artificial datasets for which the underlying metric structure is known (e.g. Fig. 3 concentric circles). This ground truth knowledge allows us to qualitatively assess faithfulness of the ClusterGraph representation. Additional toy examples are provided in our GitHub repository.

************* Reviewer
4.Typographical and grammatical issues are rare but present (e.g., "two bridge this two approaches" in the Introduction).
************* End Reviewer

Thank you for the comment, we have run a spellchecker on the paper.

****

************* Reviewer
Reviewer #2: This manuscript proposes "ClusterGraph," a new tool designed to address the limitations of existing high-dimensional data analysis methods. Current dimensionality reduction techniques (e.g., t-SNE, UMAP), while effective at preserving local features, often lose the global structure of the dataset. Conversely, clustering algorithms partition data but fail to provide information regarding the inter-cluster organization. ClusterGraph bridges this gap by constructing a graph layer on top of the output of any clustering algorithm. In this graph, vertices represent clusters, and edges represent the distances in the ambient space. The authors introduce the concept of "Metric Distortion" to assess the consistency between the graph representation and the intrinsic geometric structure of the data. Furthermore, pruning strategies based on metric distortion and connectivity are proposed to optimize visualization. The authors demonstrate the effectiveness of this tool in capturing global structure, data compression, and visualization using synthetic datasets (concentric circles) and real-world biological datasets (mice protein expression, bone marrow mononuclear cells, and lung cancer cell lines). An open-source Python implementation is also provided.

Strengths:

1. Innovation and Complementarity: The method cleverly leverages ideas from Topological Data Analysis (TDA) to provide an intermediate representation between dimensionality reduction and pure clustering. It does not attempt to replace t-SNE or UMAP but offers an "embedding-agnostic" alternative. By encoding distances directly on graph edges rather than forcing a projection into a low-dimensional Euclidean space, ClusterGraph avoids the distance distortions inherent in standard embedding techniques.

2. Rigorous Theoretical Foundation: Unlike many purely heuristic tools, this work is supported by rigorous mathematical definitions and proofs. Specifically, Propositions 1 and 2 provide proofs for the stability of the method under changes in partitions. The use of the k-nearest neighbor graph to estimate intrinsic geodesic distances is well-grounded, citing relevant theorems by Bernstein et al.

3. Objective Quality Assessment: The introduction of "Metric Distortion" is a significant contribution. It allows users to quantitatively evaluate how well the ClusterGraph preserves the intrinsic structure of the data. This metric serves as an objective criterion for automated edge pruning, removing "shortcuts" to better reflect the underlying manifold.

4. Comprehensive and Intuitive Experimental Validation: The synthetic data experiments intuitively demonstrate the limitations of t-SNE and UMAP in preserving global distance ratios, while ClusterGraph captures them accurately. In real-world biological datasets, the authors successfully show how the tool reveals global relationships—such as the outlier status of specific clusters in the lung cancer dataset—that may be obscured or ambiguous in UMAP embeddings.
************* End Reviewer

We sincerely thank the Reviewer for their thorough and generous summary of our work.

************* Reviewer
Weaknesses and Suggestions:

1. Parameter Sensitivity Analysis: The construction of ClusterGraph relies on several parameters, including the choice of the clustering algorithm (e.g., k in k-means or resolution in Leiden), the k value for the k-NN graph used in intrinsic distance estimation, and the pruning thresholds. While different parameter values are used across the experiments, a systematic sensitivity analysis is missing. A discussion on how variations in these parameters—particularly the granularity of the clustering—affect the final graph structure and metric distortion score would be beneficial.
************* End Reviewer

We thank the Reviewer for this important comment. We agree that parameter sensitivity deserves a more systematic discussion. In the revised manuscript, we now address this point in Section 3.5 “ClusterGraph stability”. We include

additional quantitative experiments showing how the metric distortion changes with (i) the number of clusters used in the clustering stage, (ii) the neighborhood size k used in the k-NN graph for intrinsic-distance approximation, and (iii) added noise within clusters.

*************Reviewer
2. Visualization Scalability: While ClusterGraph aims to "compress" data for visualization, the examples provided utilize a relatively small number of clusters (approximately 10-20). For complex datasets requiring a significantly higher number of clusters (e.g., n > 50), the fully connected graph (even after pruning) might become visually cluttered. It would be helpful to discuss the readability and limitations of the visualization when the node count increases.
************* End Reviewer

We thank the Reviewer for this important comment. We agree that readability becomes a genuine issue when the number of nodes increases substantially. The current examples were intentionally kept in the range of approximately 10–20 clusters in order to make the visual message as clear as possible. For substantially larger numbers of clusters, a flat visualization of the full ClusterGraph may indeed become cluttered, even after pruning.
At the same time, we would like to emphasize that ClusterGraph should not be viewed only as a static visualization object. It is also a data-compression and structural-abstraction tool. From this perspective, when the number of clusters becomes too large for a single readable view, the natural next step is not necessarily to display all clusters at once, but rather to move to a hierarchical or multiscale representation.
We also note that the computational side of graph visualization at these sizes is not the main bottleneck. Modern graph-visualization platforms such as Gephi are specifically designed for interactive exploration of large networks, with millions of nodes and edges.

*************Reviewer
3. Computational Complexity: The manuscript would benefit from a discussion on computational costs. Calculating inter-cluster distances (e.g., Wasserstein distance) and the metric distortion for all pairs can be computationally intensive. A brief analysis of the time complexity and scalability of the pipeline on large-scale datasets (e.g., single-cell data with millions of cells) is recommended.
*************End Reviewer

We thank the Reviewer for raising this important point. We agree that the full pipeline may be computationally demanding on large datasets, and we have expanded the discussion of computational aspects in Section 2.5 and in Appendix A.
To address scalability, we discuss several practical strategies in the manuscript. These include the use of approximate k-NN algorithms, dimensionality reduction as a preprocessing step, and replacing full pairwise averaging by simpler cluster representatives such as centroids. We also describe a landmark-based approximation, which in our view provides the most effective speed-up. More generally, most of the required computations can also be parallelized effectively.

************* Reviewer

Cookie Preference Center

We use cookies which are necessary to make our site work. We may also use additional cookies to analyze, improve and personalize our content and your digital experience. For more information, see our Cookie Policy.

You may choose not to allow some types of cookies. However, blocking some types may impact your experience of our site and the services we are able to offer. See the different category headings below to find out more or change your settings.

Allow all

Manage Consent Preferences

Strictly Necessary Cookies

Always active

These cookies are necessary for the website to function and cannot be switched off in our systems. They are usually only set in response to actions made by you which amount to a request for services, such as setting your privacy preferences, logging in or filling in forms. You can set your browser to block or alert you about these cookies, but some parts of the site will not then work.

Functional Cookies

These cookies enable the website to provide enhanced functionality and personalisation. They may be set by us or by third party providers whose services we have added to our pages. If you do not allow these cookies then some or all of these services may not function properly.

Performance Cookies

These cookies allow us to count visits and traffic sources so we can measure and improve the performance of our site. They help us to know which pages are the most and least popular and see how visitors move around the site.

Targeting Cookies

These cookies may be set through our site by our advertising partners. They may be used by those companies to build a profile of your interests and show you relevant adverts on other sites. If you do not allow these cookies, you will experience less targeted advertising.

Cookie List

Clear

Apply Cancel

Consent Leg.Interest

Confirm my choices

Powe[illegible]
