# Supplementary material for: ClusterGraph: a new tool for visualisation and compression of multidimensional data
Source: Gigascience. 2026 Jun 13;15:giag070. doi: 10.1093/gigascience/giag070 (PMC13354933; doi:10.1093/gigascience/giag070)
Supplement: giag070_Reviewer_1_Report_original_submission — Reviewer 1 -- 12/27/2025 [file giag070_reviewer_1_report_original_submission.pdf]

Reviewer Report

Title: ClusterGraph: a new tool for visualisation and compression of multidimensional data

Version: Original Submission **Date:** 12/27/2025

Reviewer name: Reviewer 1

Reviewer Comments to Author:

This manuscript introduces ClusterGraph, a graph-based abstraction built on top of clustering results, aiming to capture and visualize the global organization of high-dimensional data while avoiding distortions inherent to low-dimensional embeddings.
1.In realistic high-dimensional biological or social datasets, many assumptions are rarely verifiable. The manuscript would benefit from a discussion of failure modes, robustness to noisy clustering, and sensitivity to the choice of k in k-NN graphs.
2.The proposed distortion metric requires pairwise shortest-path computations between clusters and averaging over all inter-cluster point pairs. This raises concerns about scalability to large datasets.
3.The experimental section relies heavily on qualitative visual comparisons. While illustrative, these examples do not convincingly demonstrate that ClusterGraph improves downstream tasks, e.g., clustering. Some relevant references are:CDC: A Simple Framework for Complex Data Clustering;Structured Graph Learning for Scalable Subspace Clustering: From Single-view to Multi-view.
4.Typographical and grammatical issues are rare but present (e.g., "two bridge this two approaches" in the Introduction).

Methods

Are the methods appropriate to the aims of the study, are they well described, and are necessary controls included? Choose an item.

Conclusions

Are the conclusions adequately supported by the data shown? Choose an item.

Reporting Standards

Does the manuscript adhere to the journal’s guidelines on [minimum standards of reporting?]() Choose an item.

Choose an item.

Statistics

Are you able to assess all statistics in the manuscript, including the appropriateness of statistical tests used? Choose an item.

Quality of Written English

Please indicate the quality of language in the manuscript: Choose an item.

Declaration of Competing Interests

Please complete a declaration of competing interests, considering the following questions:

- Have you in the past five years received reimbursements, fees, funding, or salary from an organisation that may in any way gain or lose financially from the publication of this manuscript, either now or in the future?
- Do you hold any stocks or shares in an organisation that may in any way gain or lose financially from the publication of this manuscript, either now or in the future?
- Do you hold or are you currently applying for any patents relating to the content of the manuscript?
- Have you received reimbursements, fees, funding, or salary from an organization that holds or has applied for patents relating to the content of the manuscript?
- Do you have any other financial competing interests?
- Do you have any non-financial competing interests in relation to this paper?

If you can answer no to all of the above, write 'I declare that I have no competing interests' below. If your reply is yes to any, please give details below.

I declare that I have no competing interests

I agree to the open peer review policy of the journal. I understand that my name will be included on my report to the authors and, if the manuscript is accepted for publication, my named report including any attachments I upload will be posted on the website along with the authors' responses. I agree for my report to be made available under an Open Access Creative Commons CC-BY license (http://creativecommons.org/licenses/by/4.0/). I understand that any comments which I do not wish to be included in my named report can be included as confidential comments to the editors, which will not be published.

Choose an item.

To further support our reviewers, we have joined with Publons, where you can gain additional credit to further highlight your hard work (see: https://publons.com/journal/530/gigascience). On publication of this paper, your review will be automatically added to Publons, you can then choose whether or not to claim your Publons credit. I understand this statement.

Yes Choose an item.
