# Supplementary material for: ClusterGraph: a new tool for visualisation and compression of multidimensional data
Source: Gigascience. 2026 Jun 13;15:giag070. doi: 10.1093/gigascience/giag070 (PMC13354933; doi:10.1093/gigascience/giag070)
Supplement: giag070_Reviewer_2_Report_original_submission — Reviewer 2 -- 1/5/2026 [file giag070_reviewer_2_report_original_submission.pdf]

Reviewer Report

Title: ClusterGraph: a new tool for visualisation and compression of multidimensional data

Version: Original Submission **Date:** 1/5/2026

Reviewer name: Reviewer 2

Reviewer Comments to Author:

This manuscript proposes "ClusterGraph," a new tool designed to address the limitations of existing high-dimensional data analysis methods. Current dimensionality reduction techniques (e.g., t-SNE, UMAP), while effective at preserving local features, often lose the global structure of the dataset. Conversely, clustering algorithms partition data but fail to provide information regarding the inter-cluster organization. ClusterGraph bridges this gap by constructing a graph layer on top of the output of any clustering algorithm. In this graph, vertices represent clusters, and edges represent the distances in the ambient space. The authors introduce the concept of "Metric Distortion" to assess the consistency between the graph representation and the intrinsic geometric structure of the data. Furthermore, pruning strategies based on metric distortion and connectivity are proposed to optimize visualization. The authors demonstrate the effectiveness of this tool in capturing global structure, data compression, and visualization using synthetic datasets (concentric circles) and real-world biological datasets (mice protein expression, bone marrow mononuclear cells, and lung cancer cell lines). An open-source Python implementation is also provided.

Strengths:

1. Innovation and Complementarity: The method cleverly leverages ideas from Topological Data Analysis (TDA) to provide an intermediate representation between dimensionality reduction and pure clustering. It does not attempt to replace t-SNE or UMAP but offers an "embedding-agnostic" alternative. By encoding distances directly on graph edges rather than forcing a projection into a low-dimensional Euclidean space, ClusterGraph avoids the distance distortions inherent in standard embedding techniques.
2. Rigorous Theoretical Foundation: Unlike many purely heuristic tools, this work is supported by rigorous mathematical definitions and proofs. Specifically, Propositions 1 and 2 provide proofs for the stability of the method under changes in partitions. The use of the k-nearest neighbor graph to estimate intrinsic geodesic distances is well-grounded, citing relevant theorems by Bernstein et al.
3. Objective Quality Assessment: The introduction of "Metric Distortion" is a significant contribution. It allows users to quantitatively evaluate how well the ClusterGraph preserves the intrinsic structure of the data. This metric serves as an objective criterion for automated edge pruning, removing "shortcuts" to better reflect the underlying manifold.
4. Comprehensive and Intuitive Experimental Validation: The synthetic data experiments intuitively demonstrate the limitations of t-SNE and UMAP in preserving global distance ratios, while ClusterGraph captures them accurately. In real-world biological datasets, the authors successfully show how the tool reveals global relationshipsâ€”such as the outlier status of specific clusters in the lung cancer datasetâ€”that may be obscured or ambiguous in UMAP embeddings.

Weaknesses and Suggestions:
1. Parameter Sensitivity Analysis: The construction of ClusterGraph relies on several parameters, including the choice of the clustering algorithm (e.g., k in k-means or resolution in Leiden), the k value for the k-NN graph used in intrinsic distance estimation, and the pruning thresholds. While different parameter values are used across the experiments, a systematic sensitivity analysis is missing. A discussion on how variations in these parametersâ€”particularly the granularity of the clusteringâ€”affect the final graph structure and metric distortion score would be beneficial.
2. Visualization Scalability: While ClusterGraph aims to "compress" data for visualization, the examples provided utilize a relatively small number of clusters (approximately 10-20). For complex datasets requiring a significantly higher number of clusters (e.g., n > 50), the fully connected graph (even after pruning) might become visually cluttered. It would be helpful to discuss the readability and limitations of the visualization when the node count increases.
3. Computational Complexity: The manuscript would benefit from a discussion on computational costs. Calculating inter-cluster distances (e.g., Wasserstein distance) and the metric distortion for all pairs can be computationally intensive. A brief analysis of the time complexity and scalability of the pipeline on large-scale datasets (e.g., single-cell data with millions of cells) is recommended.

Methods

Are the methods appropriate to the aims of the study, are they well described, and are necessary controls included? Choose an item.

Conclusions

Are the conclusions adequately supported by the data shown? Choose an item.

Reporting Standards

Does the manuscript adhere to the journal's guidelines on [minimum standards of reporting?]() Choose an item.

Choose an item.

Statistics

Are you able to assess all statistics in the manuscript, including the appropriateness of statistical tests used? Choose an item.

Quality of Written English

Please indicate the quality of language in the manuscript: Choose an item.

Declaration of Competing Interests

Please complete a declaration of competing interests, considering the following questions:

- Have you in the past five years received reimbursements, fees, funding, or salary from an organisation that may in any way gain or lose financially from the publication of this manuscript, either now or in the future?
- Do you hold any stocks or shares in an organisation that may in any way gain or lose financially from the publication of this manuscript, either now or in the future?
- Do you hold or are you currently applying for any patents relating to the content of the manuscript?
- Have you received reimbursements, fees, funding, or salary from an organization that holds or has applied for patents relating to the content of the manuscript?
- Do you have any other financial competing interests?
- Do you have any non-financial competing interests in relation to this paper?

If you can answer no to all of the above, write 'I declare that I have no competing interests' below. If your reply is yes to any, please give details below.

I declare that I have no competing interests.

I agree to the open peer review policy of the journal. I understand that my name will be included on my report to the authors and, if the manuscript is accepted for publication, my named report including any attachments I upload will be posted on the website along with the authors' responses. I agree for my report to be made available under an Open Access Creative Commons CC-BY license (http://creativecommons.org/licenses/by/4.0/). I understand that any comments which I do not wish to be included in my named report can be included as confidential comments to the editors, which will not be published.

Choose an item.

To further support our reviewers, we have joined with Publons, where you can gain additional credit to further highlight your hard work (see: https://publons.com/journal/530/gigascience). On publication of this paper, your review will be automatically added to Publons, you can then choose whether or not to claim your Publons credit. I understand this statement.

Yes Choose an item.
